# NS1 Protein Mutation I64T Affects Interferon Responses and Virulence of Circulating H3N2 Human Influenza A Viruses

**DOI:** 10.1128/JVI.01039-16

**Published:** 2016-10-14

**Authors:** Marta L. DeDiego, Aitor Nogales, Kris Lambert-Emo, Luis Martinez-Sobrido, David J. Topham

**Affiliations:** a David H. Smith Center for Vaccine Biology and Immunology, University of Rochester, Rochester, New York, USA; b Department of Microbiology and Immunology, University of Rochester, Rochester, New York, USA; St. Jude Children's Research Hospital

## Abstract

Influenza NS1 protein is the main viral protein counteracting host innate immune responses, allowing the virus to efficiently replicate in interferon (IFN)-competent systems. In this study, we analyzed NS1 protein variability within influenza A (IAV) H3N2 viruses infecting humans during the 2012-2013 season. We also evaluated the impact of the mutations on the ability of NS1 proteins to inhibit host innate immune responses and general gene expression. Surprisingly, a previously unidentified mutation in the double-stranded RNA (dsRNA)-binding domain (I64T) decreased NS1-mediated general inhibition of host protein synthesis by decreasing its interaction with cleavage and polyadenylation specificity factor 30 (CPSF30), leading to increased innate immune responses after viral infection. Notably, a recombinant A/Puerto Rico/8/34 H1N1 virus encoding the H3N2 NS1-T64 protein was highly attenuated in mice, most likely because of its ability to induce higher antiviral IFN responses at early times after infection and because this virus is highly sensitive to the IFN-induced antiviral state. Interestingly, using peripheral blood mononuclear cells (PBMCs) collected at the acute visit (2 to 3 days after infection), we show that the subject infected with the NS1-T64 attenuated virus has diminished responses to interferon and to interferon induction, suggesting why this subject could be infected with this highly IFN-sensitive virus. These data demonstrate the importance of influenza virus surveillance in identifying new mutations in the NS1 protein, affecting its ability to inhibit innate immune responses and, as a consequence, the pathogenicity of the virus.

**IMPORTANCE** Influenza A and B viruses are one of the most common causes of respiratory infections in humans, causing 1 billion infections and between 300,000 and 500,000 deaths annually. Influenza virus surveillance to identify new mutations in the NS1 protein affecting innate immune responses and, as a consequence, the pathogenicity of the circulating viruses is highly relevant. Here, we analyzed amino acid variability in the NS1 proteins from human seasonal viruses and the effect of the mutations in innate immune responses and virus pathogenesis. A previously unidentified mutation in the dsRNA-binding domain decreased NS1-mediated general inhibition of host protein synthesis and the interaction of the protein with CPSF30. This mutation led to increased innate immune responses after viral infection, augmented IFN sensitivity, and virus attenuation in mice. Interestingly, using PBMCs, the subject infected with the virus encoding the attenuating mutation induced decreased antiviral responses, suggesting why this subject could be infected with this virus.

## INTRODUCTION

Influenza A (IAV) and B (IBV) viruses are members of the Orthomyxoviridae family responsible for seasonal epidemics and occasional pandemics, being one of the most common causes of respiratory infections in humans (1). IAVs are further classified in different subtypes according to the two major surface glycoproteins, hemagglutinin (HA; 1 to 17) and neuraminidase (NA; 1 to 9). In humans, the most frequent seasonal subtypes of IAVs are H3N2 and H1N1, and there are two lineages (Yamagata and Victoria) of IBVs. Seasonal IAV H1N1 and H3N2 have been cocirculating since 1977 (1). Despite comprehensive vaccination programs, the WHO estimates that the global disease burden from seasonal influenza results in 1 billion infections, with 3 to 5 million cases of severe disease and between 300,000 and 500,000 deaths annually (2).

The defense mechanisms provided by the innate immune system restrict influenza virus replication (3). Virus-specific patterns (pathogen-associated molecular patterns [PAMPs]) are recognized in infected cells by pattern recognition receptors (PRRs), which initiate signaling pathways leading to the production of type I and III interferons (IFNs) and proinflammatory cytokines (3). Influenza virus is recognized by the membrane-associated PRR Toll-like receptors (TLRs) 3 (double-stranded RNA [dsRNA]), 7, and 8 (single-stranded RNA [ssRNA]) and by the cytoplasmic PRR retinoic acid-inducible gene I (RIG-I) and the NOD-like receptor family member LRR and pyrin domain containing-3 (NLRP3) (3). The consequence of these downstream signaling pathways is the activation of transcription factors, such as interferon-regulatory factor 3 (IRF3), NF-κB, and activating transcription factor (ATF)-2/c-Jun, which are responsible for the transcription of type I (IFN-α and IFN-β) and type III (IFN-λ) IFNs (3–5). Secreted type I and III IFNs act in a paracrine and/or autocrine fashion and induce the expression of hundreds of IFN-stimulated genes (ISGs), many of which possess antiviral activity (3, 5, 6).

Influenza virus NS1 protein allows the virus to replicate efficiently by suppressing the host innate immune responses by a variety of mechanisms (reviewed in reference 7). Accordingly, influenza viruses lacking the NS1 protein or expressing reduced levels or truncated forms of the NS1 protein are severely impaired in growth in type I IFN-competent cells and only show pathogenicity in mice lacking key components of the type I IFN pathway (8–11). These results indicate that influenza NS1 protein is the main viral protein involved in type I IFN antagonism.

Mechanisms involved in the NS1 protein's ability to counteract innate immune responses include the inhibition of cellular transcription elongation, blocking posttranscriptional RNA processing and nuclear export, decreasing retinoic acid-inducible gene 1 (RIG-I) activation through sequestration of this RNA helicase and its activating ligand (12–14) or through inhibition of tripartite motif family 25 (TRIM25)-mediated RIG-I ubiquitination (15), interfering with IFN signaling, and directly inhibiting specific ISGs, such as protein kinase R (PKR) and RNase L, by binding to dsRNA and PKR directly (reviewed in reference 7). In addition, NS1 proteins from some human IAVs bind to the 30-kDa subunit of cleavage and polyadenylation specificity factor 30 (CPSF30), blocking the processing of cellular mRNAs (16–19). The IAV NS1 binding site for CPSF30 is centered around amino acid 186. In addition, amino acid residues 103 and 106 (16, 17, 20, 21) as well as 108, 125, and 189 (22) are important for NS1 binding. IAV NS1 protein also binds to the poly(A)-binding protein II (PABPII), inhibiting the ability of PABPII to stimulate the synthesis of long poly(A) tails (23). These two mechanisms block pre-mRNA processing and the nuclear export of mRNAs (24), leading to a general inhibition of host gene expression.

While much is known about the anti-type I IFN function of NS1 proteins derived from avian and some pandemic IAVs (17, 21, 22, 25), less is known about the diversity of NS1 in seasonal human influenza viruses and its possible contribution to disease severity. In this paper, we analyzed the NS1 protein sequences of IAV circulating within the 2012-2013 season in the Rochester (NY) area using nasal washes/swabs of different enrolled subjects. The effect of the mutations found (E26K, I64T, and R224K) on the ability of the NS1 protein to inhibit the type I IFN responses and host gene expression was analyzed. A mutation at position 64 affected the ability of NS1 to inhibit host gene expression. The lack of inhibition of host gene expression was due at least in part to the lack of interaction with CPSF30. Moreover, I64T substitution in NS1 affected virus growth, the ability to inhibit induction of type I IFN *in vitro* and *in vivo*, the sensitivity of the virus to the antiviral effect of type I IFN, and virus pathogenesis in mice. This is, to our knowledge, the first time that a mutation in the dsRNA binding domain of IAV NS1 affects CPSF30 binding and host protein expression, including the antiviral action of type I IFN. Moreover, a virus containing the I64T mutation resulted in an attenuated phenotype in mice. Furthermore, PBMCs isolated from the subject infected with the H3N2 IAV encoding the T64-NS1 protein showed evidence of a defect in the induction of innate antiviral responses. This deficiency in the ability to mount an effective antiviral response may account for the ability of the IFN-deficient virus to efficiently replicate in this and possibly other similar subjects.

## MATERIALS AND METHODS

### Cells.

Human embryonic kidney (293T; ATCC CRL-11268), human lung epithelial carcinoma (A549; ATCC CCL-185), African green monkey kidney epithelial (Vero; ATCC CCL-81), and canine kidney epithelial (MDCK; ATCC CCL-34) cells were grown at 37°C in air enriched with 5% CO_2_ using Dulbecco's modified Eagle's medium (DMEM; Gibco) supplemented with 10% fetal bovine serum (Gibco), 100 U/ml penicillin, 0.1 mg/ml streptomycin, and 50 μg/ml gentamicin (Gibco).

### Plasmids.

Polymerase II expression pCAGGS plasmids (26) containing NS1 sequences fused to an HA epitope tag (YPYDVPDYA) at the N terminus (13) were obtained using the oligonucleotides indicated in Table 1. To abolish NS mRNA splicing, two silent mutations were introduced at nucleotides (nt) 501 and 504 of NS1 (highlighted in boldface in Table 1). RNAs were extracted from nasal washes/swabs from patients 54FUR0055 (designated 55), 54FUR0065 (65), 54FUR0085 (85), and 54FUR0087 (87). Reverse transcription (RT) reactions were performed at 37°C for 2 h using the high-capacity cDNA transcription kit (Applied Biosystems) and an oligo(dT) primer to obtain the complementary DNAs (cDNAs). Overlapping PCRs using the Pfx polymerase (Invitrogen) and primers and template cDNAs described in Table 1 were used to amplify the viral NS1 protein. PCR products were cloned in the pCAGGS-HA NH2 expression plasmid (13) using SphI and NheI restriction enzymes. Plasmids (pCAGGS) encoding the NS1 proteins of influenza A/PuertoRico/8/34 (PR8) and influenza A/BrevigMission/01/1918 (1918) H1N1 viruses were previously described (13, 17).

**TABLE 1:** Primers and templates used to clone NS1 proteins in pCAGGS-HA-NH_2_ plasmid

Mutations (cDNA template) and PCR round	Primer	Sequence^*a*^ (5′–3′)
K26, I64, K224 (virus 87)		
1	NS1-SphI-VS	CTCCGATGAGGCATGCAGATTCCAACACTGTGTCAAGTTTCCAGG
	NS1-mutsplic-RS	GACATCCTCAATAGTATG**C**CC**G**GGAAAAGAAGGCAATGG
2	NS1-mutsplic-VS	CCATTGCCTTCTTTTCC**C**GG**G**CATACTATTGAGGTGTC
	NS1-NheI-K224-RS	GGACCTACCGCTAGCTCAAACTTTTGACCTAGCTGTTTTCGCC
E26, I64, K224 (virus 65)		
1	NS1-SphI-VS	CTCCGATGAGGCATGCAGATTCCAACACTGTGTCAAGTTTCCAGG
	NS1-mutsplic-RS	GACATCCTCAATAGTATG**C**CC**G**GGAAAAGAAGGCAATGG
2	NS1-mutsplic-VS	CCATTGCCTTCTTTTCC**C**GG**G**CATACTATTGAGGTGTC
	NS1-NheI-K224-RS	GGACCTACCGCTAGCTCAAACTTTTGACCTAGCTGTTTTCGCC
K26, T64, K224 (virus 85)		
1	NS1-SphI-VS	CTCCGATGAGGCATGCAGATTCCAACACTGTGTCAAGTTTCCAGG
	NS1-mutsplic-RS	GACATCCTCAATAGTATG**C**CC**G**GGAAAAGAAGGCAATGG
2	NS1-mutsplic-VS	CCATTGCCTTCTTTTCC**C**GG**G**CATACTATTGAGGTGTC
	NS1-NheI-K224-RS	GGACCTACCGCTAGCTCAAACTTTTGACCTAGCTGTTTTCGCC
E26, T64, K224 (virus 85)		
1	NS1-SphI-E26-VS	CTCCGATGAGGCATGCAGATTCCAACACTGTGTCAATTTTCCAGGTAGATTGCTTTCTTTGGCATATCCGGAAACAAGTTGTAGACCAAGAACTGAGCGATGCCCC
	NS1-mutsplic-RS	GACATCCTCAATAGTATG**C**CC**G**GGAAAAGAAGGCAATGG
2	NS1-mutsplic-VS	CCATTGCCTTCTTTTCC**C**GG**G**CATACTATTGAGGTGTC
	NS1-NheI-K224-RS	GGACCTACCGCTAGCTCAAACTTTTGACCTAGCTGTTTTCGCC
K26, I64, R224 (virus 65)		
1	NS1-SphI-VS	CTCCGATGAGGCATGCAGATTCCAACACTGTGTCAAGTTTCCAGG
	NS1-mutsplic-RS	GACATCCTCAATAGTATG**C**CC**G**GGAAAAGAAGGCAATGG
2	NS1-mutsplic-VS	CCATTGCCTTCTTTTCC**C**GG**G**CATACTATTGAGGTGTC
	NS1-NheI-R224-RS	GGACCTACCGCTAGCTCAAACTTTTGACCTAGCTGTTCTCGCC
E26, I64, R224 (virus 55)		
1	NS1-SphI-VS	CTCCGATGAGGCATGCAGATTCCAACACTGTGTCAAGTTTCCAGG
	NS1-mutsplic-RS	GACATCCTCAATAGTATG**C**CC**G**GGAAAAGAAGGCAATGG
2	NS1-mutsplic-VS	CCATTGCCTTCTTTTCC**C**GG**G**CATACTATTGAGGTGTC
	NS1-NheI-R224-RS	GGACCTACCGCTAGCTCAAACTTTTGACCTAGCTGTTCTCGCC
K26, T64, R224 (virus 85)		
1	NS1-SphI-VS	CTCCGATGAGGCATGCAGATTCCAACACTGTGTCAAGTTTCCAGG
	NS1-mutsplic-RS	GACATCCTCAATAGTATG**C**CC**G**GGAAAAGAAGGCAATGG
2	NS1-mutsplic-VS	CCATTGCCTTCTTTTCC**C**GG**G**CATACTATTGAGGTGTC
	NS1-NheI-R224-RS	GGACCTACCGCTAGCTCAAACTTTTGACCTAGCTGTTCTCGCC
E26, T64, R224 (virus 85)		
1	NS1-SphI-E26-VS	CTCCGATGAGGCATGCAGATTCCAACACTGTGTCAATTTTCCAGGTAGATTGCTTTCTTTGGCATATCCGGAAACAAGTTGTAGACCAAGAACTGAGCGATGCCCC
	NS1-mutsplic-RS	GACATCCTCAATAGTATG**C**CC**G**GGAAAAGAAGGCAATGG
2	NS1-mutsplic-VS	CCATTGCCTTCTTTTCC**C**GG**G**CATACTATTGAGGTGTC
	NS1-NheI-R224-RS	GGACCTACCGCTAGCTCAAACTTTTGACCTAGCTGTTCTCGCC

a Boldface nucleotides indicate silent mutations introduced to abolish NS mRNA splicing.

To introduce mutations E26K, I64T, and R224K into the NS1 protein of 1918 NS1, overlapping PCRs were performed using the primers described in Table 2, using as the template the pCAGGS-HA NH2 NS1-1918 plasmid (17). PCR products were digested with SmaI and XhoI and cloned into pCAGGS-HA NH2 (13). The integrity of the amplified PCR product was confirmed by sequencing (Genewiz).

**TABLE 2:** Primers and templates used to introduce mutations in pCAGGS-HA2-NH2-NS1-1918

Mutation and PCR round	Primer	Sequence (5′–3′)
E26K		
1	NS1-1918-SmaI-VS	CGGATTCACCCGGGGATTCCAACACTGTGTCAAGCTTTCAGG
	NS1-1918-K26 -RS	GGGGCATCACCCAGTTTTTGGGTCTGCAAACCG
2	NS1-1918-K26-VS	CGGTTTGCAGACCCAAAAACTGGGTGATGCCCC
	NS1-1918-XhoI-RS	TGTCGGCTCGAGTCAAACTTCTGACTTAATTGTTCTCGCCATTT
I64T		
1	NS1-1918-SmaI-VS	CGGATTCACCCGGGGATTCCAACACTGTGTCAAGCTTTCAGG
	NS1-1918-T64 -RS	CTTCAGAATCCGCTCCACAGTCTGCTTTCCAGCACGGG
2	NS1-1918-T64 -VS	CCCGTGCTGGAAAGCAGACTGTGGAGCGGATTCTGAAG
	NS1-1918-XhoI-RS	TGTCGGCTCGAGTCAAACTTCTGACTTAATTGTTCTCGCCATTT
R224G		
1	NS1-1918-SmaI-VS	CGGATTCACCCGGGGATTCCAACACTGTGTCAAGCTTTCAGG
	NS1-1918-K224-XhoI-RS	CTGTCGGCTCGAGTCAAACTTCTGACTTAATTGTTTTCGCCATTTTCCG

Plasmids encoding NS1 variants under the control of the phage T7 polymerase were obtained by subcloning NS1 from the pCAGGS-HA NH2 plasmids into pcDNA3 plasmid using EcoRI and XbaI restriction enzymes. pcDNA3 plasmids encoding PR8 and 1918 NS1 proteins have been described previously (17).

The NS1 genes of IAV H3N2 85 and 87 were cloned in the previously described pDZ plasmid encoding nonoverlapping NS1 and NEP open reading frames (ORFs) (pDZ-NSs) (27). NS1 ORFs were amplified by PCR using primers NS1-EcoRI-H3N2-VS (5′-GGCTGAATTCGAGCTCGGTACCCGGGGATCCTCTAGACCGGAGTACTGGTCGACCTCCGAAGTTGGGGGGGAGCAAAAGCAGGGTGACAAAGACATAATGGATTCCAACACTGTGTCAAGC-3′, containing an EcoRI restriction site, the 5′ end of pDZ plasmid, the 5′ noncoding region [NCR], and first 24 nt of the NS1 ORF) and NS1-BsmBI-H3N2-RS (5′-CGCGCCGCTGCCAGAGACGCCAACTTCTGACCTAGCTGTTTTCGCC-3′, containing a BsmBI restriction site, the last 25 nt of the NS1 ORF, excluding the stop codon, and 18 nt of the porcine teschovirus [PTV] 2A autoproteolytic site). pCAGGS-HA NH2 plasmids encoding the different NS1 variants were used as templates. PCR products were digested with EcoRI and BsmBI restriction enzymes and cloned into the pDZ-NSs plasmid (27).

### Virus titrations.

IAV were titrated by immunofocus assay (fluorescent focus units [FFU] per milliliter) in MDCK cells. Briefly, confluent plates of MDCK cells (96-well format; 5 × 10^4^ cells/well) were infected with 10-fold serial dilutions of tissue culture supernatants (TCS). At 8 h postinfection (hpi), cells were fixed and permeabilized using 4% formaldehyde, 0.5% Triton X-100 in phosphate-buffered saline (PBS) for 20 min at room temperature. After washing with PBS, cells were incubated in blocking solution (2.5% bovine serum albumin [BSA] in PBS) for 1 h at room temperature, washed with PBS, and incubated with the IAV NP monoclonal antibody (MAb) NR 4282 (BEI Resources) diluted in 1% BSA for 2 h at 37°C. After washing with PBS, cells were incubated with a fluorescein isothiocyanate (FITC)-conjugated rabbit anti-mouse IgG secondary antibody (Dako) diluted 1:1,000 in 1% BSA for 1 h at 37°C. IAV NP-positive cells were visualized and enumerated to determine the virus titer (FFU per milliliter) using a fluorescence microscope. Viral infections were performed in the presence of 1 μg/ml of tosylsulfonyl phenylalanyl chloromethyl ketone (TPCK)-treated trypsin (Sigma).

Vesicular stomatitis virus expressing green fluorescent protein (VSV-GFP) (28) was titrated by plaque assay (PFU per milliliter) in Vero cells. Confluent cell monolayers (12-well format; 3.75× 10^5^ cells/well) were infected with 10-fold serial dilutions for 1 h at room temperature, overlaid with agar, and incubated at 37°C. Viral plaques were visualized and counted 1 day postinfection (dpi) using crystal violet.

### Virus rescue.

Cocultures (1:1) of 293T and MDCK cells in 6-well plates were cotransfected in suspension with 1 μg of the seven ambisense wild-type (WT) plasmids (pDZ-PB2, -PB1, -PA, -HA, -NP, -NA, and -M) of influenza A/Puerto Rico/8/34 H1N1 (kindly provided by A. Garcia-Sastre, Mount Sinai School of Medicine, NY) plus the ambisense split NS plasmids (pDZ-NSs) encoding the NS1 proteins of IAV 85 or 87 using DNA-IN (Molecular Transfer, Inc.) (29). At 12 h posttransfection (hpt), transfection medium was replaced with DMEM containing 0.3% BSA, antibiotics, and 1 μg/ml TPCK trypsin (Sigma). At 48 hpt, TCS were collected, clarified, and used to infect fresh MDCK cells. At 3 dpi, recombinant viruses were plaque purified and scaled up in MDCK cells. Virus stocks were generated by infecting confluent 10-cm dishes of MDCK cells at a low multiplicity of infection (MOI) (0.001). Stocks were titrated by immunofocus assay (FFU/ml) on MDCK cells. The identity of the NS1 ORFs in the rescued viruses was confirmed by restriction analysis and sequencing (Genewiz).

### Virus growth kinetics.

To determine virus growth rates *in vitro*, confluent monolayers of MDCK or A549 cells (24-well format; 2 × 10^5^ cells/well) were infected in duplicate at low multiplicity of infection (MOI, 0.001). After 1 h of virus adsorption at room temperature, cells were washed and overlaid with DMEM containing 0.3% BSA, antibiotics, and TPCK-treated trypsin (1 μg/ml for MDCK cells and 0.25 μg/ml for A549 cells). In the experiments to measure IFN sensitivity, the medium also contained 2,000 U/ml of universal IFN-α (Axxora). At the indicated times postinfection, TCS were collected and viral titers were determined by immunofocus assay (FFU/ml) as described above.

### Bioassay to assess IFN production.

To evaluate the effect of NS1 protein on the induction of IFN mediated by Sendai virus (SeV) infection, triplicate wells (96-well plate format; 6 × 10^4^ cells/well) of human 293T cells were cotransfected (using calcium phosphate) with 100 ng/well of the pCAGGS-HA NH2 NS1 plasmids (or the empty plasmid as a control), 50 ng/well of plasmids expressing Firefly luciferase (Fluc) under the control of IFN-β (pIFNB Fluc), or the IFN-stimulated response element (ISRE) (pISRE-Fluc) promoters (17), as well as 50 ng/well of a reporter plasmid carrying Renilla luciferase (Rluc) under the control of the constitutively active simian virus 40 (SV40) promoter (pRL-SV40) (Promega). At 24 hpt, cells were infected with SeV, Cantell strain (17), and 16 hpi cell culture supernatants were collected. Viruses in TCS were inactivated by exposure to shortwave (254 nm) UV radiation for 40 min at a distance of 6 cm (11). Fresh human A549 cells (96-well plate format; 6 × 10^4^ cells/well in triplicate) were treated with the UV-inactivated TCS for 24 h and then infected (MOI, 0.001) with VSV-GFP for 16 h (11). GFP expression was analyzed under a fluorescence microscope.

To measure the levels of IFN produced by infected cells, confluent monolayers of MDCK cells (24-well plate format; 5 × 10^4^ cells/well in triplicate) constitutively expressing GFP and Fluc reporter genes under the control of the IFN-β promoter (MDCK IFN-β-GFP-/IFN-β-Fluc) (30) were mock infected or infected (MOI, 4) with either r85 or r87 virus. At 12 hpi, activation of the IFN-β promoter was determined by assessing GFP expression under a fluorescence microscope or by quantifying Fluc expression in cell lysates using a Promega luciferase reporter assay and a Lumicount luminometer. Influenza virus infection levels were evaluated by immunofluorescence using an anti-NP MAb (HB-65) as described above. In addition, TCS of infected MDCK cells were collected and viruses were UV inactivated as previously described (11). Fresh MDCK cells seeded in 96-well plates were treated (in triplicate wells) with the UV-inactivated TCS for 24 h and then infected with Newcastle disease virus (NDV)-GFP (MOI, 5) (11). The GFP intensity was measured, at 14 hpi, with a microplate reader (DTX880; Beckman Coulter). GFP expression of mock-treated cells was considered to have a value of 100%. Mean values and standard deviations (SD) were calculated using Microsoft Excel software.

### Deep-sequencing analysis of transcriptomes and virus genes.

MDCK cells were mock infected or infected at an MOI of 0.01 with viruses obtained from subjects 65 and 85. At 16 hpi, total RNAs were extracted using an RNeasy minikit (Qiagen) and further purified using an oligo(dT) column to obtain the cellular and viral mRNAs by following the manufacturer's recommendations. The mRNAs were subjected to deep sequencing at the University of Rochester Genomics Research Center. Sequence reads were cleaned according to a rigorous preprocessing workflow using Trimmomatic version 0.32 before mapping them to the canFam3.1 reference genome using SHRiMP2.2.3. Cufflinks2.0.2 was then used in conjunction with the canFam3.1 annotated genes to perform differential expression analysis. Differentially expressed genes were classified according to Gene Ontology (GO) annotation using the DAVID software (31). Only genes with a *P* value of <0.05 and a fold change of >1.5 were considered in the analysis. All sequence reads that did not align to the canFam3.1 genome were used as input to the Trinity assembler for each sample individually. The resulting contigs from each assembly were then compared (BLASTN 2.2.29+) against an influenza A/New York/392/2004 (H3N2) (PRJNA15622) reference to identify contigs derived from IAV. All of the ORFs of IAV genes were sequenced. To analyze influenza virus single-nucleotide polymorphisms (SNPs), bwa-0.7.12, samtools-1.3.1, and bcftools-1.3 software programs were used.

### Quantitative RT-PCR (qRT-PCR) of MDCK-infected cells.

MDCK cells were mock infected or infected (MOI, 0.01) with viruses obtained from nasal washes/swabs from subjects 65 and 85, isolated from MDCK cells (32). At 24 hpi, total RNAs were extracted using an RNeasy minikit (Qiagen) by following the manufacturer's recommendations. Reverse transcription (RT) reactions were performed at 37°C for 2 h using the high-capacity cDNA transcription kit and random hexamer oligonucleotides to generate cDNAs. Quantitative PCRs (qPCRs) were performed using TaqMan IFN-β, IFN-induced protein with tetratricopeptide repeats 2 (IFIT2), and CXCL10 gene expression assays (Applied Biosystems) specific for the Canis familiaris (dog) genes (Cf03644503_s1, Cf02645026_m1, and Cf02622529_m1, respectively). Quantification was achieved using the 2^−ΔΔ*CT*^ method (33).

### Inhibition of host protein expression.

To evaluate the effect of IAV NS1 on host protein synthesis, confluent monolayers of human 293T cells (96-well plate format; 6 × 10^4^ cells/well in triplicate) were transiently cotransfected, using DNA-IN, with 200 ng/well of pCAGGS-HA NH2 NS1 expression plasmids, or the empty plasmid as a control, together with 50 ng/well of pCAGGS plasmids expressing GFP (17) and Gaussia luciferase (Gluc) (34). GFP and Gluc expression levels were measured 30 hpt using fluorescence microscopy and a Lumicount luminometer, respectively. For Gluc measurements, cell culture supernatants were mixed with an equal volume of Biolux Gaussia luciferase reagent (New England BioLabs).

### Western blotting.

Cells were lysed in passive lysis buffer (Promega), containing Laemmli sample buffer (Bio-Rad) and β-mercaptoethanol and then boiled for 5 min before running 10% SDS-PAGE gels. Proteins were transferred to nitrocellulose membranes (Bio-Rad) and detected by Western blotting. Primary rabbit anti-HA (NS1) and anti-FLAG (CPSF30) polyclonal antibodies diluted 1:1,000 (H6908 and F7425, respectively; Sigma-Aldrich), MAb anti-actin diluted 1:2,000 (A1978; Sigma-Aldrich), and MAb anti-ISG15 diluted 1:200 (sc-166755; Santa Cruz Biotechnologies) were used, followed by incubation with a 1:1,000 dilution of goat anti-rabbit and goat anti-mouse IgG antibodies conjugated to horseradish peroxidase (Sigma-Aldrich). Proteins were detected by chemiluminescence using the SuperSignal West Femto maximum-sensitivity substrate (Thermo Scientific) by following the manufacturer's recommendations.

### Inhibition of IFN-β and ISRE promoters.

To evaluate the effect of NS1 protein on the inhibition of IFN-β and ISRE promoters, triplicate wells of human 293T cells (96-well format; 6 × 10^4^ cells/well) were cotransfected with 100 ng/well of the pCAGGS-HA NH2 NS1 plasmids or the empty plasmid as a control, with 50 ng/well of plasmids expressing Fluc under the control of the IFN-β (pIFNB-Fluc) or the ISRE (pISRE-Fluc) promoters (17) and with 50 ng/well of a reporter plasmid carrying the Rluc gene under the control of the constitutively active SV40 promoter (pRL-SV40) (Promega) using the calcium phosphate method. At 24 hpt, cells were infected with SeV, Cantell strain (17), and at 16 hpi cells were lysed using 20 mM Tris-HCl (pH 7.4), 5 mM EDTA, 100 mM NaCl, 1% NP-40, and cOmplete protease inhibitor cocktail (Roche) for 30 min on ice. Cell lysates were clarified by centrifugation at 14,000 rpm for 10 min at 4°C, and an equal volume of luciferase reporter buffer (Promega) was added. Fluc protein expression levels were quantified in a Lumicount luminometer.

### Bioassay to determine NS1 IFN antagonism.

Triplicate wells of human A549 cells (96-well format; 3 × 10^4^ cells/well) were transiently transfected with 200 ng/well of the pCAGGS-HA NH2 NS1 plasmids, or the empty plasmid as control, using DNA-IN by following the manufacturer's instructions. At 24 hpt, cells were transfected with 100 ng of polyinosinic-poly(C) [poly(I·C); Sigma] using DNA-IN or were treated with 250 U/ml of universal IFN-α. Sixteen hours after poly(I·C) or IFN-α treatment, cells were infected (MOI, 0.001) with VSV-GFP (28). VSV-GFP titers in the culture supernatants were determined 21 hpi as described above.

### Interaction of NS1 proteins with CPSF30.

HA-tagged NS1 variants were synthesized *in vitro* using the pcDNA3 plasmids and the TNT7 transcription/translation kit (Promega) by following the manufacturer's recommendations. Human 293T cells (6-well format; 1.5 ×10^6^ cells/well) were transiently transfected with 8,000 ng of a pCAGGS plasmid expressing a FLAG-tagged version of the human CPSF30 (17). At 30 hpt, cells were lysed in 20 mM Tris-HCl (pH 7.5), 100 mM NaCl, 0.5 mM EDTA, 5% glycerol, and 1% Triton X-100 and supplemented with a cOmplete mini protease inhibitor cocktail (Pierce). Cleared cell lysates expressing FLAG-CPSF30 were incubated overnight at 4°C with the *in vitro*-synthesized NS1 proteins and 20 μl of an anti-FLAG affinity resin (Sigma-Aldrich). After extensive washing, precipitated proteins were dissociated from the resin using disruption buffer and analyzed by Western blotting as described above using rabbit anti-HA (NS1)- and anti-FLAG (CPSF30)-specific polyclonal antibodies.

### Interaction of NS1 proteins with dsRNA.

To prepare poly(I·C)-conjugated agarose beads, poly(C)-conjugated agarose beads (Sigma) containing approximately 62.5 μg of poly(C) were washed five times with TBS buffer (25 mM Tris, 150 mM NaCl). The beads then were resuspended in buffer containing 50 mM Tris and 50 mM NaCl and incubated overnight with 500 μg of inosinic acid (Sigma). The beads were washed twice with TBS, resuspended in TBS buffer containing 1 mM EDTA and 0.5% Triton X-100, and incubated at 4°C for 2 h with the HA-tagged NS1 variants synthesized *in vitro* using pcDNA3 plasmids and the TNT7 transcription/translation kit (Promega). The mixture was washed 3 times with TBS buffer containing 1 mM EDTA and 0.05% Tween 20, and the bound proteins were eluted in loading buffer at 95°C over 5 min. The eluted proteins were analyzed by Western blotting using a rabbit anti-HA-specific polyclonal antibody, as previously described.

### Mouse experiments.

Female 6-week-old C57BL/6 mice were purchased from the Jackson Laboratory and maintained in the animal care facility at the University of Rochester in a pathogen-free environment. All animal protocols were approved by the University of Rochester Committee of Animal Resources and complied with the recommendations in the *Guide for the Care and Use of Laboratory Animals* of the National Research Council (35). Mice were anesthetized intraperitoneally (i.p.) with 2,2,2-tribromoethanol (Avertin) and then inoculated intranasally (i.n.) with 30 μl of the indicated recombinant influenza A/Puerto Rico/8/34 NS split viruses encoding the NS1 proteins from patients 85 and 87 (r85 and r87, respectively). The mice were monitored daily for morbidity (body weight loss; *n* = 5) and mortality (survival; *n* = 5). Mice showing more than 25% loss of body weight were considered to have reached the experimental endpoint and were humanely euthanized.

Virus replication was evaluated by determination of viral titers in the lungs at 1, 3, and 5 dpi. To that end, mice (*n* = 3) were sacrificed and lungs were extracted and homogenized. Virus titers were determined by immunofocus assay (FFU per milliliter) as indicated above. Geometric mean titers (GMTs) were determined and statistical analyses (Mann-Whitney test) were performed using GraphPad Prism software. Levels of IFN-β induction were analyzed in mouse lungs at 1, 3, and 5 dpi. To that end, mice (*n* = 3) were sacrificed and lungs were extracted and incubated in RNAlater (Ambion) at 4°C for 24 h prior to freezing at −80°C. Lungs were homogenized in lysis buffer using a gentleMACS dissociator (Miltenyi Biotec), and total RNA was extracted using an RNeasy minikit (Qiagen). RT reactions were performed at 37°C for 2 h using the high-capacity cDNA transcription kit and random hexamer oligonucleotides to generate cDNAs. qPCRs were performed using a TaqMan gene expression assay (Applied Biosystems) specific for the IFN-β murine gene (Mm00439552_s1). Quantification was achieved using the 2^−ΔΔ*CT*^ method (33).

### Study design and human subjects.

Human subjects were enrolled as part of an acute influenza surveillance protocol (IRB number 09-0034). Individuals reporting influenza-like illness (fever, cough, and rhinitis) were asked to visit the Vaccine Research Unit (VRU) at the University of Rochester for nasal and nasopharyngeal swabbing and blood sampling. Starting with nasopharyngeal swabs, MDCK cells were inoculated and observed for cytopathic effect at 3 dpi. Supernatants then were collected and used to generate virus stocks in MDCK cells (32). The study was approved by the University of Rochester Human Research Subjects Review Board. Informed written individual or parental consent was obtained for each participant.

### NS1 sequencing.

RNA was obtained from 300 μl of patient nasal washes/swabs using the QIAamp viral RNA extraction kit (Qiagen) according to the manufacturer's instructions. RT reactions were performed during 2 h at 37°C using the high-capacity cDNA reverse transcription kit (Applied Biosystems) and the primers NS1-NCR-5′-VS (5′-AGCAAAGCAGGAGTAAAGATGAATCC-3′, complementary to the 5′ NCR and the first 8 nt of influenza A/Victoria/361/2011 NS1 gene [GenBank accession no. KJ942684.1]) and NEP-3′-RS (5′-CTGTTCCACTTCAAACAGCAGTTGTAATG-3′, complementary to influenza A/Victoria/361/2011 NS1 ORF). The cDNAs were amplified by PCR using Pfx polymerase (Life Technologies) and the same VS and RS primers. Amplified PCR products were used for Sanger sequencing (Genewiz).

### qRT-PCR of PBMCs.

PBMCs from subjects (acute visit, 2 to 3 days after infection) were thawed and rested overnight (10^6^ cells/M24 well) in RPMI medium containing 10% fetal bovine serum and 100 U/ml penicillin, 0.1 mg/ml streptomycin, and 50 μg/ml gentamicin. The following day, PBMCs were treated with 2,000 U/ml of IFN-α or infected (MOI, 1) with r85. At 21 h after treatment or infection, PBMCs were collected and total RNA was extracted using an RNeasy minikit (Qiagen). RT reactions were performed at 37°C for 2 h using the high-capacity cDNA transcription kit (Applied Biosystems) and random hexamer oligonucleotides to generate cDNAs. qPCRs were performed using TaqMan gene expression assays (Applied Biosystems) specific for human IFN-β, IFIT2, and interleukin-29 (IL-29) (IFN-λ3) and glyceraldehyde-3-phosphate dehydrogenase (GAPDH) genes (Hs01077958_s1, Hs01922738_s, Hs00601677_g1, and Hs02758991_g1, respectively) or a TaqMan assay specific for the influenza virus M gene (NR-15592; BeiResources). Quantification was achieved using the 2^−ΔΔ*CT*^ method (33).

## RESULTS

### Variability in innate immune responses among IAV H3N2 virus clinical isolates.

**FIG 1:**
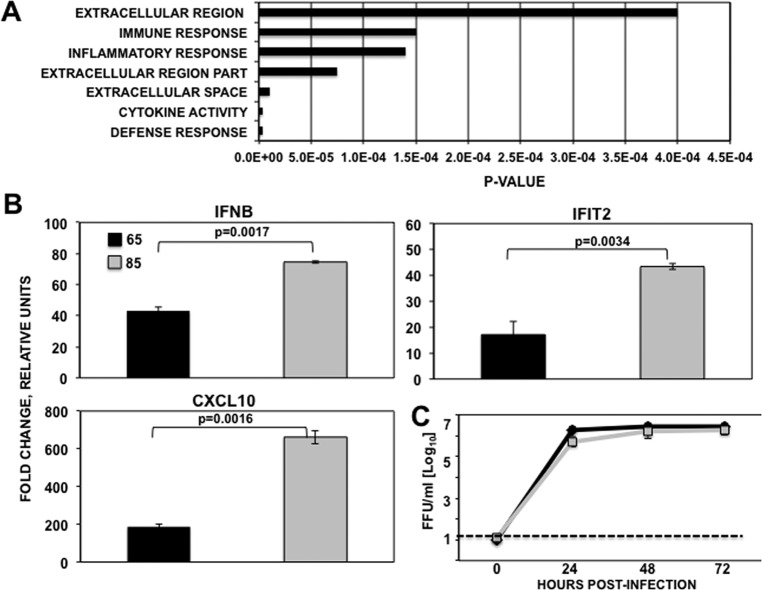
Differential innate immune responses after infection with two different circulating IAV H3N2 viruses. (A) Genes differentially expressed in MDCK cells infected (MOI, 0.01) with virus 65 compared to virus 85-infected MDCK cells were classified according to their main biological functions using the DAVID software. *P* values of the most significant groups are shown on the *x* axis. (B) Canine MDCK cells were mock infected or infected (MOI, 0.01) with IAV A H3N2 65 or 85. RNAs were extracted at 24 hpi, and the expression of cellular genes IFN-β, IFIT2, and CXCL10 was evaluated by qRT-PCR. In each case, the expression levels of mRNAs for representative innate immune response genes were compared to the levels in mock-infected cells. Bars represent standard deviations of the means from triplicates. *P* values determined using Student's *t* test are indicated. (C) MDCK cells were infected (MOI, 0.01) in duplicate with IAV H3N2 65 or 85. Virus titers in supernatants of infected cells at different times pi were determined by immunofocus assay (FFU/ml). The experiments were repeated 3 times, in duplicate, with similar results. Error bars represent the standard deviations for the duplicates in one representative experiment. The dotted line indicates the limit of detection.

We used a collection of IAV H3N2 viruses (see Table 4) isolated from patients in Rochester (NY) (32) during the 2012-2013 season to analyze differences in innate immune responses induced after virus infection. To that end, MDCK cells were infected with the viruses from patients 55, 65, 85, 90, and 91 (MOI, 0.01) isolated from MDCK cells (32). At 16 h postinfection (hpi), total RNAs were purified and analyzed by deep sequencing to compare the transcriptomes of infected cells and to obtain the virus gene sequences. Only the genes showing a fold change of at least 1.5 and a *P* value of <0.05 were considered statistically significant. Whereas gene expression in 85 virus-infected cells was different, the transcriptomes of cells infected with viruses 55, 65, 90, and 91 were very similar (data not shown). For this reason, and to simplify the results, only the transcriptome from the 65 virus-infected cells will be shown and compared to gene expression in mock- and 85 virus-infected cells. A total of 234 and 637 genes were differentially expressed in cells infected with 65 and 85 viruses, respectively, compared to mock-infected cells (see Tables S1 and S2 in the supplemental material), and 79 genes were differentially expressed in cells infected with 65 and 85 viruses (see Table S3). The genes differentially expressed in cells infected with the two viruses were clustered according to their main biological function using DAVID software (36). The most statistically significant clusters were associated with defense response, cytokine activity, extracellular space, extracellular region, inflammatory response, and immune response (Fig. 1A), with IAV 85 inducing an increased innate immune response compared to virus 65. Interestingly, genes encoding IFN-β, type III IFNs (IFN-λ1 and IFN-λ3), many ISGs (RIG-1, ISG56/IFIT1, ISG54/IFIT2, IFIT3, interferon-induced 1 [IFI1], IFI6, guanylate-binding protein 1 [GBP1], GBP5, and GBP6), and inflammatory factors (tumor necrosis factor [TNF], IL-6, IL-8, CXCL10, CCL4, CCL5, IRF1, and BATF2) were differentially upregulated in cells infected with virus 85 compared to virus 65 (Table 3).

**TABLE 3:** Innate immune response genes in 65 and 85 virus MDCK-infected cells

Gene ID	Gene product	Values				Fold change (virus 65 vs virus 85)
		65	85	*P*	*q*^*b*^	
ENSCAFG00000001653	IFN-β1	2.54138	12.7543	0	0	5.018651284
ENSCAFG00000011481	IL-29L (IFN-λ1)	3.29086	26.054	0	0	7.917079426
ENSCAFG00000005588	IL-28B (IFN-λ3)	0.715677	5.40374	0	0	7.550529079
ENSCAFG00000001807	DDX58 (RIG-I)	53.5688	98.9914	8.39E−07	0.000134937	1.847930138
ENSCAFG00000009617	IFIT1	23.2887	57.8476	3.31E−14	1.31E−11	2.483934269
ENSCAFG00000009612	IFIT2	6.69669	12.2205	2.67E−07	4.80E−05	1.824856758
ENSCAFG00000031614	IFIT3	27.2511	54.1892	3.90E−09	9.18E−07	1.988514225
ENSCAFG00000012657	IRGM (IFI1)	19.8128	36.6909	1.43E−07	2.69E−05	1.851878584
ENSCAFG00000012046	IFI6	1.58598	5.74363	1.07E−11	3.45E−09	3.621502163
ENSCAFG00000020204	GBP1	1.35786	3.5978	6.75E−07	0.000111732	2.649610416
ENSCAFG00000024540	GBP5	1.84438	4.11102	1.21E−09	3.14E−07	2.228944144
ENSCAFG00000020200	GBP6	0.485786	1.80615	0	0	3.717995167
ENSCAFG00000000517	TNF-α	0	0.228953	0.000492312	0.0318383	—^*a*^
ENSCAFG00000002733	Q95LE4 (IL-6)	0.0324374	0.305077	0.000226062	0.0170348	9.405100285
ENSCAFG00000003029	IL-8	1.45821	3.01756	1.49E−06	0.000227973	2.069359009
ENSCAFG00000008584	CXCL10	96.6783	311.781	0	0	3.224932586
ENSCAFG00000018164	CCL4	0.0471098	0.675799	9.07E−06	0.0011344	14.34518932
ENSCAFG00000018171	CCL5	2.59183	12.71	0	0	4.90387101
ENSCAFG00000000851	IRF1	9.35472	17.4592	8.77E−08	1.71E−05	1.866351959
ENSCAFG00000014017	BATF2	3.80272	6.47318	0.000572323	0.0357576	1.702249968

a —, no accurate fold change can be calculated as the denominator is 0.

b *q* value indicates the false-discovery rate.

To confirm that IAV isolated from patient 85 induced higher levels of innate immune response transcripts than those obtained from the cells infected with the virus isolated from patient 65, MDCK cells were infected at an MOI of 0.01 and the expression of IFN-β, ISG54/IFIT2, and CXCL10 genes was analyzed by qRT-PCR (Fig. 1B). Compared to mock-infected cells, upregulation of IFN-β, ISG54/IFIT2, and CXCL10 transcripts was higher in cells infected with IAV 85 than in cells infected with IAV 65 (Fig. 1B). To rule out the possibility that IAV 85 induced a higher immune response due to higher levels of viral replication, growth kinetics were determined. To that end, MDCK and A549 cells were infected with both virus isolates at the same MOIs (0.01 and 0.5 for MDCK and A549 cells, respectively), and TCS were collected at 24, 48, and 72 hpi (Fig. 1C and data not shown). Viruses isolated from both patients grew to similar titers in MDCK and A549 cells, suggesting that the differences in innate immune responses were not due to differences in virus growth (Fig. 1C and data not shown).

To analyze whether differences in innate immune responses between cells infected with viruses 65 and 85 could be due to viral protein differences, amino acid changes between both viruses were analyzed. Differences in viral genes HA, NA, PA-X, PB1, PB2, and NS1 were detected between both viruses (see Table S4 in the supplemental material). However, influenza virus NS1 is the main viral protein that suppresses host innate immune responses allowing the virus to replicate efficiently (reviewed in reference 7), suggesting that one or more of the 3 mutations found in the NS1 protein is responsible for differential induction of host innate immune responses elicited during infection (Fig. 1).

To rule out the possibility that the differences in innate immune responses induced after infection with viruses 65 and 85 were due to coinfections with different strains of influenza viruses or to coinfections with other pathogens leading to immunosuppression, we looked for other microorganism sequences and for the presence of SNPs in the influenza virus using the deep-sequencing data collected from the 65 and 85 virus-infected cells. We did not find any sequences for any other microorganisms, strongly suggesting that cells were not coinfected with other pathogens. When looking at influenza virus SNPs, we found low-frequency sequences containing changes in NA, HA, PA, and PB2 (see Table S5 in the supplemental material). However, as these genes have not been implicated to date in the antagonism of innate immune responses after influenza virus infection, these data suggested that the differences in innate immune responses are not due to coinfections with other influenza viruses.

### Variability in NS1 protein sequence among IAV H3N2 clinical isolates.

To further assess variability in the NS1 protein of IAV H3N2 viruses circulating within the 2012-2013 season in the Rochester (NY) area, the NS1 protein sequences of 10 additional clinical samples from the same 2012-2013 season were obtained (GenBank accession numbers KU715844, KU715845, KU715848, KU715849, KU715851, KU715853, KU715854, KU715855, KU715856, KU715857, KU715859, and KU715860). Clinical isolates from patients 54FUR0056, 54FUR0058, 54FUR0064, 54FUR0065, 54FUR0068, 54FUR0072, 54FUR0073, 54FUR0090, and 54FUR0091 (referred to here by the last two numbers in their identifiers) encoded the same NS1 sequence. However, IAV infecting patient 55 presented an E26K change, while the virus infecting patient 87 presented E26K and R224K changes. Finally, the virus from patient 85 presented E26K, I64T, and R224K changes (Table 4). These data indicate the presence of genetic variability in the NS1 protein among A/H3N2 IAVs infecting people both in the same area and within the same season.

**TABLE 4:** Amino acid changes in NS1 proteins from influenza A/H3N2 virus circulating during 2012-2013 season

Patient	Amino acid at position:		
	26	64	224
55	K	I	R
56	E	I	R
58	E	I	R
64	E	I	R
65	E	I	R
68	E	I	R
72	E	I	R
73	E	I	R
85	K	T	K
87	K	I	K
90	E	I	R
91	E	I	R
Consensus^*a*^	K	I	R

a The consensus sequence shows the most prevalent amino acid found in A/H3N2 NS1 proteins from 2012-2013 human viruses (IRD).

### Effect of NS1 changes on translation inhibition and host innate immune responses.

**FIG 2:**
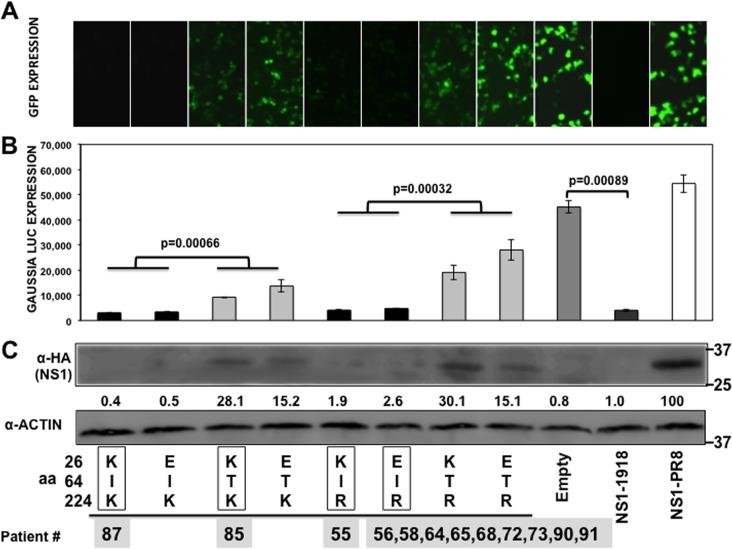
Effect of IAV H3N2 NS1 mutations at positions 26, 64, and 224 on general gene expression. Human 293T cells were transiently cotransfected with pCAGGS plasmids expressing the indicated HA epitope-tagged NS1 proteins, along with GFP- and Gluc pCAGGS-expressing plasmids, using DNA-IN. (A) At 30 hpt, GFP expression was visualized using a fluorescence microscope. (B) At 30 hpt, Gluc expression was analyzed using luminescence. Error bars represent the standard deviations for triplicates. *P* values using Student's *t* test are indicated. (C) IAV NS1 protein and cellular actin protein expression levels were analyzed by Western blotting from cell extracts using antibodies specific to the HA tag (to detect the NS1 protein) and to actin (as a loading control). Western blots were quantified by densitometry using the software ImageJ (v1.46), and the amounts of NS1 protein were normalized to the amounts of actin protein. Protein expression in cells transfected with the plasmid expressing PR8-NS1 was considered to be 100% for comparison to the levels of expression by the other NS1 variants (numbers below the NS1 blot). Molecular mass markers (in kilodaltons) are indicated on the right. The experiments were repeated 3 times with similar results. The squares represent the NS1 variants found in the subjects (Table 4), showing the numbers of patients for each NS1 variant below the squares. aa, amino acid.

Influenza NS1 protein is a multifunctional protein that plays several key roles during the replication cycle of the virus (reviewed in reference 7). One of the main functions of influenza NS1 protein is to counteract the type I IFN response (reviewed in reference 7). Additionally, the NS1 protein of some IAV has been shown to inhibit host protein expression by binding to CPSF30 (16–18) and PABPII (23). To analyze the effect of A/H3N2 NS1 changes in positions 26, 64, and 224 (in the same backbone) on the inhibition of host gene expression, human 293T cells were cotransfected with plasmids expressing GFP and Gluc and with plasmid encoding the different NS1 variants under the control of a polymerase II promoter (26) or with the empty plasmid as a control (Fig. 2). Plasmids expressing NS1 from A/BM/1/18 H1N1 (1918) and A/PuertoRico/8/34 H1N1 (PR8) were used as additional controls since they represent NS1 proteins which do and do not block general gene expression, respectively (17). At 30 hpt, GFP (Fig. 2A) and Gluc (Fig. 2B) expression levels were evaluated using fluorescence microscopy and a LumiCount luminometer, respectively. As previously shown (17), 1918 NS1 protein inhibited protein expression of both reporter genes, whereas PR8 NS1 protein did not. The NS1 proteins encoded by H3N2 IAVs inhibited protein synthesis to different extents (Fig. 2A and B). The levels of Gluc and GFP expression were similar in cells expressing NS1 proteins containing amino acid changes at positions 26 and 224 (Fig. 2A and B), suggesting that these mutations do not significantly affect the ability of the NS1 protein to inhibit cellular gene expression. However, the transfection of plasmids encoding NS1 proteins containing T at amino acid position 64 led to increased levels of reporter gene expression in the cells, indicating that this mutation affected the ability of NS1 to block host gene expression. On the other hand, cells transfected with plasmids encoding the NS1 proteins containing an I at position 64 expressed reduced levels of reporter proteins, similar to that observed with the 1918 NS1 protein. These results were further confirmed when NS1 expression levels were analyzed by Western blotting. As expected, NS1 proteins that efficiently blocked GFP and Gluc expression (I64) were barely detected by Western blotting (Fig. 2C), similar to the situation with the 1918 NS1 protein (17). On the other hand, NS1 proteins containing T64 were easily detected in transfected cell lysates, similar to the situation with PR8 NS1 (17) (Fig. 2C).

**FIG 3:**
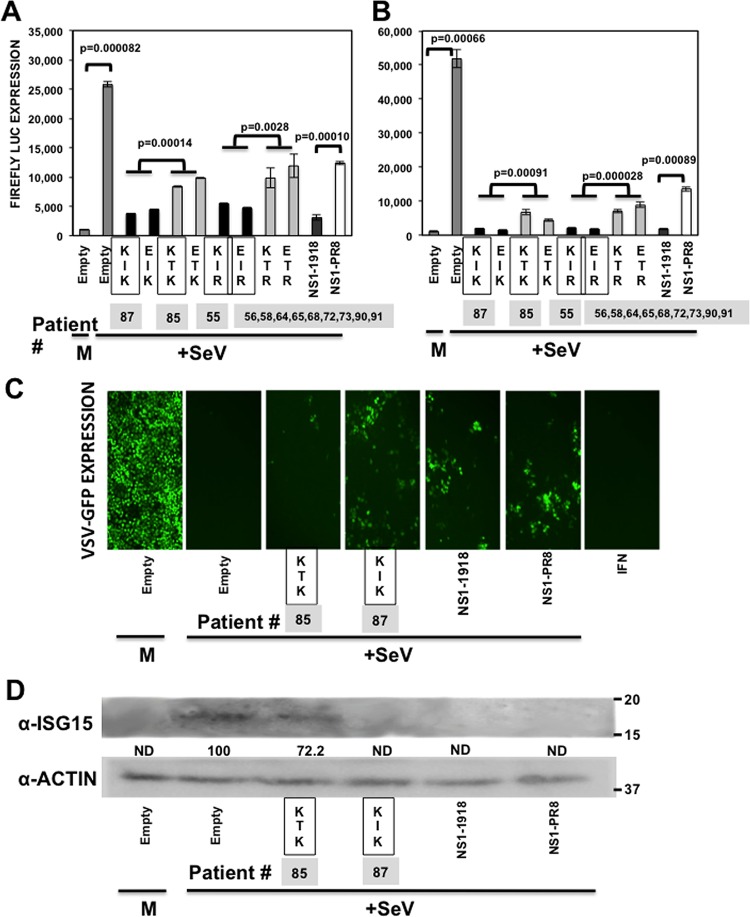
Effect of IAV H3N2 NS1 protein mutations on innate immune responses induced by SeV infection. Human 293T cells were transiently transfected using the calcium phosphate method with the indicated pCAGGS NS1-expressing plasmids, together with plasmids expressing Fluc under the control of an IFN-β (A) or ISRE promoter (B). At 24 hpt, cells were mock infected (M) or infected with SeV (Cantell strain) (+SeV) to induce activation of the promoters, and 16 hpi cell lysates were prepared for reporter gene expression. (A and B) Reporter Firefly expression was measured by luminescence. Data represented show the means and standard deviations of the results determined for triplicate wells. Experiments were repeated 3 times in triplicate wells with similar results. *P* values determined using Student's *t* test are indicated. (C) At 16 h after SeV infection, TCS were collected and, after UV inactivation, were used to treat fresh A549 cells. Alternatively, A549 cells were treated with 2,500 U/ml of universal IFN-α as a control (picture on the right). After 24 h of incubation, cells were infected (MOI, 0.001) with the IFN-sensitive VSV-GFP. At 16 hpi, VSV-GFP-infected cells were observed under a fluorescence microscope. (D) At 16 h after SeV infection, cellular extracts were obtained and the expression of ISG15 and actin were evaluated by Western blotting, using antibodies specific to ISG15 and to actin (as a loading control). Western blots were quantified by densitometry using the software ImageJ (v1.46), and the amounts of ISG15 protein were normalized to the amounts of actin protein (numbers below the ISG15 blot; ND, not detected). Molecular mass markers (in kilodaltons) are indicated on the right. The squares represent the NS1 variants found in the subjects (Table 4), showing the numbers of patients for each NS1 variant below the squares.

To analyze whether the amino acid differences observed in the IAV H3N2 NS1 variants affected their ability to counteract the innate immune responses, two complementary assays were performed. In the first assay, human 293T cells were cotransfected with plasmids expressing the different NS1 proteins under a polymerase II promoter, with plasmids expressing Fluc under the control of the IFN-β (Fig. 3A) or an ISRE (Fig. 3B) promoter and with a plasmid constitutively expressing Rluc. At 24 hpt, cells were left mock infected or were infected with SeV (Cantell strain) to induce activation of both promoters. At 16 hpi, IFN-β and ISRE promoter activation was determined by quantifying levels of Fluc (Fig. 3A and B), the levels of secreted IFN were quantified in a bioassay (Fig. 3C), and the levels of the endogenous IFN-stimulated protein 15 (ISG15) were analyzed by Western blotting (Fig. 3D). As expected, SeV infection induced robust activation of both IFN-β and ISRE promoters in cells transfected with the empty plasmid. As expected, PR8 NS1 inhibited SeV-induced activation of both promoters to lower extents than 1918 NS1 (17). In cells transfected with the different IAV H3N2 NS1 variants, the levels of Fluc were reduced compared to those for empty plasmid-infected cells, consistent with previous results showing that IAV NS1 proteins counteract SeV-induced activation of both promoters (reviewed in reference 7). Notably, NS1 proteins containing T at position 64 inhibited IFN and ISRE promoter activation to lower extents than those containing I64 (Fig. 3A and B), similar to the results obtained with PR8 NS1. To analyze the levels of SeV infection-induced IFN in cells overexpressing the different NS1 variants, TCS were UV inactivated and used to treat A549 cells. As a control, cells were treated with 2,500 U/ml of universal IFN-α. The cells then were infected with the IFN-sensitive VSV-GFP, and the levels of GFP expression were analyzed (Fig. 3C). GFP expression was high in cells treated with TCS from empty plasmid-transfected, mock-infected cells. In contrast, the levels of GFP expression decreased in cells treated with supernatants from SeV-infected, NS1-I64-transfected cells and, to a much lower extent, from SeV-infected NS1-T64-transfected cells (Fig. 3C). This bioassay indicated that the levels of secreted IFN induced after SeV infection were lower in cells transfected with NS1-I64 than in cells transfected with NS1-T64. Remarkably, and confirming the previous results, levels of ISG15 expression were detected by Western blotting in SeV-infected cells which were transfected with empty plasmid, and with the plasmid expressing the T64-NS1, but not in cells transfected with I64-NS1, PR8-NS1, and 1918-NS1 plasmids (Fig. 3D). As a control, the levels of the cellular actin protein were similar in all cases (Fig. 3D). These results indicated that I64T substitution affected the ability of NS1 to inhibit SeV-mediated activation of both promoters, the induction of secreted IFN, and the induction of IFN-stimulated genes, such as ISG15, possibly because of a defect in inhibiting general host expression (Fig. 2). In fact, the levels of constitutive Rluc expression in these experiments were similar to those of Gluc (Fig. 2 and data not shown), corroborating that the mutation I64T decreases the ability of the NS1 protein to inhibit general gene expression.

**FIG 4:**
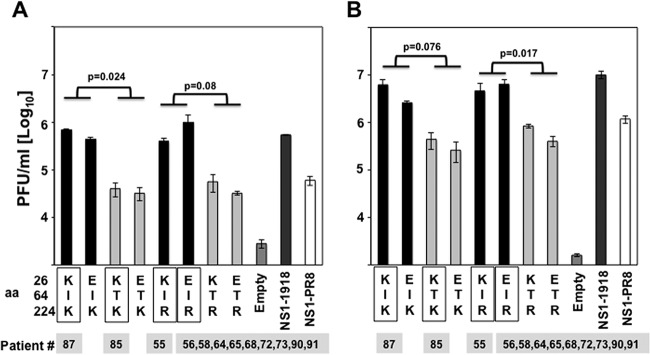
Effect of IAV H3N2 NS1 protein mutations on VSV growth. Human A549 cells were transfected with pCAGGS plasmids expressing the indicated NS1 variants using DNA-IN. At 24 hpt, cells were either transfected with 100 ng of poly(I·C) (A) or treated with 250 U/ml of universal IFN (B) to induce an antiviral cellular state. At 16 h posttreatment, cells were infected (MOI, 0.001) with VSV-GFP, and viral titers in the TCS were determined at 21 hpi. Bars represent the standard deviations from triplicates. Experiments were repeated 3 times in triplicate wells with similar results. *P* values using Student's *t* test are indicated. The squares represent the NS1 variants found in the subjects (Table 4), showing the numbers of patients for each NS1 variant below the squares.

To further analyze whether the amino acid changes found in the NS1 proteins affected their ability to counteract the innate immune responses, human A549 cells were transiently transfected with plasmids expressing the different IAV H3N2 NS1 proteins. Cells transfected with the empty plasmid or with 1918- and PR8 NS1-expressing plasmids were included as controls. At 24 hpt, cells were mock treated or treated with poly(I·C) (Fig. 4A) or IFN-α (Fig. 4B) for 16 h and infected (MOI, 0.001) with VSV-GFP. At 21 hpi, cell culture supernatants were collected and VSV-GFP titers were quantified. Cells transfected with empty plasmid and treated with either poly(I·C) or IFN-α showed reduced titers of VSV-GFP because of the sensitivity of VSV to the antiviral state induced by type I IFN production and signaling. As expected, VSV-GFP titers were ∼10-fold (1 log) higher in cells transfected with 1918 NS1 than in cells transfected with PR8 NS1 in both poly(I·C)-treated (Fig. 4A) and IFN-α-treated (Fig. 4B) cells, consistent with the ability of 1918 NS1 to inhibit host protein expression (Fig. 2). VSV-GFP replication was rescued in cells transfected with the IAV H3N2 NS1 variants and either treated with poly(I·C) (Fig. 4A) or IFN-α (Fig. 4B), consistent with the ability of NS1 to counteract the type I IFN response (reviewed in reference 7). However, there were qualitative differences among the different A/H3N2 NS1 proteins. VSV-GFP titers were also ∼10-fold (1 log) lower in cells expressing NS1 proteins containing the residue T64 than in cells expressing the NS1 proteins containing amino acid I at position 64. Notably, NS1 protein mutations E26K and R224K had no significant effects on VSV-GFP growth. These data showed that the mutation I64T diminished the capability of NS1 to antagonize cellular antiviral responses, whereas the mutations at positions 26 and 224 did not show a significant effect.

**FIG 5:**
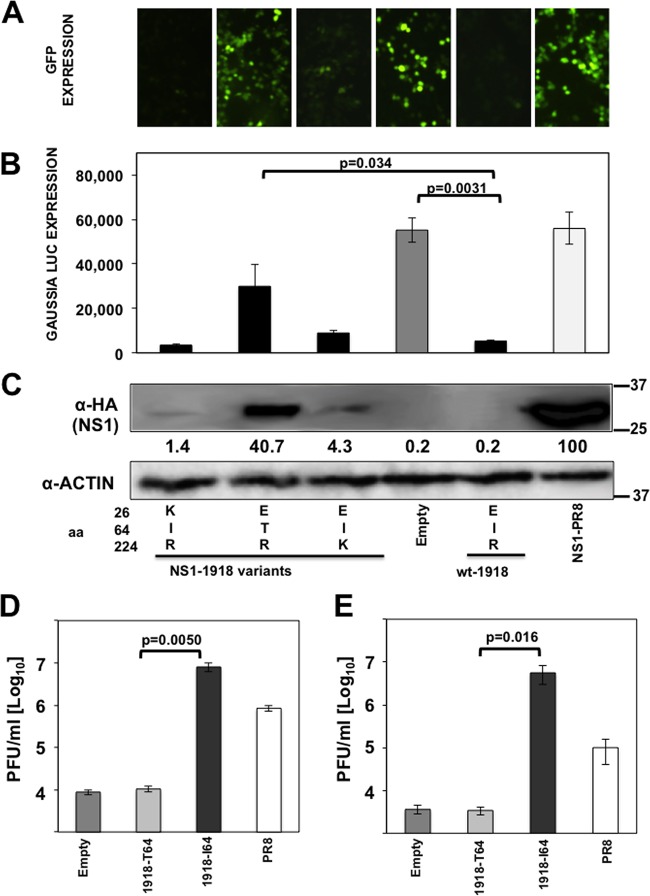
Mutation I64T in IAV 1918 H1N1 NS1 affects its ability to inhibit general gene expression and innate immune responses. (A, B, and C) Human 293T cells were transiently cotransfected with pCAGGS plasmids expressing the different NS1 variants indicated and the WT 1918-NS1 (E26, I64, and R224), along with pCAGGS plasmids expressing the reporter proteins GFP and Gluc. (A) At 30 hpt, GFP expression was visualized using a fluorescence microscope. (B) Gluc expression was analyzed at 30 hpt using luminescence. Error bars represent the standard deviations for triplicates. *P* values using Student's *t* test are indicated. (C) In addition, NS1 and actin expression levels were analyzed by Western blotting from cell extracts using antibodies specific to the HA tag (to detect the NS1 protein) and to actin as a loading control. Western blots were quantified by densitometry using the software ImageJ (v1.46), and the amounts of NS1 protein were normalized to the amounts of actin protein. Protein expression in cells transfected with the PR8-NS1-expressing plasmid was considered to be 100% for comparison with the level of expression by the other NS1 variants (numbers below the NS1 blot). Molecular mass markers (in kilodaltons) are indicated on the right. Three different experiments were performed, with similar results. (D and E) Human A549 cells were transfected with pCAGGS plasmids expressing the 1918-NS1-I64, 1918-NS-T64, or PR8-NS1 protein using DNA-IN. At 24 hpt, cells were either transfected with 100 ng of poly(I·C) (D) or treated with 250 U/ml of universal IFN-α (E) to induce an antiviral cellular state. At 16 h posttreatment, cells were infected (MOI, 0.001) with VSV-GFP, and viral titers in the TCS were determined at 21 hpi. Bars represent the standard deviations from triplicates. Experiments were repeated 3 times in triplicate wells with similar results. *P* values using Student's *t* test are indicated.

To further evaluate the effect of mutations E26K, I64T, and R224K on the ability of NS1 to inhibit host innate immune responses and gene expression, these mutations were introduced in the 1918 NS1 background (Fig. 5). To analyze the effect of these amino acid changes on the ability of 1918 NS1 to inhibit host protein expression, human 293T cells were cotransfected with plasmids expressing GFP and Gluc under the control of the polymerase II promoter and with plasmids encoding the different 1918 NS1 variants, or with the empty plasmid as a control. I64T substitution in 1918 NS1 significantly affected the ability of 1918 NS1 to inhibit host gene expression as evaluated by GFP (Fig. 5A) and Gluc (Fig. 5B) expression. However, substitutions E26K and R224K did not have a significant effect on the ability of 1918 NS1 to inhibit host protein expression (Fig. 5A and B). These results were further confirmed when we evaluated NS1 protein expression levels in cell lysates by Western blotting (Fig. 5C). As expected, 1918 NS1 variants that efficiently blocked GFP and Gluc expression levels (E26K and R224K) were barely detected by Western blotting, similar to WT 1918 NS1 (Fig. 5C). On the other hand, 1918 NS1 I64T was detected by Western blotting, as expected, based on the results obtained with GFP and Gluc reporter assays. Altogether, these data indicate that substitution I64T also affects the ability of 1918 NS1 to inhibit host protein expression and demonstrate the key role of this amino acid residue located in the dsRNA-binding domain of IAV NS1 in regulating general gene expression.

To further analyze the effect of NS1 I64T mutation on the antagonism of innate immune responses, human A549 cells were transiently transfected with plasmids expressing 1918-NS1-I64 (WT protein), 1918-NS1-T64, and PR8 NS1. At 24 hpt, cells were mock treated or treated with poly(I·C) (Fig. 5D) or IFN-α (Fig. 5E) for 16 h and infected (MOI, 0.001) with VSV-GFP. At 21 hpi, cell culture supernatants were collected and VSV-GFP titers were quantified. Cells transfected with empty plasmid and treated with either poly(I·C) or IFN-α showed reduced titers of VSV-GFP because of the sensitivity of VSV to the antiviral state induced by type I IFN production [poly(I·C)-treated cells; Fig. 5D] and signaling (IFN-α-treated cells; Fig. 5E). Interestingly, VSV-GFP titers were ∼1,000-fold (3 logs) higher in cells transfected with 1918-NS1-I64 than in cells transfected with the 1918-NS1-T64 or the empty plasmid-transfected control cells (Fig. 5D and E). These data further confirmed that the mutation I64T diminished the capability of NS1 to antagonize cellular antiviral responses.

### Effect of I64T mutation on the binding of NS1 to CPSF30.

**FIG 6:**
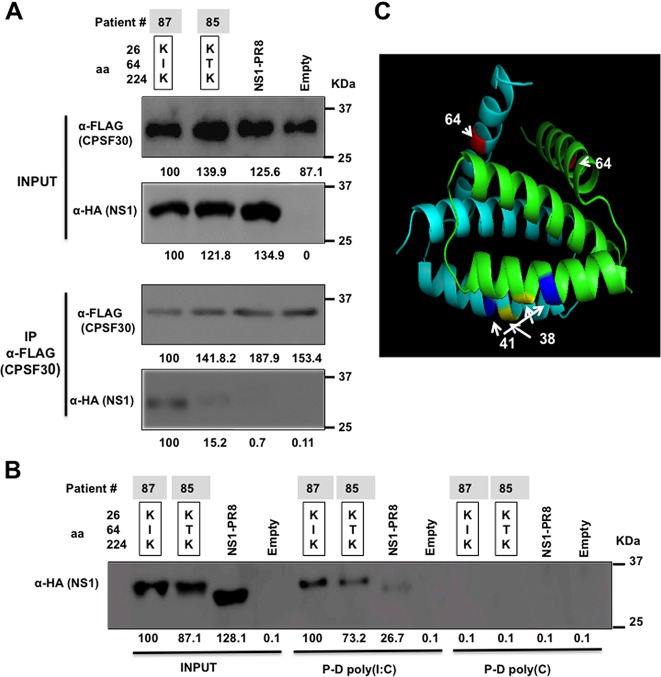
IAV H3N2 NS1 protein I64T mutation decreases the binding to CPSF30 without significantly affecting the binding to dsRNA. (A) FLAG-tagged CPSF30 was expressed in 293T cells, mixed with *in vitro*-synthesized HA-tagged NS1 variants, and immunoprecipitated using an anti-FLAG resin. Following SDS-PAGE, input and immunoprecipitated (IP) proteins were detected by Western blotting using antibodies specific for the HA tag (NS1 proteins) or the FLAG tag (CPSF30 protein). Western blots were quantified by densitometry using the software ImageJ (v1.46), and the amounts of NS1 proteins coimmunoprecipitated with CPSF30 were normalized to the amounts of CPSF30 proteins immunoprecipitated. The amounts of CPSF30 and NS1 proteins present in the input and in the coimmunoprecipitation using I64-NS1 protein (first lane) were considered 100% for comparison (numbers below each blot). (B) Poly(C)-conjugated (control) or poly(I·C)-conjugated beads were incubated with the indicated *in vitro*-synthesized HA-tagged IAV H3N2 NS1 variants. Pulled-down NS1 proteins (P-D) were detected by Western blotting using an antibody specific for the HA epitope tag. Western blots were quantified by densitometry using the software ImageJ (v1.46), and the amounts of NS1 proteins pulled down with poly(I·C) or poly(C) were normalized to the amounts of NS1 proteins detected in the inputs before the pulldown. The amount of I64-NS1 protein in the input and after the pulldown was considered 100% for comparison with the amounts of the other NS1 variants (numbers below the NS1 blot). Three different experiments were performed with very similar results. The squares represent the combinations found in the subjects (Table 4), showing the number of the patient (85 or 87). Molecular mass markers (in kilodaltons) are indicated on the right. (C) Tridimensional protein structure of NS1-dsRNA binding domain. The NS1-dsRNA binding domain structure is based on the NS1 structure of influenza A virus A/crow/Kyoto/T1/2004(H5N1) (PDB entry 2Z0A) and was created using Swiss-Model (52). The structure was colored using the MacPyMOL molecular graphics system (pymol.org). Each monomer of the NS1-dsRNA binding domain is represented in light blue and green, respectively. The positions 38, 41, and 64 of NS1 protein are indicated and represented in yellow, blue, and red, respectively.

The NS1 proteins of some IAV strains interact with CPSF30, inhibiting host protein expression (16–19). To assess whether I64T mutation plays a role in the binding of NS1 to CPSF30, cell extracts from human 293T cells transfected with a plasmid encoding FLAG-tagged CPSF30 (17) were incubated with *in vitro*-transcribed and -translated NS1 proteins from patients 85 and 87 (differing only at I64T position), or with PR8 NS1 as control, and with agarose beads conjugated with an anti-FLAG antibody (Fig. 6). As expected, PR8 NS1 did not coimmunoprecipitate with CPSF30 according to previous results showing that PR8 NS1 does not interact with CPSF30 (17). Interestingly, NS1 from patient 87 (I64) coimmunoprecipitated with CPSF30, while the amount of NS1 T64 (patient 85) was significantly reduced. Importantly, the amounts of both IAV H3N2 NS1 and CPSF30 proteins used for the coimmunoprecipitation and the amounts of coimmunoprecipitated CPSF30 proteins were similar in all cases (Fig. 6A). Altogether, these results indicate that I64T substitution decreases IAV NS1 binding to CPSF30 and provides a mechanism for the impairment of T64-NS1-mediated inhibition of general gene expression.

### Effect of I64T mutation on the binding of NS1 to dsRNA.

To analyze whether I64T substitution could also impair the binding of NS1 to dsRNA, agarose beads conjugated to poly(I·C) were incubated with the *in vitro*-transcribed and -translated NS1 from patients 85 and 87 (differing only in the I64T position) or with PR8 NS1 as a control. We also included agarose beads conjugated to poly(C) to demonstrate the specific interaction of NS1 with poly(I·C). As expected, all NS1 proteins were pulled down using poly(I·C) agarose beads but not with the poly(C) agarose beads, suggesting that the pulldown was specific (Fig. 6B). No significant differences were observed between the precipitated amounts of I64-NS1 and T64-NS1 proteins (Fig. 6B), suggesting that mutation I64T does not significantly impair the binding of NS1 to dsRNA. According to these data, the tridimensional structure of the NS1-dsRNA binding domain showed that each NS1-dsRNA binding domain monomer forms three antiparallel α-helices, with residue 64 located in α-helix 3, whereas residues important for dsRNA binding, such as 38 and 41 (37), were located in α-helix 2 (Fig. 6C).

### Effect of I64T mutation on virulence, virus growth, and IFN-β induction *in vivo*.

**FIG 7:**
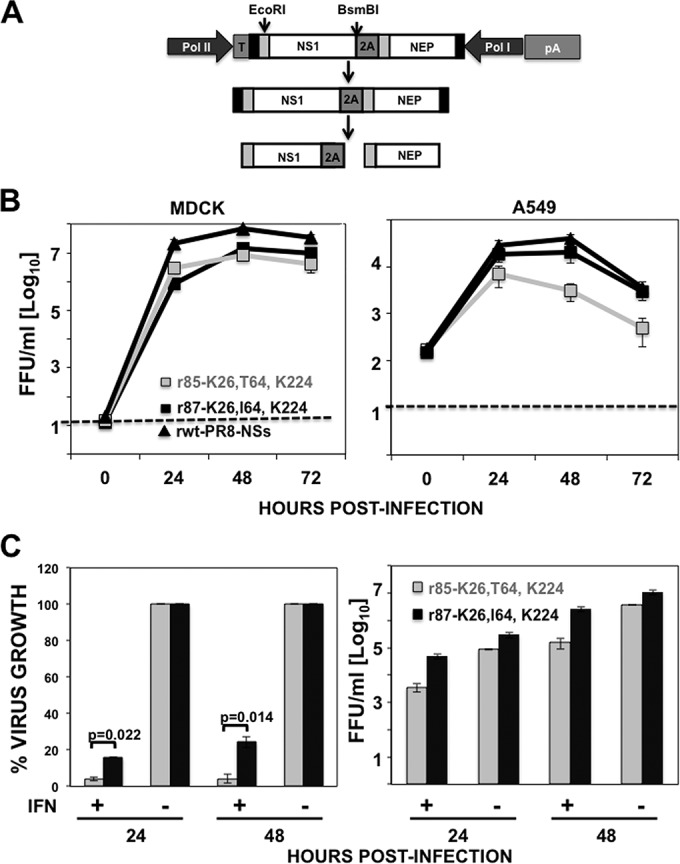
Recombinant virus growth kinetics *in vitro*. (A) Scheme of the pDZ plasmid encoding the NS split segment (27) used to rescue the viruses r65 and r85 (top lane) as well as the NS segment encoding the NS1 and NEP proteins (second lane) being processed by the porcine teschovirus-1 (PTV-1) 2A autoproteolytic cleavage site (third lane). Viral 3′ and 5′ noncoding regions are indicated with black boxes at the end of the viral segment. Viral products from the NS (NS1 and NEP) segment are indicated with white boxes. The region before the splicing donor in the viral segments is indicated with light gray boxes. The sequence of the PTV-1 2A autoproteolytic cleavage site (2A) is indicated with dark gray boxes. The sequences encoding the polymerase (Pol) I and II promoters, the Pol I promoter terminator (T), and the poly(A) tail (pA) are indicated. The position of restriction sites EcoRI and BsmBI, used to clone the NS1 sequences from patients 65 and 85, is shown. (B) Canine MDCK (left) and human A549 (right) cells were infected in duplicate with recombinant PR8 viruses expressing either 65 and 85 H3N2 NS1 proteins or the PR8 NS1 protein (wt-PR8-NSs) at an MOI of 0.001. Virus titers in infected cell supernatants were determined at different times postinfection by immunofocus assay. (C) MDCK cells were infected (MOI, 0.001) with viruses r65 and r85, and just after the infection, cells were left untreated or were treated with 2,000 U of IFN-α/well. Supernatants were collected at 24 and 48 hpi, and virus titers were determined by immunofocus assay. (Left) The percentage of growth in MDCK cells treated with IFN-α (+) was normalized to the growth in the nontreated cells (−) at 24 and 48 hpi (considered 100% growth). (Right) Virus titers (in FFU/ml) in nontreated (−) and IFN-treated (+) cells are shown. The experiments were repeated 3 times in duplicate wells. The dotted line in panel B indicates the limit of detection.

NS1 has been previously shown to play a significant role in IAV virulence *in vivo* (8, 9, 38). Likewise, mutations affecting the ability of NS1 to counteract the host innate immune response have been shown to reduce the virulence of IAV *in vivo* (39, 40). To analyze whether I64T mutation leads to virus attenuation, recombinant viruses encoding the NS1 proteins from patients 85 and 87 (differing only in the amino acid at position 64) were generated (r85 and r87, respectively) (Fig. 7). To this end, a previously described PR8 virus encoding a split NS segment (NSs) was used (27). In this PR8 NSs virus, the NS1 and NEP ORFs are separated by the PTV 2A autoproteolytic cleavage site (Fig. 7A) (27). This allowed the introduction of mutations in NS1 without affecting the amino acid sequence of NEP (27). Both viruses were rescued using previously described plasmid-based reverse genetic approaches (29), and virus growth of these viruses and of a virus encoding the NS1 from PR8 encoding the NS split segment (rwt-PR8-NSs) (27) was determined in canine (MDCK) and human (A549) cells infected at low MOI (0.001) (Fig. 7B). In MDCK cells, both recombinant PR8 viruses showed similar growth kinetics and reached viral titers of ∼10^7^ FFU/ml at the peak of infection (48 hpi). However, in A549 cells, r85 was attenuated compared to r87 at all tested times postinfection. Moreover, r85 viral titers were approximately 5- to 10-fold lower than those for r87 (Fig. 7B). The titers reached by virus rwt-PR8-NSs were around 10-fold higher than titers for virus r87 in MDCK cells and similar to that of virus r87 in A549 cells (Fig. 7B). Altogether, these results indicated that r85 was significantly affected in replication when tested in cells related to those targeted during natural human infection (i.e., human airway epithelial [A549] cells) (41).

To demonstrate that the differences in growth observed in A549 cells were due to differences in the ability to mount an effective antiviral type I IFN response and the ability of r87 (I64) to better overcome the antiviral state induced by type I IFN compared to r85 (T64), MDCK cells were infected (MOI, 0.001) with r85 and r87 viruses. After viral adsorption, cells were left untreated (control) or were treated with 2,000 U/ml of universal type I IFN-α, and virus titers were determined at 24 and 48 hpi. After IFN-α treatment, compared to type I IFN-untreated cells, r85 viral titers were decreased more than 20-fold (∼ 95%) at both 24 and 48 hpi. In contrast, r87 viral titers were only decreased by 6- and 4-fold (∼85 and 75%) at 24 and 48 hpi, respectively (Fig. 7C). These data indicated that r85 virus containing T64-NS1 protein is more sensitive to the antiviral effect of exogenous type I IFN than r87 (I64) (Fig. 7C).

**FIG 8:**
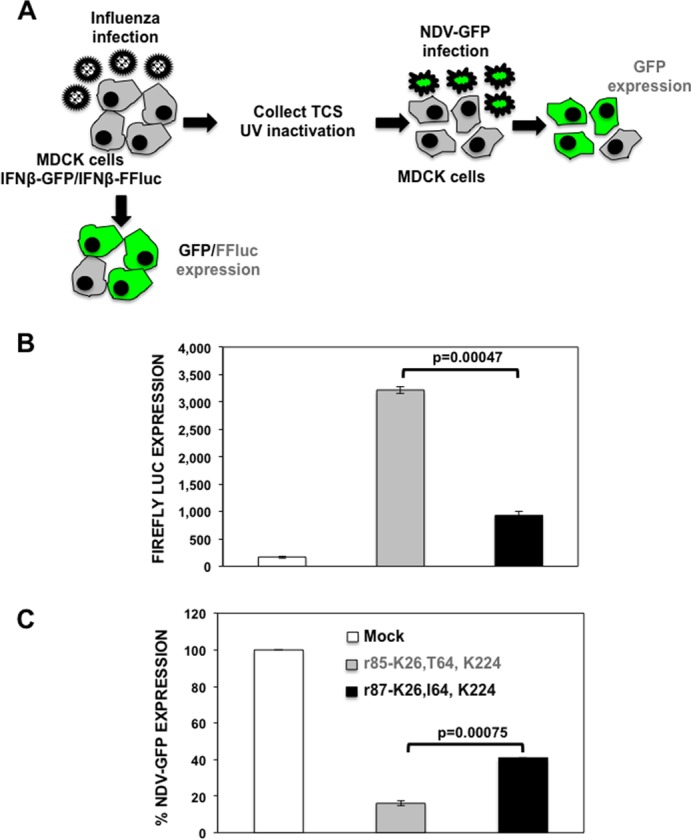
Induction of IFN by r85 and r87 viruses. (A) Schematic representation of the IFN-β induction bioassays. MDCK cells constitutively expressing GFP and Fluc reporter genes under the control of the IFN-β promoter (MDCK IFN-β GFP-CAT/IFN-β Fluc) were infected (MOI, 4) with r85 and r87. (B) At 12 hpi, the activation of the IFN-β promoter was determined by assessing Fluc activity using a microplate reader. (C) TCS of previously infected MDCK cells were collected and, after UV inactivation, were used to treat fresh MDCK cells. After 24 h of incubation, cells were infected (MOI, 5) with the IFN-sensitive NDV-GFP. At 14 hpi, NDV-GFP-infected cells were analyzed by visualizing GFP expression. Bars represent the standard deviations from triplicates. Experiments were repeated 3 times in triplicate wells with similar results. *P* values determined using Student's *t* test are indicated.

To test whether I64T mutation diminishes the NS1 protein's ability to counteract host innate immune responses in the context of virus infection, MDCK cells constitutively expressing GFP and Fluc reporter genes under the control of the IFN-β promoter (MDCK IFN-β GFP-CAT/IFN-β Fluc) (30) were mock infected or infected (MOI, 4) with either r85 or r87 virus (Fig. 8A). At 12 hpi, the activation of the IFN-β promoter was determined by evaluating GFP expression (data not shown) and quantifying the Fluc activities (Fig. 8B). Importantly, comparable levels of infection in MDCK IFN-β GFP-CAT/IFN-β Fluc cells were verified by immunofluorescence using an anti-NP antibody (data not shown). The levels of GFP signal in these cells were a reflection of the amounts of IFN produced in cells infected with the NS1 mutant viruses. The GFP signal was not detected in mock-infected cells, as expected (data not shown). In r87 virus-infected cells, very low levels of GFP expression were detected, consistent with the data showing that the r87 virus counteracts the IFN response induced by the host. However, in cells infected with the r85 virus, higher levels of GFP expression were expressed (data not shown). Furthermore, this observation was quantified through an Firefly-luciferase (Fluc) assay (Fig. 8B). Data correlated with the GFP levels observed previously, indicating that virus with the mutation I64T presented defects in counteracting host defenses.

NDV infection is highly affected by previous antiviral states induced in the cells (42). Therefore, as a second approach to analyze the IFN responses induced by the NS1 mutant viruses, inhibition of viral infection of a recombinant NDV expressing GFP (NDV-GFP) was evaluated in MDCK cells treated with the UV-inactivated supernatants collected in the first bioassay (Fig. 8A). Inhibition of NDV-GFP was evaluated by GFP expression and was dependent on the amounts of IFN present in the supernatants of the cells infected with the different viruses. In cells pretreated with supernatants from mock-infected and, to a lesser extent, r87 virus-infected cells, NDV-GFP replicated efficiently, as evidenced by high levels of GFP expression (Fig. 8C). However, NDV-GFP replication was compromised in cells treated with the supernatant from r85-infected cells. Altogether, these data confirmed that infection with r85 virus induced an increased IFN response compared to infection with the r87 virus.

**FIG 9:**
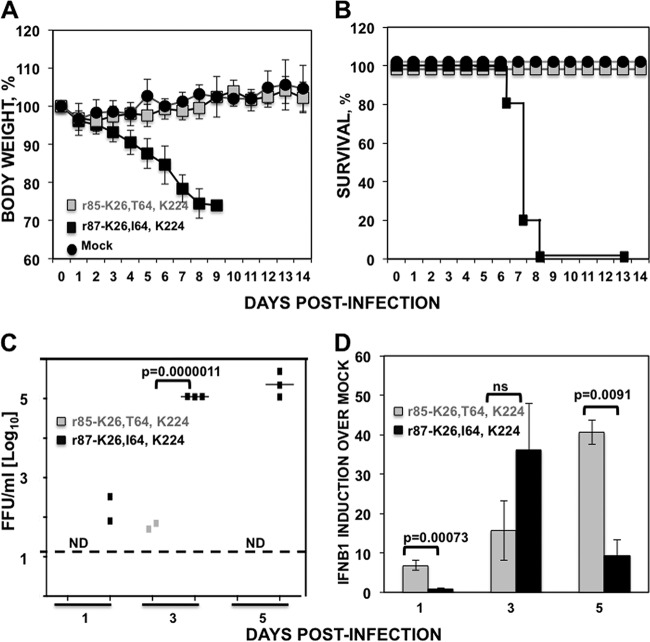
Virulence, growth, and IFN-β induction of recombinant PR8 viruses containing T64 or I64 mutations in NS1. (A and B) Groups (*n* = 5) of 7- to 8-week-old C57BL/6 female mice were infected (50 FFU/mouse) with recombinant PR8 viruses containing a split NS segment encoding NS1 from 65 or 85 H3N2 IAVs. Weight loss (A) and survival (B) were evaluated daily for 2 weeks. (C and D) Groups (*n* = 3) of 7- to 8-week-old C57BL/6 female mice were infected (50 FFU/mouse) with recombinant PR8 viruses containing a split NS segment encoding the NS1 from 65 or 85 H3N2 IAVs. (C) Mice were sacrificed at 1, 3, and 5 dpi, and lungs were harvested, homogenized, and used to quantify viral titers by immunofocus assay (FFU/ml). The dotted black line indicates the limit of detection (20 FFU/ml). ND, not detected. *P* values determined using Student's *t* test are indicated. (D) Mice were sacrificed at 1, 3, and 5 dpi, and total RNA was extracted to quantify levels of mRNA expression by qRT-PCR. *P* values determined using Student's *t* test are indicated. ns, not significant.

We next evaluated the effect of NS1 protein I64T mutation in virus growth and virulence *in vivo*. To that end, mice (*n* = 5) were infected intranasally (i.n.) with 50 FFU of r85 or r87 or were mock infected. Morbidity (body weight loss) and mortality (percent survival) were monitored for 14 days (Fig. 9A and B, respectively). All mice infected with r87 rapidly lost weight (Fig. 9A) and succumbed to viral infection by day 9 (Fig. 9B). In contrast, mice infected with r85 did not lose weight and all survived the infection, similar to the mock-infected control group (Fig. 9A and B). These results suggested that NS1 I64T mutation generates a virus with an attenuated phenotype *in vivo* that correlates with the inability of NS1 to inhibit host gene expression and therefore to counteract the antiviral state induced by type I IFN responses.

To analyze whether the observed attenuation correlated with virus titers in the lungs, groups of mice (*n* = 3) were infected (i.n.) with 50 FFU of r85 and r87, and virus titers in lungs extracted at 1, 3, and 5 days postinfection (dpi) were evaluated (Fig. 9C). At 1 dpi, low titers (less than 10^3^ FFU/ml) were detected in 2 out of 3 mice infected with virus r87, and no infectious virus was detected in mice infected with virus r85. High viral titers (>10^5^ FFU/ml) were detected in the lungs of mice infected with IAV r87 at 3 and 5 dpi, while low titers (<10^2^ FFU/ml) were detected in the lungs of 2 out of 3 mice at 3 dpi and no virus was detected at 5 dpi in mice infected with IAV r85 (Fig. 9C). These data indicated that r85 is strongly impaired in growth *in vivo*, correlating with the absence of morbidity and mortality observed in the infected mice.

To determine whether incorporating the mutation I64T in the NS1 protein induces higher IFN-β responses *in vivo*, the levels of IFN-β mRNA were evaluated in lungs from mice infected with virus r85 and r87 at 1, 3, and 5 dpi (Fig. 9D). Higher IFN-β levels were observed at 1 and 5 dpi in mice infected with r85 compared to mice infected with r87 (Fig. 9D), consistent with previous results showing that the mutation I64T impairs the function of NS1 to counteract host defenses. At 3 dpi, no significant differences in IFN-β induction were observed for either virus. This is probably because r87 virus is actively replicating at 3 dpi and therefore inducing IFN-β expression, whereas replication of r85 is highly attenuated (Fig. 9C). On day 5, IFN-β levels in the r85-infected mice were significantly higher than those in mice infected with r87, demonstrating r85's inability to block IFN induction. These results, together with the previous observation that r85 is more sensitive to type I IFN responses (Fig. 7C), most probably accounts for the attenuated phenotype of r85 *in vivo*.

### Mutation I64T in the NS1 protein is found very rarely in circulating human, swine, and avian influenza A viruses.

To analyze the frequency of I64T mutation in IAV infecting subjects worldwide, the sequences from H3N2 NS1 proteins from human viruses circulating since 1968 (from the Influenza Research Database [IRD]) were analyzed. Interestingly, from 6,980 sequences, most of them (6,976) contain I64 (99.9%), 2 contain T64 (0.03%), 1 contains L64 (0.015%), and 1 contains V64 (0.015%) (see Table S6 in the supplemental material). These data suggest, as shown in the manuscript, that amino acid I64 is relevant for the NS1 function(s), as other amino acids at this position are present only in very minor frequencies.

To analyze whether the low frequency of the I64T mutation also applies to other influenza subtypes, such as human H1N1, human 2009 pandemic H1N1, human H5, human H7, avian H1N1, avian H3N2, swine H1N1, swine 2009 pandemic H1N1, and swine H3N2, NS1 protein sequences from the Influenza Research Database were further analyzed (see Table S6 in the supplemental material). As observed for human H3N2 viruses, the frequencies of amino acids other than I64 were very low (<0.12%) in all analyzed sequences.

### Reduced IFN response in the subject infected with the virus containing NS1-T64.

**FIG 10:**
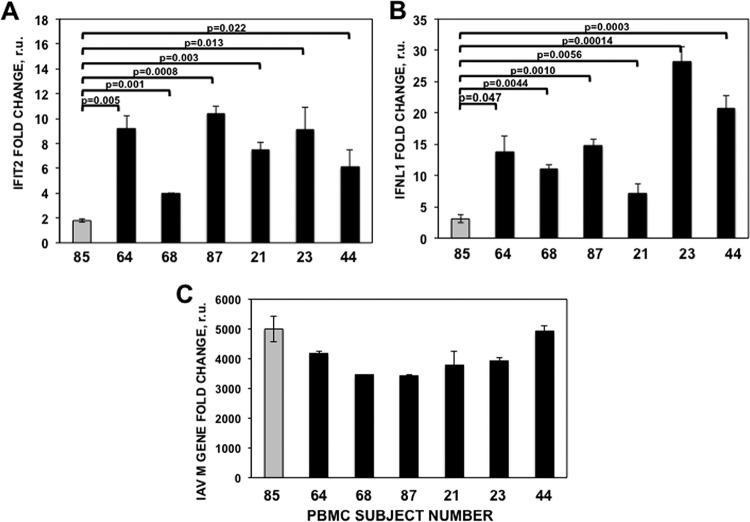
Induction of IFN responses on PBMCs from infected subjects. PBMCs from subject 85 (infected with the virus encoding T64-NS1; in gray) and from subjects 64, 68, 87, 21, 23, and 44 (infected with viruses encoding I64-NS1; in black) were either treated with 2,000 U/ml of IFN-α (A) or infected (MOI, 1) with virus r85 (B). The levels of mRNA expression of cellular IFIT2 (A), IFN-λ1 (B), or gene M mRNA in the infected PBMCs (C) were analyzed by qRT-PCR. Bars represent standard deviations of the means from triplicates. *P* values determined using Student's *t* test are indicated. The experiments were repeated twice independently, using technical triplicates, with similar results. r.u., relative units.

We have shown that the virus containing NS1-T64 is impaired in antagonizing innate antiviral responses and is highly attenuated, at least in mice. Given these results and the observation that H3N2 IAVs with an NS1-T64 mutation are very rare in the human population, it was surprising that a virus with this mutation was found in a human subject reporting flu symptoms (fever, rhinitis, sore throat, cough, chills, and myalgia). To analyze whether subject 85, infected with the virus containing the T64-NS1 protein, presented a defect in IFN responses, PBMCs from this subject and subjects 87, 64, and 68 (collected during the 2012-2013 season), as well as 21, 23, and 44 (collected during the 2010-2011 season) as controls, were treated with IFN-α (Fig. 10A) or infected with virus r85 (containing T64-NS1 protein) (Fig. 10B). The levels of IFIT2 induction after IFN-α treatment (Fig. 10A) and of IFN-β and IFN-λ1 after r85 virus infection (Fig. 10B and data not shown) were analyzed. After IFN-α treatment, levels of IFIT2 mRNA were induced, as expected. Interestingly, the levels of IFIT2 mRNA induced were 2.20- to 5.81-fold higher in the PBMCs from patients 64, 68, 87, 21, 23, and 44 (infected with viruses encoding the I64-NS1 protein) than from patient 85 (infected with the virus encoding the T64-NS1 protein) (Fig. 10A). After infection with virus r85, no significant increases (less than 2-fold) in IFN-β expression were observed (data not shown). However, expression of IFN-λ1 was induced, and again, the levels of IFN-λ1 mRNA induction were 2.31- to 9.2-fold higher in the PBMCs from patients 64, 68, 87, 21, 23, and 44 (infected with viruses encoding the I64-NS1 protein) compared to patient 85 (infected with the virus encoding the T64-NS1 protein) (Fig. 10B). As controls, the levels of GAPDH expression were similar in all samples (data not shown), and the levels of viral gene M were similar after infection with virus r85 (Fig. 10C). These data suggested that patient 85 presents a defect in the induction of IFN responses, providing an explanation of how a highly attenuated, IFN-sensitive virus was able to infect and cause symptoms in this and perhaps other human subjects with innate immunity defects.

## DISCUSSION

Influenza virus NS1 protein counteracts host innate immune responses, allowing the virus to replicate efficiently even in IFN-competent systems (8, 9, 38). In this work, we show quantitative differences in the innate immune responses induced after infection with human H3N2 IAV circulating during the 2012-2013 flu season in Rochester (NY), which have been correlated with differences in NS1 amino acid sequences. Furthermore, we show for the first time that a particular mutation (I64T) in the dsRNA-binding domain decreases NS1-mediated host gene expression inhibition by affecting the NS1 binding to CPSF30. Interestingly, a recombinant PR8 virus incorporating the mutation T64 (r85) was fully attenuated *in vivo*, probably because this virus is highly sensitive to IFN responses. Furthermore, we show that the patient infected with this virus presents a defect in the induction of type I IFN responses, probably explaining why this subject was infected with the highly sensitive IFN virus and developed flu symptoms.

Some reports have shown variability in the NS1 proteins from seasonal IAVs (43–45), and that IAV strains circulating in humans in different seasons differ in the ability of their NS1 proteins to block innate immune responses (44, 46). In this work, transcriptome analysis of cells infected with viruses circulating in the same season and the same area showed differential induction of innate immune responses, although the viruses grew to similar extents (Fig. 1). Given the previous reports and the fact that two of these viruses (65 and 85) presented variations in three amino acid positions in the NS1 protein (Table 4), we first analyzed the effect of these mutations in type I IFN responses by expressing the different NS1 variants in all of the different possible combinations. IAV H3N2 NS1 proteins of recently circulating viruses blocked general gene expression, as previously shown for other influenza virus NS1 proteins (16–18), although to different extents (Fig. 2). Whereas mutations in positions 26 and 224 had minor effects on the ability of NS1 to suppress gene expression, mutation I64T strongly decreased the ability of NS1 to inhibit general gene expression either in the H3N2 (Fig. 2) or in H1N1 1918 (Fig. 5) NS1 backgrounds, demonstrating the key role of this amino acid residue in affecting general gene expression.

By blocking gene expression in infected cells, the NS1 protein suppresses the expression of type I IFN and type I IFN-stimulated genes, most of which display antiviral activity (16, 18, 47). To analyze the effect of these mutations in counteracting type I IFN responses, two complementary experiments were performed (Fig. 3 and 4). Both of them showed that mutation I64T, which impaired the ability of NS1 to inhibit host gene expression, also impaired the ability of NS1 to counteract host innate immune responses. Similar to these data, previous results with NS1 proteins from H1N1, H7N9, and H5N1 viruses showed that decreasing NS1-mediated inhibition of host gene expression correlates with increased innate immune responses after infection (20–22). Interestingly, mutation I64T conferred an IFN-hyperinducer phenotype in a screening in which the authors passaged an IAV H3N2 virus in type I IFN-incompetent cells (48). Also, in a study involving random mutagenesis, rescue of replication-proficient mutants in type I IFN response-deficient cells, and subsequent screening for viruses with an enhanced capacity to induce type I IFN after infection, a similar mutation (I68T), pointing in the same direction of the α-helix in the NS1 protein crystal structure as the mutation I64T (Fig. 6C and data not shown) and affecting the ability of NS1 protein to counteract IFN responses, was found (49). However, in both studies the mechanism leading to increased IFN induction, as well as virus pathogenesis, was not analyzed.

Influenza virus NS1 protein inhibits host gene expression and, therefore, type I IFN and IFN-stimulated genes by binding and inhibiting the cellular factor CPSF30 (16–18). To analyze the molecular mechanism by which mutation I64T decreases the ability of NS1 to block gene expression, the binding of NS1 to CPSF30 was analyzed (Fig. 6A). NS1 contains I64 bound to CPSF30. This result is consistent with previous results showing that NS1 proteins encoding amino acids F103 and M106 bind CPSF30 (16, 17, 20, 21). Interestingly, mutation T64 decreased the binding of NS1 to CPSF30 (Fig. 6A), providing an explanation for the decreased NS1-mediated inhibition of host gene expression (Fig. 2). It has been described that the binding site for CPSF30 is centered around amino acid 186, and that mutation in amino acids 184 to 188 leads to a virus highly impaired in growth (18). In addition, amino acid residues 103 and 106 (16, 17, 20, 21) and residues 108, 125, and 189 (22) are important for NS1 binding to CPSF30 for PR8 and pH1N1 viruses, respectively. It is worth mentioning that as far as we know, this is the first amino acid in the dsRNA-binding domain of IAV NS1 that has been shown to play a role in the interaction with CPSF30 (Fig. 6A) and inhibition of host protein expression (Fig. 2 and 5) without significantly affecting dsRNA binding (Fig. 6B). A crystal structure of NS1 bound to CPSF30 has been reported; however, only the NS1 effector domain (amino acids 85 to 215) was expressed (16). Therefore, whether the mutation I64T affects the binding to CPSF30 directly or affects the conformational structure of amino acids relevant for the binding should be further studied.

To analyze the effect of the I64T mutation on virus growth and virulence, viruses encoding the NS1 proteins from patients 85 and 87 (differing only at amino acid position 64) were rescued. To this end, we used a virus containing a split NS segment (27). Interestingly, r87 grew to high titers both *in vitro* and *in vivo*. However, r85 grew to lower titers than r87 in the human A549 cells (Fig. 7B). Similarly, in mice, r85 was highly impaired in growth and was fully attenuated compared to r87, which was highly virulent, killing 100% of the mice (Fig. 8). Our results indicate that these differences are due to the higher IFN-β induction at early time points after infection with r85 (Fig. 8 and 9D) and to the type I IFN-sensitive phenotype provided by NS1-T64 compared to NS1-I64 (Fig. 7C). In agreement with our results, increasing the binding of NS1 protein to CPSF30 in H7N9 and in H5N1 NS1 proteins by introducing the mutations L103F and I106M led to decreased type I IFN responses after infection, augmented virus titers *in vivo*, and increased virulence in mice (21, 40).

It was surprising that subject 85 was infected and reporting flu-like symptoms with a virus encoding the T64-NS1 protein (see Table S5 in the supplemental material), as we have shown that this mutation is quite rare in the human population (0.03%), most probably because this virus is highly sensitive to IFN responses and is highly attenuated, at least in mice (Fig. 8). PBMCs from subject 85 were treated with type I IFN and infected with r85, as it was previously shown that influenza virus induced the synthesis of IFN after PBMC infection (50) and that type I IFN treatment of PBMCs induced the expression of ISGs (51). Interestingly, induction of ISGs and IFN-λ1 in PBMCs from subject 85 was significantly decreased compared to that of other subjects (Fig. 10), providing a possible explanation for why this virus could infect this subject. This suggests that viruses with an impaired ability to antagonize interferon responses can persist in the population by infecting subjects with interferon defects. At this point, the exact cause of the reduced interferon responses in this subject is unknown. It is possible there are genetic factors that are responsible, but it was not possible to investigate this directly given the consent requirements for genetic testing.

Altogether, analyzing the NS1 sequences from influenza viruses infecting subjects and the effect of mutations on NS1 protein functions represents a feasible strategy to identify NS1 mutations affecting innate immune responses and pathogenicity *in vivo*. In addition, screening viral sequences in patients infected with viruses bearing mutations that impact IFN antagonism might be a valid strategy to identify patients with possible innate immune defects.

## Supplementary Material

Supplemental material
