# Supplementary material for: NS1 Protein Mutation I64T Affects Interferon Responses and Virulence of Circulating H3N2 Human Influenza A Viruses
Source: J Virol. 2016 Oct 14;90(21):9693–711. doi: 10.1128/JVI.01039-16 (PMC5068522; doi:10.1128/JVI.01039-16)
Supplement: Supplemental material [file supp_90_21_9693__index.html]

Supplemental material 

# NS1 Protein Mutation I64T Affects Interferon Responses and Virulence of Circulating H3N2 Human Influenza A Viruses

## Supplemental material

- Supplemental file 1 -

  Table S1 (Differentially expressed genes in MDCK cells infected with virus 65 versus those in mock-infected cells.)

  Table S2 (Differentially expressed genes in MDCK cells infected with virus 85 versus those in mock-infected cells.)

  Table S3 (Differentially expressed genes in MDCK cells infected with virus 65 versus those in cells infected with virus 85.)

  Table S4 (Amino acid changes between viruses isolated from patients 65 and 85.)

  Table S5 (Frequency of amino acid changes in MDCK cells infected with viruses isolated from patients 65 and 85.)

  Table S6 (Amino acid changes at position 64 in NS1 proteins from circulating influenza A viruses.)

  PDF, 360K
