# Supplementary material for: NS1 Protein Mutation I64T Affects Interferon Responses and Virulence of Circulating H3N2 Human Influenza A Viruses
Source: J Virol. 2016 Oct 14;90(21):9693–711. doi: 10.1128/JVI.01039-16 (PMC5068522; doi:10.1128/JVI.01039-16)
Supplement: Supplemental material [file JVI.01039-16_zjv999182050so1.pdf]

SUPPLEMENTARY TABLE 1. Differentially expressed genes in MDCK cells infected with virus 65 vs mock-infected cells.

gene_id	gene	value_1 (MOCK)	value_2 (65)	p_value	q_value	FOLD CHANGE 65 VS MOCK
ENSCAFG00000001653	IFNB1	0	2.54138	2.16E-12	7.40E-10	#DIV/0!
ENSCAFG00000013842	MAGEB16	0	0.196924	2.25E-09	5.53E-07	#DIV/0!
ENSCAFG00000021492	U3	0	3.65444	0.000444878	0.0295017	#DIV/0!
ENSCAFG00000022153	5S_rRNA	0	22.9479	0.000444878	0.0295017	#DIV/0!
ENSCAFG00000028395	SNORA31	0	27.752	1.80E-05	0.00202447	#DIV/0!
ENSCAFG00000009617	IFIT1	0.329682	23.2887	0	0	70.63988935
ENSCAFG00000012657	IRGM	0.478531	19.8128	0	0	41.40337826
ENSCAFG00000000498	ENSCAFG00000000498	0.504054	18.0162	0	0	35.74259901
ENSCAFG00000000502	ENSCAFG00000000502	0.899956	26.0393	0	0	28.93397011
ENSCAFG00000008584	CXL10_CANFA	3.73629	96.6783	0	0	25.87548076
ENSCAFG00000005750	IDO1	0.652966	16.3442	0	0	25.03070604
ENSCAFG00000028220	RNaseP_nuc	0.14277	3.1038	1.18E-06	0.000184692	21.73986132
ENSCAFG00000028829	ENSCAFG00000028829	0.564206	11.8413	0	0	20.9875471
ENSCAFG00000030746	ENSCAFG00000030746	1.26657	26.4246	0	0	20.8631185
ENSCAFG00000022080	U3	0.619814	11.9284	0.000131401	0.0110343	19.24512838
ENSCAFG00000011481	IL29L	0.173132	3.29086	1.09E-13	4.16E-11	19.00780907
ENSCAFG00000000497	ENSCAFG00000000497	0.679403	12.4006	0	0	18.25220083
ENSCAFG00000028671	ENSCAFG00000028671	0.661289	11.6463	0	0	17.61151327
ENSCAFG00000024867	TRIM22	0.048907	0.82879	1.02E-12	3.63E-10	16.94624491
ENSCAFG00000018171	CCL5	0.181859	2.59183	1.11E-14	4.50E-12	14.25186546
ENSCAFG00000010511	Q2KM15_CANFA	0.34898	4.91644	0	0	14.08802797
ENSCAFG00000024116	ENSCAFG00000024116	0.10992	1.53478	0.000710472	0.0422506	13.96270015
ENSCAFG00000024087	MB21D1	0.0800542	1.11472	2.66E-13	9.82E-11	13.92456611
ENSCAFG00000005582	ENSCAFG00000005582	0.0878651	1.16403	3.47E-06	0.000487824	13.2479221
ENSCAFG00000006550	ENSCAFG00000006550	0.134884	1.76456	0	0	13.08205569
ENSCAFG00000010172	MX1_CANFA	10.2442	128.655	0	0	12.55881377
ENSCAFG00000009612	IFIT2	0.578194	6.69669	0	0	11.58208145
ENSCAFG00000023783	ZBP1	0.0775736	0.868522	3.13E-12	1.06E-09	11.1961028
ENSCAFG00000008933	NLRC5	0.0851416	0.950626	0	0	11.16523533
ENSCAFG00000031614	IFIT3	2.54578	27.2511	0	0	10.70442065
ENSCAFG00000023063	APOL5	0.768167	8.15542	0	0	10.61672787
ENSCAFG000000032	RSAD2	19.636	203.488	0	0	10.36300672

93

ENSCAFG00000001689	ENSCAFG00000001689	0.0179463	0.178365	5.52E-05	0.00528391	9.938817472
ENSCAFG00000011681	EPSTI1	1.14303	10.7632	2.81E-05	0.00299584	9.416375773
ENSCAFG00000031880	5S_rRNA	5.49831	50.5735	0.000607677	0.0374682	9.198008115
ENSCAFG00000025851	U3	0.972923	8.5113	1.84E-05	0.00205913	8.748174316
ENSCAFG00000012046	IFI6	0.196986	1.58598	6.43E-08	1.28E-05	8.051232067
ENSCAFG00000001394	ETV7	0.436106	3.15971	0	0	7.245279817
ENSCAFG00000025983	RNase_MRP	0.631564	4.53326	0.000115265	0.00990045	7.177831542
ENSCAFG00000031735	C4NZX1_CANFA	0.079105	0.566393	0	0	7.16001517
ENSCAFG00000031602	XAF1	0.0334252	0.239256	2.68E-05	0.00287281	7.157952682
ENSCAFG00000010167	MX2_CANFA	13.5261	96.1532	0	0	7.108715742
ENSCAFG00000009800	ENSCAFG00000009800	2.11664	14.8445	0	0	7.013237962
ENSCAFG00000001807	DDX58	7.67502	53.5688	0	0	6.979630021
ENSCAFG00000005588	ENSCAFG00000005588	0.105688	0.715677	0.000191124	0.0150472	6.771601317
ENSCAFG00000014220	C1R	0.061293	0.407105	4.26E-06	0.000587122	6.641949325
ENSCAFG00000028753	5S_rRNA	11.0144	63.2912	0.000222745	0.0169069	5.746223126
ENSCAFG00000031253	5S_rRNA	11.0144	63.2912	0.000222745	0.0169069	5.746223126
ENSCAFG00000020204	ENSCAFG00000020204	0.2399	1.35786	2.79E-07	5.00E-05	5.660108378
ENSCAFG00000029051	ENSCAFG00000029051	5.32113	29.7013	0	0	5.581765527
ENSCAFG00000008285	HNF4G	0.0473915	0.262921	1.19E-08	2.65E-06	5.547851408
ENSCAFG00000011525	ISG20	6.35843	35.0156	0	0	5.506956906
ENSCAFG00000006212	SEMA3A	0.140336	0.77038	8.55E-14	3.29E-11	5.489539391
ENSCAFG00000021718	U3	3.41308	18.4393	4.94E-05	0.00482246	5.402539642
ENSCAFG00000019348	ISG15	80.7563	435.551	0	0	5.393399648
ENSCAFG00000018916	SOCS1	0.187643	1.01057	0.000422111	0.0284333	5.38559925
ENSCAFG00000015087	RARRES3	1.6802	8.89658	0	0	5.294952982
ENSCAFG00000020954	U3	3.55071	18.5515	3.75E-05	0.00385151	5.224729702
ENSCAFG00000007099	ENSCAFG00000007099	0.248181	1.27992	5.50E-10	1.48E-07	5.157203815
ENSCAFG00000014864	CASP12	0.0978787	0.500158	8.67E-07	0.000139219	5.109977963
ENSCAFG00000022297	U3	4.48202	22.528	1.17E-05	0.00141358	5.026305104
ENSCAFG00000014017	BATF2	0.769299	3.80272	2.00E-15	8.54E-13	4.943097547
ENSCAFG00000021979	U3	3.87286	18.987	6.84E-07	0.000112482	4.902578456
ENSCAFG00000013909	Q7YSA1_CANFA	0.424097	2.01528	0	0	4.751931751
ENSCAFG00000028873	CMPK2	22.7643	105.005	0	0	4.612704981
ENSCAFG00000008659	SAMHD1	4.7475	21.7148	0	0	4.573944181

ENSCAFG00000020152	IL34	0.31493	1.43535	1.52E-13	5.69E-11	4.557679484
ENSCAFG00000002120	CD274	0.0430979	0.192439	0.000746317	0.0438856	4.465159555
ENSCAFG00000024540	GBP5	0.4167	1.84438	0	0	4.426157907
ENSCAFG00000005335	CAPN14	0.151372	0.669253	4.86E-11	1.46E-08	4.421246994
ENSCAFG00000030725	PSMB9	0.341535	1.49637	0	0	4.381307919
ENSCAFG00000011953	PARP15	0.549872	2.39912	0	0	4.363051765
ENSCAFG00000025025	TRIM34	0.0829808	0.34529	0.000105514	0.00918892	4.161083046
ENSCAFG00000015720	DHX58	3.81479	14.6428	0	0	3.838428852
ENSCAFG00000020343	IFI44L	4.22825	15.9807	1.62E-09	4.11E-07	3.779506888
ENSCAFG00000014584	TMEM106A	1.03823	3.91626	2.00E-14	7.99E-12	3.772054362
ENSCAFG00000018872	PLAC1	0.282719	1.03249	0.000412742	0.0279626	3.65200075
ENSCAFG00000031353	BST2	11.6528	41.5154	0	0	3.562697377
ENSCAFG00000020200	GBP6	0.138429	0.485786	1.89E-12	6.51E-10	3.509279125
ENSCAFG00000005675	RNF213	2.29046	7.96844	0	0	3.47896929
ENSCAFG00000011970	PARP14	3.10362	10.7863	0	0	3.47539325
ENSCAFG00000007406	H6BA88_CANFA	3.42463	11.6916	0	0	3.413974648
ENSCAFG00000022709	ENSCAFG00000022709	54.0088	181.776	0	0	3.365673742
ENSCAFG00000010588	MARCKSL1	19.3409	64.7839	0	0	3.349580423
ENSCAFG00000004776	TRANK1	0.90297	2.97385	4.00E-15	1.66E-12	3.293409526
ENSCAFG00000014860	CASP4	0.11225	0.362122	0.000168847	0.0136776	3.22603118
ENSCAFG00000022461	7SK	1.12404	3.59688	0.000476964	0.0310811	3.199957297
ENSCAFG00000019054	ENSCAFG00000019054	32.3547	103.42	0	0	3.196444411
ENSCAFG00000017556	TEX14	0.0439526	0.140246	0.000722586	0.0427577	3.190846503
ENSCAFG00000007803	GALNTL6	0.105383	0.331789	4.29E-06	0.000591606	3.148411034
ENSCAFG00000009003	DDX60	18.5555	56.8746	2.22E-16	1.01E-13	3.065107381
ENSCAFG00000016252	ENSCAFG00000016252	10.7661	32.1496	0	0	2.986188128
ENSCAFG00000010438	IFIH1	7.36878	22.0003	0	0	2.985609558
ENSCAFG00000008401	P2RX7	0.0395829	0.117886	6.57E-05	0.00614618	2.978205235
ENSCAFG00000024646	ERAP2	0.976777	2.901	8.85E-12	2.91E-09	2.969971652
ENSCAFG00000000832	TAP1	6.13683	17.7897	0	0	2.89884191
ENSCAFG00000020342	IFI44	39.0991	111.116	1.15E-10	3.30E-08	2.841906847
ENSCAFG00000015383	TNFSF10	8.11151	22.9811	0	0	2.833146973
ENSCAFG00000011940	PARP9	5.45093	15.3374	0	0	2.813721695
ENSCAFG00000012968	HELZ2	2.2539	6.32312	5.01E-05	0.00487354	2.80541284
ENSCAFG00000030686	GLIPR2	1.5232	4.09841	3.73E-14	1.46E-11	2.690657826

ENSCAFG00000014346	C1S	0.124984	0.335624	1.18E-05	0.00142476	2.685335723
ENSCAFG00000025373	ENSCAFG00000025373	1.45599	3.88575	5.21E-11	1.55E-08	2.66880267
ENSCAFG00000023556	OAS1	26.5648	70.1947	4.44E-15	1.83E-12	2.6423952
ENSCAFG00000023062	FYB	0.19149	0.505034	2.42E-06	0.000354086	2.637390986
ENSCAFG00000017632	IFI27	10.296	26.8588	2.40E-05	0.00260782	2.608663559
ENSCAFG00000007151	ENSCAFG00000007151	13.2222	34.4396	4.44E-16	1.97E-13	2.604680008
ENSCAFG00000011948	DTX3L	5.51281	14.3287	4.44E-16	1.97E-13	2.599164491
ENSCAFG00000005609	XDH	0.0670418	0.174128	0.000107122	0.00931166	2.597304965
ENSCAFG00000031100	ENSCAFG00000031100	9.25773	23.6066	5.77E-15	2.37E-12	2.549933947
ENSCAFG00000014624	IFI35	7.09134	18.014	4.17E-14	1.63E-11	2.540281526
ENSCAFG00000017916	SEMA7A	1.60943	4.03388	1.88E-11	5.87E-09	2.506402888
ENSCAFG00000017781	S1PR5	0.498037	1.24426	0.000203458	0.015679	2.498328437
ENSCAFG00000018752	IL7R	0.445097	1.08857	1.07E-05	0.001311	2.445691613
ENSCAFG00000018405	FST	0.872696	2.11756	3.90E-08	8.08E-06	2.426457781
ENSCAFG00000022711	ENSCAFG00000022711	46.7491	112.947	1.09E-13	4.16E-11	2.416025121
ENSCAFG00000013633	B2M	165.532	390.038	3.29E-12	1.11E-09	2.356269483
ENSCAFG00000002007	SAMD9L	30.302	71.0416	1.57E-11	4.97E-09	2.344452511
ENSCAFG00000002416	TDRD7_CANFA	3.47456	8.07548	1.33E-12	4.69E-10	2.324173421
ENSCAFG00000031894	FAM46A	1.66953	3.81957	5.74E-11	1.71E-08	2.28781154
ENSCAFG00000015400	PARP11	3.02636	6.91318	2.51E-08	5.41E-06	2.284321759
ENSCAFG00000009243	SLC38A4	6.58354	14.6567	4.38E-11	1.32E-08	2.226264289
ENSCAFG00000022010	7SK	3.39039	7.52705	0.000298529	0.0214628	2.22011332
ENSCAFG00000011857	SLAMF9	5.6474	12.4147	2.76E-09	6.66E-07	2.198303644
ENSCAFG00000008929	OAS3	9.57171	20.9069	2.26E-11	6.92E-09	2.184238762
ENSCAFG00000003997	PARP12	4.81979	10.1918	2.30E-10	6.47E-08	2.114573457
ENSCAFG00000018641	LGALS9	32.5649	68.6151	5.44E-08	1.10E-05	2.107026277
ENSCAFG00000002471	TRIM14	8.64502	18.1429	0.000154162	0.0126516	2.098653329
ENSCAFG00000009797	STAT1	14.1768	29.4718	8.46E-09	1.91E-06	2.078875346
ENSCAFG00000000823	H8ZYZ2_CANFA	3.24696	6.69564	1.05E-09	2.73E-07	2.062125804
ENSCAFG00000013889	RTP4	11.6226	23.7702	1.90E-09	4.76E-07	2.045170616
ENSCAFG00000005605	RELT	1.21828	2.47666	5.59E-07	9.39E-05	2.032915258
ENSCAFG00000013121	A5H028_CANFA	3.32669	6.69003	4.04E-09	9.46E-07	2.011016957
ENSCAFG00000014965	GCH1	6.07557	12.1871	9.68E-07	0.000153904	2.005918786
ENSCAFG00000018381	ESM1	8.00889	16.0093	3.90E-09	9.18E-07	1.998941177
ENSCAFG00000006025	TRIM21	1.39733	2.79117	8.80E-08	1.71E-05	1.99750238

ENSCAFG00000004351	PHF11	3.20759	6.3783	2.29E-06	0.000336557	1.988502271
ENSCAFG00000007839	UBE2L6	23.2095	46.0818	5.43E-09	1.26E-06	1.985471466
ENSCAFG00000022721	ENSCAFG00000022721	858.156	1702.6	0.000408635	0.0277243	1.984021553
ENSCAFG00000020294	HAS3	0.434311	0.861664	0.000639103	0.03882	1.983979222
ENSCAFG00000032012	OGFR	2.15679	4.2121	9.38E-05	0.00829523	1.952948595
ENSCAFG00000009781	HERC6	18.1894	35.5022	1.17E-08	2.59E-06	1.951807096
ENSCAFG00000004966	MYD88	15.7965	30.6316	1.42E-08	3.12E-06	1.939138417
ENSCAFG00000006051	Q2KM13_CANFA	8.15829	15.8188	3.69E-08	7.70E-06	1.938984763
ENSCAFG00000017836	PIGW	1.24236	2.40654	9.07E-05	0.00807252	1.93707138
ENSCAFG00000017169	ADAR	10.8971	21.0575	3.95E-07	6.87E-05	1.932394857
ENSCAFG00000006546	ARID5A	0.308969	0.595582	0.000267006	0.0195775	1.927643226
ENSCAFG00000029394	HAS2	2.64263	5.01122	1.84E-07	3.43E-05	1.896300277
ENSCAFG00000017921	C19orf66	10.083	19.1181	9.81E-07	0.000155413	1.896072597
ENSCAFG00000006638	SNAI2	2.5112	4.69507	6.15E-06	0.000805716	1.869651959
ENSCAFG00000000851	IRF1	5.04422	9.35472	2.14E-07	3.96E-05	1.854542427
ENSCAFG00000028442	5_8S_rRNA	339.376	617.344	4.39E-05	0.00437998	1.81905615
ENSCAFG00000001704	APOL6	9.13819	16.4633	0.000141052	0.0117087	1.801593094
ENSCAFG00000023107	OAS2	27.3461	48.9417	2.03E-06	0.000301978	1.789714073
ENSCAFG00000020276	CYR61	15.8755	28.1078	9.56E-07	0.000152328	1.770514315
ENSCAFG00000000073	ZCCHC2	2.66964	4.68086	1.63E-05	0.00186722	1.753367495
ENSCAFG00000010717	ENSCAFG00000010717	2.97217	5.18411	2.02E-05	0.0022393	1.744217188
ENSCAFG00000001289	LY6E	8.92011	15.5093	0.000765029	0.0447339	1.738689321
ENSCAFG00000011164	UBA7	5.23727	9.02695	1.25E-05	0.00149317	1.723598363
ENSCAFG00000017707	ENSCAFG00000017707	18.9401	32.572	3.57E-06	0.000500396	1.719737488
ENSCAFG00000000121	ENSCAFG00000000121	11.4711	19.6841	3.58E-06	0.000501149	1.715973185
ENSCAFG00000020333	PSMB10	10.0354	17.1667	7.12E-05	0.00660043	1.710614425
ENSCAFG00000001301	GSDMD	14.3931	24.5487	4.56E-06	0.000624252	1.705588094
ENSCAFG00000015892	CXCL16	7.86556	13.4149	7.28E-05	0.006729	1.705523828
ENSCAFG00000016228	SERPINE2	19.0834	32.4117	6.10E-06	0.000800955	1.698423761
ENSCAFG00000000267	TNFAIP3	2.27906	3.85566	2.32E-05	0.00254043	1.691776434
ENSCAFG00000026794	5_8S_rRNA	349.486	589.244	0.000158848	0.0129909	1.686030342
ENSCAFG00000032746	MXD1	1.11457	1.86389	0.000633024	0.0385754	1.672295145
ENSCAFG00000008181	ENSCAFG00000008181	16.8457	28.1612	8.86E-05	0.00791715	1.671714443
ENSCAFG00000002891	PNPT1	3.37384	5.62662	8.35E-05	0.00756448	1.667719868
ENSCAFG00000013418	NFKBIA	3.66302	6.10206	0.000120599	0.0102928	1.66585495

ENSCAFG00000020110	Q4W6L5_CANFA	24.5056	40.5932	1.50E-05	0.0017428	1.656486681
ENSCAFG00000003189	RHBDL2	5.48348	9.07928	0.000224032	0.0169634	1.655751457
ENSCAFG00000000958	GJA1	14.9757	24.792	2.87E-05	0.00305859	1.655481881
ENSCAFG00000010704	SP140	0.906744	1.48614	0.000301616	0.0216516	1.638985204
ENSCAFG00000001254	EGR1	2.26773	3.69227	0.000178571	0.0143294	1.62817884
ENSCAFG00000025811	5_8S_rRNA	653.407	1063.55	0.000140225	0.0116504	1.627699122
ENSCAFG00000007890	CDKN2AIP	6.27019	10.1689	0.000144462	0.0119812	1.621784986
ENSCAFG00000008830	TRAFD1	8.78899	14.1276	4.59E-05	0.0045569	1.607420193
ENSCAFG00000015757	CNP	17.3658	27.2316	0.000111351	0.00962583	1.568116643
ENSCAFG00000002424	BZW2	17.2697	27.0357	0.000189479	0.0149998	1.565499111
ENSCAFG00000018897	JUN	9.86096	15.4258	0.000628823	0.0384191	1.564330451
ENSCAFG00000029359	KCNK5	2.61544	4.08031	0.000519387	0.0332234	1.560085492
ENSCAFG00000013769	ENSCAFG00000013769	12.6514	19.6379	0.000167194	0.0135788	1.552231374
ENSCAFG00000030110	ENSCAFG00000030110	3.25465	4.96704	0.000462674	0.0303604	1.526136451
ENSCAFG00000030140	ENSCAFG00000030140	10925.3	16603.1	0.000521838	0.0333082	1.519692823
ENSCAFG00000030537	HSPB8	21.1784	14.0631	0.00043621	0.0291532	0.664030333
ENSCAFG00000016241	SMC1A	6.86723	4.52077	0.00033436	0.0235347	0.658310556
ENSCAFG00000000365	C5orf45	16.7855	10.9806	0.000305082	0.0218503	0.654171755
ENSCAFG00000030281	MTHFD2	18.7904	12.1951	0.000721596	0.0427261	0.64900694
ENSCAFG00000000360	SQSTM1	64.2924	41.3892	0.000188335	0.0149431	0.643765048
ENSCAFG00000029920	TSC22D3	2.26476	1.4541	0.000877994	0.0499107	0.642054787
ENSCAFG00000016328	GRB7	5.83107	3.73522	0.000384533	0.0263748	0.64057197
ENSCAFG00000019181	INPP5K	3.74889	2.35782	0.000201378	0.0155872	0.628938166
ENSCAFG00000000420	CPM	15.0279	9.42877	0.000263885	0.0193874	0.62741767
ENSCAFG00000012085	ABCC5	0.883488	0.541613	0.000482675	0.0313879	0.613039453
ENSCAFG00000017891	DEGS2	18.756	11.3749	0.000119301	0.0102005	0.606467264
ENSCAFG00000000569	ZNF608	1.08128	0.650601	0.000352802	0.0245933	0.601695213
ENSCAFG00000032469	DDIT4	20.9744	12.6156	1.42E-05	0.00167376	0.601476085
ENSCAFG00000003749	SLC7A11	4.07679	2.43226	0.000656064	0.0397059	0.59661155
ENSCAFG00000006777	ADAMTS9	1.56612	0.929777	5.49E-05	0.0052675	0.593681838
ENSCAFG00000016984	PER1	1.80365	1.06891	0.000135106	0.0112949	0.592637152
ENSCAFG00000014386	SLC25A42	1.9165	1.12874	0.00023179	0.017389	0.58895904
ENSCAFG00000019937	KIAA0513	3.4021	2.00311	0.000506139	0.0325203	0.588786338
ENSCAFG00000005428	DYRK1B	2.7792	1.62997	0.000342394	0.0240461	0.586488918
ENSCAFG00000008685	CCNG2	6.58725	3.85454	1.82E-05	0.00203711	0.585151619

ENSCAFG00000009313	PHF21A	1.97447	1.15426	0.00044625	0.0295718	0.584592321
ENSCAFG00000020184	CHST4	15.6659	9.06249	5.85E-06	0.000775748	0.578485117
ENSCAFG00000002028	VLDLR	2.68933	1.54213	0.000458251	0.0301057	0.573425351
ENSCAFG00000017937	CYP1A1	20.6017	11.5491	6.82E-07	0.000112482	0.56058966
ENSCAFG00000005595	FARP1	0.976246	0.546777	0.000231144	0.017362	0.560081168
ENSCAFG00000019194	SLC43A2	1.40432	0.763516	0.00048742	0.0316308	0.543690897
ENSCAFG00000008104	ENSCAFG00000008104	7.40467	4.01981	6.77E-05	0.00631322	0.542874969
ENSCAFG00000001095	TEF	2.44444	1.31103	0.000757963	0.0444591	0.53633143
ENSCAFG00000001957	DOCK8	0.234599	0.124914	0.00061539	0.037795	0.532457513
ENSCAFG00000017941	CYP1A2	8.95607	4.76447	8.94E-07	0.000143315	0.53198222
ENSCAFG00000005560	FLRT3	1.08854	0.572539	0.000373515	0.0257318	0.525969647
ENSCAFG00000004655	BCL3	3.88186	2.02789	4.71E-05	0.00464596	0.522401632
ENSCAFG00000005593	NCCRP1	1.23496	0.641229	0.000481746	0.0313492	0.519230582
ENSCAFG00000029146	PPP1R3C	4.2054	2.153	7.11E-07	0.000116847	0.511960812
ENSCAFG00000002727	ABCA1	1.62337	0.826005	2.45E-07	4.46E-05	0.508821156
ENSCAFG00000002147	IL1R1	2.02765	0.98549	1.56E-05	0.00180287	0.486025695
ENSCAFG00000012580	ENSCAFG00000012580	0.449537	0.21369	0.000741475	0.0436575	0.475242305
ENSCAFG00000013923	SUSD2	1.00305	0.462151	0.000472277	0.0308612	0.460745726
ENSCAFG00000012129	GRK5	0.857347	0.388292	2.23E-07	4.08E-05	0.452899468
ENSCAFG00000009418	TP53INP1	1.21425	0.53507	2.43E-05	0.00263046	0.440658843
ENSCAFG00000006674	SPINK5	33.9147	14.555	1.64E-10	4.66E-08	0.429164934
ENSCAFG00000029093	LYPD6	0.524371	0.222029	1.00E-05	0.00124276	0.423419678
ENSCAFG00000011850	KLHL24	5.34059	2.23515	6.59E-10	1.75E-07	0.418521175
ENSCAFG00000001007	FBXO32	0.947766	0.320715	1.38E-06	0.000212528	0.338390489
ENSCAFG00000012255	RAB17	1.31399	0.417218	1.19E-06	0.000185675	0.31751992
ENSCAFG00000008380	AQP5	0.340374	0.0812865	0.000302883	0.0217259	0.238815244
ENSCAFG00000030131	ENSCAFG00000030131	0.305328	0	8.50E-05	0.00766604	0

#DIV/0!, Indicates that no accurate fold change can be calculated as the denominator is 0.

SUPPLEMENTARY TABLE 2. Differentially expressed genes in MDCK cells infected with virus 85 vs mock-infected cells.

gene_id	gene	value_1 (MOCK)	value_2 (85)	p_value	q_value	FOLD CHANGE 85 VS MOCK
ENSCAFG00000001653	IFNB1	0	12.7543	7.79E-22	3.70E-19	#DIV/0!
ENSCAFG000000027	Q95LE4_CANFA	0	0.305077	1.13E-07	2.17E-05	#DIV/0!

33						
ENSCAFG00000013842	MAGEB16	0	0.306107	1.05E-11	3.40E-09	#DIV/0!
ENSCAFG00000018164	CCL4_CANFA	0	0.675799	1.23E-09	3.16E-07	#DIV/0!
ENSCAFG00000022153	5S_rRNA	0	31.3384	4.84E-05	0.00474577	#DIV/0!
ENSCAFG00000024063	ENSCAFG00000024063	0	0.255127	8.61E-05	0.00773195	#DIV/0!
ENSCAFG00000009617	IFIT1	0.329682	57.8476	0	0	175.4648419
ENSCAFG00000011481	IL29	0.173132	26.054	0	0	150.4863341
ENSCAFG00000005582	ENSCAFG00000005582	0.0878651	9.78473	0	0	111.3608247
ENSCAFG00000008584	CXL10_CANFA	3.73629	311.781	0	0	83.44668107
ENSCAFG00000012657	IRGM	0.478531	36.6909	0	0	76.67402948
ENSCAFG00000018171	CCL5	0.181859	12.71	0	0	69.88930985
ENSCAFG00000000498	ENSCAFG00000000498	0.504054	33.5151	0	0	66.49109024
ENSCAFG00000000502	ENSCAFG00000000502	0.899956	46.4019	0	0	51.56018739
ENSCAFG00000005588	ENSCAFG00000005588	0.105688	5.40374	8.88E-16	3.88E-13	51.12917266
ENSCAFG00000005750	IDO1	0.652966	31.3856	0	0	48.06620865
ENSCAFG00000024116	ENSCAFG00000024116	0.10992	4.81009	7.95E-07	0.000129249	43.7599163
ENSCAFG00000006550	ENSCAFG00000006550	0.134884	5.46544	0	0	40.51955755
ENSCAFG00000028829	ENSCAFG00000028829	0.564206	19.0353	0	0	33.73820909
ENSCAFG00000000497	ENSCAFG00000000497	0.679403	21.0005	0	0	30.9102256
ENSCAFG00000012046	IFI6	0.196986	5.74363	0	0	29.15755434
ENSCAFG00000028671	ENSCAFG00000028671	0.661289	19.0004	0	0	28.73236966
ENSCAFG00000024867	TRIM22	0.048907	1.32622	0	0	27.11718159
ENSCAFG00000024087	MB21D1	0.0800542	1.81418	0	0	22.66189657
ENSCAFG00000010511	Q2KM15_CANFA	0.34898	7.84851	0	0	22.48985615
ENSCAFG00000031614	IFIT3	2.54578	54.1892	0	0	21.28589273
ENSCAFG00000009612	IFIT2	0.578194	12.2205	0	0	21.1356396
ENSCAFG00000001689	ENSCAFG00000001689	0.0179463	0.3529	4.71E-08	9.64E-06	19.66422048
ENSCAFG00000028220	RNaseP_nuc	0.14277	2.56642	5.51E-06	0.000735217	17.9759053
ENSCAFG00000030746	ENSCAFG00000030746	1.26657	20.8307	0	0	16.4465446
ENSCAFG00000023783	ZBP1	0.0775736	1.20186	1.33E-15	5.77E-13	15.49315747
ENSCAFG00000010172	MX1_CANFA	10.2442	158.554	0	0	15.47744089
ENSCAFG00000003293	RSAD2	19.636	299.146	0	0	15.23456916
ENSCAFG00000023063	APOL5	0.768167	11.6789	0	0	15.20359505
ENSCAFG00000008933	NLRC5	0.0851416	1.28223	0	0	15.05997068
ENSCAFG00000020204	ENSCAFG00000020204	0.2399	3.5978	0	0	14.99708212

ENSCAFG00000002120	CD274	0.0430979	0.624459	8.72E-11	2.54E-08	14.48931386
ENSCAFG00000008285	HNF4G	0.0473915	0.672163	0	0	14.18319741
ENSCAFG00000020200	GBP6	0.138429	1.80615	0	0	13.04748283
ENSCAFG00000031602	XAF1	0.0334252	0.432491	2.33E-08	5.06E-06	12.93906992
ENSCAFG00000011681	EPSTI1	1.14303	14.747	1.47E-06	0.000224423	12.90167362
ENSCAFG00000001807	DDX58	7.67502	98.9914	0	0	12.89786867
ENSCAFG00000031923	ENSCAFG00000031923	0.0175415	0.218569	8.23E-05	0.00748	12.46010888
ENSCAFG00000006212	SEMA3A	0.140336	1.65008	0	0	11.75806636
ENSCAFG00000013909	Q7YSA1_CANFA	0.424097	4.83915	0	0	11.41047921
ENSCAFG00000018916	SOCS1	0.187643	2.02847	2.48E-07	4.50E-05	10.81026204
ENSCAFG00000003439	IL4I1	0.101971	1.10125	0.000351832	0.0245439	10.79963911
ENSCAFG00000031735	C4NZX1_CANFA	0.079105	0.848155	0	0	10.72188863
ENSCAFG00000001394	ETV7	0.436106	4.46336	0	0	10.234576
ENSCAFG00000015087	RARRES3	1.6802	17.0199	0	0	10.12968694
ENSCAFG00000024540	GBP5	0.4167	4.11102	0	0	9.865658747
ENSCAFG00000009800	ENSCAFG00000009800	2.11664	19.5407	0	0	9.23194308
ENSCAFG00000010167	MX2_CANFA	13.5261	116.098	0	0	8.583257554
ENSCAFG00000014017	BATF2	0.769299	6.47318	0	0	8.414387644
ENSCAFG00000007099	ENSCAFG00000007099	0.248181	1.93581	1.55E-15	6.67E-13	7.799992747
ENSCAFG00000014220	C1R	0.061293	0.476575	2.55E-07	4.62E-05	7.775357708
ENSCAFG00000018405	FST	0.872696	6.61268	0	0	7.577300687
ENSCAFG00000011525	ISG20	6.35843	48.1345	0	0	7.570186351
ENSCAFG00000019348	ISG15	80.7563	596.499	0	0	7.386408243
ENSCAFG00000029051	ENSCAFG00000029051	5.32113	38.8261	0	0	7.296589258
ENSCAFG00000022709	ENSCAFG00000022709	54.0088	334.125	0	0	6.186491831
ENSCAFG00000013585	CRHR1	0.0560401	0.333753	0.000191705	0.0150744	5.955610358
ENSCAFG00000002390	FAM178B	0.0205321	0.121837	5.02E-05	0.00487822	5.933976554
ENSCAFG00000030725	PSMB9	0.341535	2.01453	0	0	5.89845843
ENSCAFG00000019809	GSTM3	0.200798	1.18189	2.15E-07	3.96E-05	5.885965
ENSCAFG00000028873	CMPK2	22.7643	133.752	0	0	5.87551561
ENSCAFG00000025025	TRIM34	0.0829808	0.484445	8.26E-07	0.000133439	5.838037233
ENSCAFG00000010588	MARCKSL1	19.3409	111	0	0	5.739133132
ENSCAFG00000019054	ENSCAFG00000019054	32.3547	181.418	0	0	5.607160629
ENSCAFG00000008659	SAMHD1	4.7475	25.9659	0	0	5.469383886
ENSCAFG00000014584	TMEM106A	1.03823	5.60819	0	0	5.401683635

ENSCAFG00000020152	IL34	0.31493	1.65756	4.44E-16	1.97E-13	5.263264853
ENSCAFG00000007803	GALNTL6	0.105383	0.529441	2.25E-11	6.90E-09	5.02396971
ENSCAFG00000002568	GUCA2A	0.185082	0.923176	0.000116485	0.00999616	4.987929674
ENSCAFG00000008391	HEY1	0.269801	1.31319	3.55E-15	1.48E-12	4.867254013
ENSCAFG00000011953	PARP15	0.549872	2.56923	0	0	4.672414671
ENSCAFG00000000267	TNFAIP3	2.27906	10.5727	0	0	4.639061718
ENSCAFG00000012339	ATF3	1.84996	8.42425	1.21E-12	4.29E-10	4.553747108
ENSCAFG00000018864	GPC3	0.0285625	0.129557	0.00070045	0.0418368	4.535912473
ENSCAFG00000019992	OLFM3	0.0509193	0.222288	0.000857331	0.0490029	4.365495991
ENSCAFG00000013418	NFKBIA	3.66302	15.8084	0	0	4.315673952
ENSCAFG00000020343	IFI44L	4.22825	17.8942	6.94E-11	2.05E-08	4.23205818
ENSCAFG00000014864	CASP12	0.0978787	0.41392	1.77E-05	0.00200102	4.228907822
ENSCAFG00000007406	H6BA88_CANFA	3.42463	14.3068	0	0	4.177619188
ENSCAFG00000004776	TRANK1	0.90297	3.75652	0	0	4.160182509
ENSCAFG00000010955	PTHR_CANFA	0.161748	0.668869	7.87E-05	0.00717501	4.135253604
ENSCAFG00000005609	XDH	0.0670418	0.268798	2.01E-08	4.39E-06	4.009409055
ENSCAFG00000014360	SPTSSB	5.2187	20.8389	1.30E-07	2.47E-05	3.993120892
ENSCAFG00000014346	C1S	0.124984	0.495475	3.21E-10	8.83E-08	3.964307431
ENSCAFG00000030927	NFE2	0.0375165	0.146919	5.37E-06	0.000720295	3.916116909
ENSCAFG00000008401	P2RX7	0.0395829	0.153947	3.86E-07	6.72E-05	3.889229945
ENSCAFG00000031353	BST2	11.6528	44.6349	0	0	3.830401277
ENSCAFG00000022461	7SK	1.12404	4.26079	4.91E-05	0.00479353	3.790603537
ENSCAFG00000011970	PARP14	3.10362	11.6908	0	0	3.766827124
ENSCAFG00000005335	CAPN14	0.151372	0.563121	8.47E-08	1.66E-05	3.720113363
ENSCAFG00000008458	ENSCAFG00000008458	1.82247	6.75795	2.39E-05	0.00259729	3.708126883
ENSCAFG00000031100	ENSCAFG00000031100	9.25773	33.9627	0	0	3.668577502
ENSCAFG00000007151	ENSCAFG00000007151	13.2222	48.3856	0	0	3.659421276
ENSCAFG00000024646	ERAP2	0.976777	3.54703	0	0	3.631361099
ENSCAFG00000025373	ENSCAFG00000025373	1.45599	5.26851	0	0	3.618506995
ENSCAFG00000005675	RNF213	2.29046	8.24419	0	0	3.599359954
ENSCAFG00000014860	CASP4	0.11225	0.39533	0.000135377	0.0113075	3.521870824
ENSCAFG00000010438	IFIH1	7.36878	25.8334	0	0	3.505790646
ENSCAFG00000015720	DHX58	3.81479	13.3675	0	0	3.504124736
ENSCAFG00000009107	NKX3-1	0.661136	2.31005	5.66E-05	0.00539063	3.494061736
ENSCAFG00000000832	TAP1	6.13683	21.333	0	0	3.476224696

ENSCAFG00000000851	IRF1	5.04422	17.4592	0	0	3.461228892
ENSCAFG00000015383	TNFSF10	8.11151	27.8625	0	0	3.434933816
ENSCAFG00000022469	7SK	0.990716	3.38432	0.000330998	0.0234032	3.416034464
ENSCAFG00000009003	DDX60	18.5555	61.4777	2.22E-16	1.01E-13	3.313179381
ENSCAFG00000016252	ENSCAFG00000016252	10.7661	35.5429	0	0	3.301371899
ENSCAFG00000003550	INHBA	1.39371	4.46952	2.22E-16	1.01E-13	3.206922531
ENSCAFG00000009243	SLC38A4	6.58354	20.63	0	0	3.133572516
ENSCAFG00000032746	MXD1	1.11457	3.48984	2.44E-15	1.03E-12	3.131108858
ENSCAFG00000011940	PARP9	5.45093	17.0179	0	0	3.122017711
ENSCAFG00000020342	IFI44	39.0991	122.008	2.38E-14	9.46E-12	3.120481034
ENSCAFG00000003029	IL8_CANFA	0.967579	3.01756	7.01E-13	2.51E-10	3.118670413
ENSCAFG00000031894	FAM46A	1.66953	5.20265	0	0	3.116236306
ENSCAFG00000005605	RELT	1.21828	3.78904	2.22E-16	1.01E-13	3.110155301
ENSCAFG00000009264	BCL2L15	0.989449	3.0231	3.26E-05	0.00339423	3.055336859
ENSCAFG00000018931	CIITA	0.106459	0.324961	0.000156675	0.0128243	3.052452118
ENSCAFG00000012968	HELZ2	2.2539	6.87769	1.04E-05	0.00128167	3.05146191
ENSCAFG00000022711	ENSCAFG00000022711	46.7491	141.867	0	0	3.034646656
ENSCAFG00000021780	7SK	1.09631	3.28439	0.000497696	0.0320541	2.995858836
ENSCAFG00000021575	7SK	2.00292	5.93223	2.86E-05	0.00305232	2.961790785
ENSCAFG00000022010	7SK	3.39039	9.85649	7.17E-07	0.000117602	2.907184719
ENSCAFG00000011948	DTX3L	5.51281	15.8808	0	0	2.880708749
ENSCAFG00000006546	ARID5A	0.308969	0.884768	1.72E-09	4.37E-07	2.863614149
ENSCAFG00000023556	OAS1	26.5648	75.6988	0	0	2.849590435
ENSCAFG00000018752	IL7R	0.445097	1.25131	2.73E-07	4.90E-05	2.81131978
ENSCAFG00000020749	7SK	3.12189	8.74243	2.46E-05	0.00266761	2.800364523
ENSCAFG00000002007	SAMD9L	30.302	84.1491	1.33E-15	5.77E-13	2.777014719
ENSCAFG00000021438	7SK	3.34033	8.95748	2.39E-05	0.00260453	2.68161529
ENSCAFG00000014624	IFI35	7.09134	18.9464	1.55E-15	6.67E-13	2.671765844
ENSCAFG00000022516	7SK	2.11423	5.57838	9.26E-05	0.00821788	2.638492501
ENSCAFG00000001704	APOL6	9.13819	23.9752	7.69E-11	2.25E-08	2.623626779
ENSCAFG00000030686	GLIPR2	1.5232	3.9885	1.92E-13	7.17E-11	2.618500525
ENSCAFG00000026800	5_8S_rRNA	19.4454	50.4762	0.00035743	0.0248055	2.595791293
ENSCAFG00000005937	CATSPERG	0.0936581	0.242679	6.47E-05	0.00606836	2.591115985
ENSCAFG00000022173	7SK	1.93804	5.01447	0.000799968	0.0462873	2.587392417
ENSCAFG00000017836	PIGW	1.24236	3.14118	2.26E-08	4.91E-06	2.528397566

ENSCAFG00000029359	KCNK5	2.61544	6.61047	1.48E-13	5.55E-11	2.527479124
ENSCAFG00000004351	PHF11	3.20759	8.03439	1.50E-10	4.27E-08	2.504805789
ENSCAFG00000001047	NCOA7	0.453507	1.11994	8.37E-09	1.90E-06	2.469509842
ENSCAFG00000002416	TDRD7_CANFA	3.47456	8.5556	3.51E-14	1.38E-11	2.462354946
ENSCAFG00000000686	ENSCAFG00000000686	3.63902	8.9407	4.16E-10	1.13E-07	2.456897736
ENSCAFG00000014195	IQCJ-SCHIP1	1.13701	2.78976	3.14E-09	7.52E-07	2.453593196
ENSCAFG00000023062	FYB	0.19149	0.46936	1.11E-05	0.00135003	2.451094052
ENSCAFG00000015892	CXCL16	7.86556	19.1941	2.09E-11	6.45E-09	2.440271258
ENSCAFG00000013633	B2M	165.532	403.275	5.06E-13	1.82E-10	2.436235894
ENSCAFG00000013889	RTP4	11.6226	27.8321	2.17E-13	8.06E-11	2.39465352
ENSCAFG00000009994	CD40	0.399681	0.954654	3.40E-05	0.00353536	2.388539861
ENSCAFG00000000823	H8ZYZ2_CANFA	3.24696	7.67708	3.89E-13	1.42E-10	2.364390076
ENSCAFG00000002471	TRIM14	8.64502	20.4105	1.05E-05	0.00129052	2.360954631
ENSCAFG00000009797	STAT1	14.1768	33.1163	1.60E-11	5.03E-09	2.335950285
ENSCAFG00000009272	EGR3	0.644901	1.49542	3.70E-05	0.00380052	2.318836535
ENSCAFG00000008305	C21orf91	1.04982	2.42769	4.92E-06	0.000664651	2.31248214
ENSCAFG00000013049	EGR2	0.660852	1.49341	4.65E-05	0.00461052	2.259825195
ENSCAFG00000006025	TRIM21	1.39733	3.14778	2.75E-10	7.62E-08	2.252710527
ENSCAFG00000017921	C19orf66	10.083	22.5388	5.28E-10	1.42E-07	2.235326788
ENSCAFG00000015400	PARP11	3.02636	6.74326	6.86E-08	1.36E-05	2.228175101
ENSCAFG00000012751	AUNIP	1.84455	4.09557	3.21E-07	5.68E-05	2.22036269
ENSCAFG00000017632	IFI27	10.296	22.797	0.00054957	0.034682	2.214160839
ENSCAFG00000011857	SLAMF9	5.6474	12.5009	1.90E-09	4.76E-07	2.213567305
ENSCAFG00000015105	BIRC3_CANFA	1.95107	4.31647	9.82E-10	2.56E-07	2.212360397
ENSCAFG00000023829	APR_CANFA	29.8	65.7013	0.000129115	0.0108812	2.204741611
ENSCAFG00000004575	ZNF296	0.507548	1.11823	7.59E-05	0.00695318	2.203200485
ENSCAFG00000000073	ZCCHC2	2.66964	5.87045	9.34E-10	2.44E-07	2.198966902
ENSCAFG00000017539	LDLR	15.0137	32.7792	7.62E-11	2.24E-08	2.183285932
ENSCAFG00000003997	PARP12	4.81979	10.4747	4.94E-11	1.48E-08	2.17326896
ENSCAFG00000029394	HAS2	2.64263	5.72181	2.71E-10	7.57E-08	2.165195279
ENSCAFG00000004966	MYD88	15.7965	33.7368	8.81E-11	2.56E-08	2.135713607
ENSCAFG00000012607	ENSCAFG00000012607	0.922857	1.96412	1.97E-05	0.00219032	2.128303735
ENSCAFG00000014965	GCH1	6.07557	12.9014	1.00E-07	1.93E-05	2.123488002
ENSCAFG00000004176	PANX1	3.57704	7.59471	1.94E-10	5.48E-08	2.123182855
ENSCAFG00000001254	EGR1	2.26773	4.79722	5.15E-09	1.20E-06	2.115428204

ENSCAFG00000008929	OAS3	9.57171	20.2434	1.43E-10	4.09E-08	2.114919905
ENSCAFG00000018641	LGALS9	32.5649	68.7788	1.38E-07	2.60E-05	2.112053162
ENSCAFG00000031530	EGR4	0.132672	0.280077	0.000355569	0.0246946	2.1110483
ENSCAFG00000007501	DBR1	3.96228	8.28592	6.56E-09	1.50E-06	2.091200016
ENSCAFG00000018381	ESM1	8.00889	16.5223	7.40E-10	1.96E-07	2.062994997
ENSCAFG00000003880	ENSCAFG00000003880	30.0608	61.9422	2.42E-08	5.22E-06	2.060563924
ENSCAFG00000006051	Q2KM13_CANFA	8.15829	16.7934	1.90E-09	4.76E-07	2.058446071
ENSCAFG00000028536	TBX20	0.696752	1.43245	4.48E-07	7.71E-05	2.055896503
ENSCAFG00000017169	ADAR	10.8971	22.2993	3.64E-08	7.64E-06	2.046351782
ENSCAFG00000031460	ICAM1	4.88619	9.90197	3.94E-09	9.24E-07	2.026521687
ENSCAFG00000002891	PNPT1	3.37384	6.82638	4.41E-08	9.08E-06	2.023326536
ENSCAFG00000006638	SNAI2	2.5112	5.0734	3.35E-07	5.92E-05	2.020309016
ENSCAFG00000007686	SLCO5A1	0.258948	0.521269	0.000804013	0.0464754	2.013025781
ENSCAFG00000014297	ZC3H12C	0.79631	1.57548	1.15E-05	0.00139577	1.978475719
ENSCAFG00000009781	HERC6	18.1894	35.8494	7.17E-09	1.63E-06	1.970895137
ENSCAFG00000016228	SERPINE2	19.0834	37.5501	8.47E-09	1.91E-06	1.967683956
ENSCAFG00000028442	5_8S_rRNA	339.376	663.28	4.16E-06	0.000575102	1.954410447
ENSCAFG00000018132	TAF4B	0.493766	0.955792	3.09E-05	0.00324073	1.935718539
ENSCAFG00000004675	TNFSF11	6.08794	11.7753	8.24E-08	1.62E-05	1.93420106
ENSCAFG00000005345	SERTAD1	8.59095	16.5745	1.85E-06	0.000277652	1.929297691
ENSCAFG00000030931	Q8SPY1_CANFA	8.85613	17.044	3.39E-08	7.18E-06	1.924542661
ENSCAFG00000007890	CDKN2AIP	6.27019	11.9529	3.18E-07	5.65E-05	1.90630587
ENSCAFG00000007839	UBE2L6	23.2095	44.0655	4.98E-08	1.01E-05	1.898597557
ENSCAFG00000032012	OGFR	2.15679	4.08609	0.000150113	0.0123952	1.894523806
ENSCAFG00000010704	SP140	0.906744	1.71784	2.43E-06	0.000354414	1.89451488
ENSCAFG00000012130	CFLAR	8.25023	15.6208	1.49E-05	0.00173182	1.893377518
ENSCAFG00000008181	ENSCAFG00000008181	16.8457	31.7789	1.23E-06	0.000190599	1.886469544
ENSCAFG00000002424	BZW2	17.2697	32.3371	1.85E-07	3.45E-05	1.872476071
ENSCAFG00000012168	CASP10	4.06186	7.59567	1.18E-06	0.000184709	1.869997981
ENSCAFG00000009794	EDN1_CANFA	6.196	11.5744	3.75E-07	6.56E-05	1.868043899
ENSCAFG00000013121	A5H028_CANFA	3.32669	6.17323	1.98E-07	3.66E-05	1.855667345
ENSCAFG00000016172	TLR6	0.503228	0.932367	0.000710656	0.0422506	1.852772501
ENSCAFG00000010717	ENSCAFG00000010717	2.97217	5.47084	2.77E-06	0.000400163	1.84068879
ENSCAFG00000017707	ENSCAFG00000017707	18.9401	34.7527	2.20E-07	4.05E-05	1.834874156
ENSCAFG00000006459	CDK17	0.824577	1.51152	0.000493457	0.0318685	1.833085327

ENSCAFG00000020357	DNAJB4	3.17574	5.81432	1.08E-06	0.000170217	1.830855171
ENSCAFG00000017916	SEMA7A	1.60943	2.94357	1.47E-05	0.00172275	1.828951865
ENSCAFG00000007582	SNX25	3.1919	5.80145	6.37E-07	0.000106019	1.817553808
ENSCAFG00000000121	ENSCAFG00000000121	11.4711	20.6911	4.18E-07	7.21E-05	1.803759012
ENSCAFG00000031999	SLC30A1	5.28481	9.5159	5.63E-07	9.45E-05	1.800613456
ENSCAFG00000020276	CYR61	15.8755	28.4362	5.75E-07	9.63E-05	1.791200277
ENSCAFG00000013233	PISD	4.82433	8.58862	0.000792467	0.0459488	1.780272079
ENSCAFG00000030110	ENSCAFG00000030110	3.25465	5.74266	2.35E-06	0.000344567	1.76444779
ENSCAFG00000009660	MORC3	2.5436	4.47617	2.47E-06	0.000358705	1.759777481
ENSCAFG00000008409	VEGFC	1.01982	1.79276	0.000483833	0.0314415	1.757918064
ENSCAFG00000012118	URB2	4.16992	7.28159	2.10E-06	0.000309906	1.746218153
ENSCAFG00000020333	PSMB10	10.0354	17.5144	3.53E-05	0.00364819	1.745261773
ENSCAFG00000008830	TRAFD1	8.78899	15.3329	1.79E-06	0.00026979	1.74455768
ENSCAFG00000023107	OAS2	27.3461	47.6055	5.89E-06	0.000778339	1.740851529
ENSCAFG00000008704	THBS1	11.3652	19.6468	2.78E-06	0.000400346	1.728680534
ENSCAFG00000001301	GSDMD	14.3931	24.8337	2.84E-06	0.000407139	1.725389249
ENSCAFG00000006530	LEPROTL1	34.9484	60.2314	3.91E-05	0.00397248	1.723437983
ENSCAFG00000015757	CNP	17.3658	29.8193	3.46E-06	0.00048668	1.717127918
ENSCAFG00000017492	ARHGAP17	1.85251	3.17472	8.93E-06	0.00111833	1.713739737
ENSCAFG00000003182	ENSCAFG00000003182	10.2498	17.5387	1.15E-05	0.00140271	1.711126071
ENSCAFG00000000300	HIVEP2	1.20958	2.06121	1.46E-05	0.00170657	1.704070835
ENSCAFG00000011164	UBA7	5.23727	8.89134	2.14E-05	0.00235929	1.697705102
ENSCAFG00000000958	GJA1	14.9757	25.3937	1.16E-05	0.00140904	1.695660303
ENSCAFG00000014114	CABYR	1.65756	2.81043	0.000278261	0.020191	1.695522334
ENSCAFG00000015758	EXO1	1.04195	1.76577	0.000103584	0.0090376	1.694678248
ENSCAFG00000002428	ANKMY2	8.08858	13.586	1.16E-05	0.00140637	1.679652053
ENSCAFG00000014184	MGAT2	11.6702	19.5917	1.86E-05	0.00207433	1.678780141
ENSCAFG00000000874	THBS2	1.24969	2.0975	0.000217441	0.0165981	1.678416247
ENSCAFG00000009635	ENSCAFG00000009635	10.1726	17.0567	0.000196001	0.0153098	1.676729646
ENSCAFG00000030140	ENSCAFG00000030140	10925.3	18312.7	1.74E-05	0.00197991	1.676173652
ENSCAFG00000007539	ADML_CANFA	10.8779	18.1918	2.36E-05	0.00258254	1.672363232
ENSCAFG00000003189	RHBDL2	5.48348	9.15688	0.000172508	0.0139142	1.669903054
ENSCAFG00000004401	ENSCAFG00000004401	18.1422	30.2859	3.78E-05	0.00386979	1.66936204
ENSCAFG00000005491	AZI2	6.55074	10.92	6.88E-05	0.0063919	1.666987241
ENSCAFG00000011408	ZNFX1	7.55967	12.5985	6.40E-05	0.00600811	1.666541

ENSCAFG00000020110	Q4W6L5_CANFA	24.5056	40.8361	1.19E-05	0.00143308	1.666398701
ENSCAFG00000001474	DNAJA1	32.4416	54.0205	1.20E-05	0.00144958	1.665161398
ENSCAFG00000010146	NUP153	4.50165	7.49061	8.91E-05	0.00795572	1.663969878
ENSCAFG00000018493	CD320	8.69272	14.4033	0.00022489	0.0169651	1.65693822
ENSCAFG00000011883	ENSCAFG00000011883	9.04409	14.95	0.000168469	0.0136588	1.653013183
ENSCAFG00000026794	5_8S_rRNA	349.486	573.382	0.000347052	0.0242825	1.640643688
ENSCAFG00000002560	ENSCAFG00000002560	33.4841	54.5776	3.19E-05	0.00334277	1.62995571
ENSCAFG00000012689	USF1	7.15162	11.5923	0.000229381	0.0172572	1.620933439
ENSCAFG00000017283	TIPIN	5.07688	8.22685	0.000131858	0.0110627	1.620453901
ENSCAFG00000025811	5_8S_rRNA	653.407	1055.15	0.000181752	0.0145227	1.614843428
ENSCAFG00000004402	PRSS23	4.9673	8.00877	0.000540561	0.0342215	1.612298432
ENSCAFG00000011157	CASP7	2.23831	3.6	0.000451057	0.0297644	1.608356305
ENSCAFG00000015272	FGFBP1	103.563	166.44	5.54E-05	0.00528757	1.607137684
ENSCAFG00000013747	TAB3	1.59131	2.55687	0.000187284	0.0148781	1.606770522
ENSCAFG00000011445	B4GALT5	9.8728	15.8457	0.000191199	0.0150472	1.604985414
ENSCAFG00000018340	ENSCAFG00000018340	31.0937	49.899	4.88E-05	0.0047744	1.60479454
ENSCAFG00000005226	FAM208B	2.77792	4.44828	5.87E-05	0.00556137	1.601298814
ENSCAFG00000010961	LGALS8	9.01006	14.4215	0.000437309	0.0291851	1.600599774
ENSCAFG00000008528	GPR87	9.66029	15.4382	0.000249303	0.0184806	1.598109374
ENSCAFG00000015201	SNAP29	18.5946	29.6593	0.000868137	0.0494401	1.5950491
ENSCAFG00000006434	SHQ1	1.86875	2.96216	0.000203564	0.015679	1.585102341
ENSCAFG00000007738	LSM6	15.9253	25.2117	0.000347612	0.0243036	1.583122453
ENSCAFG00000010010	N4BP1	6.14969	9.70531	8.92E-05	0.00795572	1.578178737
ENSCAFG00000016259	ENSCAFG00000016259	12.5467	19.7665	0.000174336	0.0140255	1.575434178
ENSCAFG00000020052	FRRS1	2.50401	3.94167	0.000169046	0.013682	1.574143075
ENSCAFG00000001818	LOC474739	27.1025	42.5495	0.000130606	0.0109871	1.569947422
ENSCAFG00000018897	JUN	9.86096	15.4104	0.000623653	0.0382275	1.562768737
ENSCAFG00000017306	NFATC2IP	5.66185	8.75146	0.000240197	0.0179274	1.54568913
ENSCAFG00000013769	ENSCAFG00000013769	12.6514	19.5367	0.00019941	0.0155115	1.544232259
ENSCAFG00000017983	PPCDC	3.35071	5.11829	0.000827063	0.0475331	1.527524017
ENSCAFG00000001667	RBM28	4.47198	6.73708	0.000610011	0.0375875	1.506509421
ENSCAFG00000003318	ENSCAFG00000003318	969.096	1441.81	0.000649114	0.0393518	1.487788619
ENSCAFG00000019835	SORT1	11.3601	7.65599	0.00077946	0.0453284	0.673936849
ENSCAFG00000028490	ENSCAFG00000028490	21.1203	14.2283	0.00072599	0.0429051	0.673678878
ENSCAFG00000019838	Q38IV4_CANFA	13.4119	9.00299	0.00070934	0.0422506	0.671268799

ENSCAFG00000005843	Q2PPL3_CANFA	8.73037	5.85504	0.00059747	0.0370047	0.670651988
ENSCAFG00000011615	GOLGB1	3.94428	2.64382	0.000586918	0.0364755	0.67029217
ENSCAFG00000031357	ENSCAFG00000031357	106.535	71.1799	0.000646833	0.0392388	0.668136293
ENSCAFG00000007383	EIF2AK3	12.723	8.49514	0.000520269	0.0332572	0.667699442
ENSCAFG00000018173	LAMA3	6.8059	4.53688	0.000506317	0.0325203	0.666609853
ENSCAFG00000017162	PBXIP1	5.81477	3.87535	0.000656223	0.0397059	0.666466601
ENSCAFG00000025482	ENSCAFG00000025482	109.234	72.7758	0.000488336	0.0316541	0.666237618
ENSCAFG00000019379	ABCA3	4.70102	3.13076	0.000661493	0.039999	0.665974618
ENSCAFG00000018877	NPR3	13.6193	9.06666	0.000536777	0.0340576	0.665721439
ENSCAFG00000001601	PLIN2	12.2845	8.17351	0.000597797	0.0370047	0.665351459
ENSCAFG00000005575	ENSCAFG00000005575	20.1713	13.4093	0.000463558	0.0303972	0.664771234
ENSCAFG00000016582	KIAA0247	7.44885	4.94972	0.000485238	0.031511	0.664494519
ENSCAFG00000001585	FLNC	6.65593	4.41843	0.000488614	0.0316541	0.663833604
ENSCAFG00000017094	ZNF609	3.84145	2.54935	0.000775299	0.0451659	0.663642635
ENSCAFG00000018300	PPP1R13B	7.13228	4.72306	0.000564556	0.0354136	0.662208999
ENSCAFG00000029313	CLDN1	58.2776	38.5863	0.000624661	0.0382644	0.662112029
ENSCAFG00000010452	KDM5B	7.25203	4.79871	0.00044193	0.0293891	0.661705757
ENSCAFG00000004322	APMAP	19.3873	12.8082	0.000517059	0.0330971	0.660648982
ENSCAFG00000003874	ENSCAFG00000003874	27.7729	18.3355	0.000375971	0.025863	0.66019393
ENSCAFG00000028632	ENSCAFG00000028632	51.7115	34.1275	0.000492947	0.0318575	0.659959583
ENSCAFG00000006293	Q3HTT7_CANFA	11.322	7.45787	0.000340025	0.0238977	0.658706059
ENSCAFG00000009654	RBL2	5.59262	3.68037	0.000629365	0.038421	0.658076179
ENSCAFG00000024263	CLDN9	54.0896	35.5649	0.00049935	0.0321386	0.657518266
ENSCAFG00000009104	STC1	43.7286	28.7426	0.000471933	0.0308602	0.657295226
ENSCAFG00000018126	ULK2	5.36498	3.52553	0.000369838	0.0254971	0.657137585
ENSCAFG00000008869	TGM2	27.3926	17.9778	0.000297878	0.0214324	0.656301337
ENSCAFG00000031747	FBXO42	2.60788	1.71106	0.00085052	0.0486729	0.656111478
ENSCAFG00000010100	TEX264	18.9997	12.4507	0.000421014	0.0284006	0.655310347
ENSCAFG00000015495	SLC3A2	10.5523	6.90075	0.000671496	0.0403959	0.653956957
ENSCAFG00000031039	YPEL5	7.70754	5.03883	0.000761331	0.044601	0.653753338
ENSCAFG00000005069	PTPRF	20.3274	13.2809	0.00075969	0.0445326	0.653349666
ENSCAFG00000030281	MTHFD2	18.7904	12.2654	0.000849304	0.0486452	0.652748212
ENSCAFG00000019432	BTBD2	3.13881	2.04758	0.000734155	0.043306	0.652342767
ENSCAFG00000028617	TMPRSS11E	93.0126	60.6543	0.00027425	0.0199617	0.652108424
ENSCAFG00000030885	C7orf60	5.3428	3.48378	0.000662488	0.0400242	0.652051359

ENSCAFG00000031798	ZNF436	2.66418	1.7355	0.000877975	0.0499107	0.651419949
ENSCAFG00000024148	HEXIM1	20.4613	13.3186	0.000359537	0.0249333	0.650916608
ENSCAFG00000009614	SOX13	7.87934	5.12419	0.00042961	0.0288145	0.650332388
ENSCAFG00000000637	SLC12A2	15.9956	10.3743	0.0002032	0.015679	0.648572107
ENSCAFG00000019730	SLC25A33	5.48284	3.55498	0.000773709	0.0451012	0.648382955
ENSCAFG00000002628	EPS8L1	11.5555	7.48929	0.000253464	0.0187538	0.648114751
ENSCAFG00000018925	KDM4B	4.1272	2.67235	0.000320283	0.0227483	0.647497092
ENSCAFG00000010998	CHD2	2.89096	1.8712	0.000332223	0.0234546	0.647259042
ENSCAFG00000012860	RAPH1	5.25456	3.40075	0.000676644	0.0406796	0.647199766
ENSCAFG00000019059	UBXN6	20.4964	13.2293	0.00024723	0.0183648	0.645445054
ENSCAFG00000019997	CMIP	8.21903	5.30462	0.000388291	0.0265743	0.645407061
ENSCAFG00000023780	MUC5B	1.53363	0.988207	0.000697958	0.0417207	0.644358157
ENSCAFG00000007522	KDM3A	3.44785	2.21844	0.000473382	0.0308905	0.643427063
ENSCAFG00000011659	ATP9A	5.2831	3.39575	0.000161414	0.0131663	0.642757093
ENSCAFG00000016007	KRT10	41.5606	26.6679	0.000199865	0.0155341	0.641663017
ENSCAFG00000018008	FAM59A	8.26352	5.29896	0.00023851	0.0178156	0.641247313
ENSCAFG00000012844	IL10RA	11.8069	7.57088	0.000247055	0.0183648	0.641225046
ENSCAFG00000019688	RERE	6.95254	4.45362	0.000563716	0.0354082	0.640574524
ENSCAFG00000024385	GPR56	4.35169	2.7862	0.000269979	0.0196812	0.640257004
ENSCAFG00000017080	EPN3	6.91475	4.42328	0.000144667	0.0119876	0.639687624
ENSCAFG00000006959	PROM2	9.92813	6.34428	0.000125687	0.0106496	0.639020641
ENSCAFG00000029346	ENSCAFG00000029346	33.052	21.0888	0.000223341	0.0169384	0.638049135
ENSCAFG00000031298	ZKSCAN1	3.80591	2.42736	0.000663901	0.0400673	0.637787021
ENSCAFG00000013261	PCMTD2	6.33348	4.03698	0.000456605	0.0300777	0.637403134
ENSCAFG00000006229	PCDH1	15.3166	9.7582	0.000108271	0.00937683	0.637099617
ENSCAFG00000015840	ATP13A2	4.64929	2.962	0.000365019	0.0252019	0.637086523
ENSCAFG00000007637	ACSS2	10.5717	6.73406	0.00084033	0.0482071	0.636989321
ENSCAFG00000009696	WFDC2	34.6695	22.0677	0.00013896	0.0115657	0.636516246
ENSCAFG00000025105	ENSCAFG00000025105	559.864	356.359	0.000635275	0.0386876	0.636509938
ENSCAFG00000023409	KRT36	14.8206	9.43175	0.000187211	0.0148781	0.636394613
ENSCAFG00000002154	TLN1	6.86681	4.36506	0.000147148	0.0121717	0.635675081
ENSCAFG00000007416	WEE1	4.79593	3.04773	0.000785473	0.045589	0.635482586
ENSCAFG00000000691	B1H0W0_CANFA	3.3515	2.1261	0.000128249	0.0108374	0.634372669
ENSCAFG00000001630	MYH9	18.0857	11.4689	0.000187308	0.0148781	0.634141891
ENSCAFG00000004254	FAAH	5.19123	3.29019	0.000538034	0.0341143	0.63379777

ENSCAFG00000002808	ENSCAFG00000002808	10.7957	6.83403	0.000447997	0.0296458	0.633032596
ENSCAFG00000009978	ETS2	7.8823	4.98598	0.000100158	0.0087846	0.63255395
ENSCAFG00000029519	LDLRAP1	4.11638	2.59971	0.000136547	0.011385	0.631552481
ENSCAFG00000008716	ENSCAFG00000008716	12.1043	7.60419	0.000354836	0.0246679	0.628222202
ENSCAFG00000008404	CLUS_CANFA	158.305	99.4483	0.000208111	0.0159899	0.628206942
ENSCAFG00000004905	ENSCAFG00000004905	26.9168	16.9055	7.87E-05	0.00717501	0.628065
ENSCAFG00000012991	ARI5B_CANFA	2.9509	1.85164	0.000494894	0.0319394	0.627483141
ENSCAFG00000018012	TRPV2	1.84695	1.15782	0.000611725	0.0376437	0.626882157
ENSCAFG00000012430	BCL9L	9.5912	5.99002	5.73E-05	0.00545176	0.624532905
ENSCAFG00000016004	A3RF35_CANFA	60.1865	37.5754	6.85E-05	0.00636296	0.624316084
ENSCAFG00000002494	GALNT12	3.75144	2.34002	0.000591137	0.0366962	0.623765807
ENSCAFG00000019295	MNT	3.64607	2.27176	0.000151297	0.01246	0.623070868
ENSCAFG00000002030	UNC13B	2.67063	1.66385	0.000173002	0.0139405	0.62301779
ENSCAFG00000023449	KRT13	213.042	132.439	0.000194814	0.0152805	0.621656763
ENSCAFG00000018129	MUC16	6.41531	3.98388	4.91E-05	0.00479354	0.620995712
ENSCAFG00000023529	KRT15	188.905	117.233	0.000224606	0.0169651	0.620592361
ENSCAFG00000016180	KLF3	7.71309	4.78654	5.06E-05	0.00490517	0.620573596
ENSCAFG00000032583	METTL7A	16.9624	10.5205	4.14E-05	0.0041524	0.620224732
ENSCAFG00000007603	FLNB	21.8683	13.5607	0.000100217	0.0087846	0.620107644
ENSCAFG00000016817	KDM6B	2.28255	1.41455	0.000285463	0.0206498	0.619723555
ENSCAFG00000019181	INPP5K	3.74889	2.32232	0.00012588	0.0106563	0.619468696
ENSCAFG00000016012	KRT27	59.6468	36.9344	4.75E-05	0.00467521	0.619218466
ENSCAFG00000009632	TYRO3	5.45962	3.38003	0.000753489	0.0442796	0.619096201
ENSCAFG00000013420	ETV5	4.21521	2.6075	0.000154438	0.0126632	0.618593142
ENSCAFG00000016014	KRT26	20.5128	12.6787	4.98E-05	0.00485511	0.618087243
ENSCAFG00000017262	MYH10	4.18073	2.57796	3.78E-05	0.00387403	0.616629153
ENSCAFG00000015669	ZMIZ1	3.8233	2.35698	0.000111909	0.00965633	0.616477912
ENSCAFG00000007749	MYOF	12.3146	7.59002	4.74E-05	0.0046658	0.616343202
ENSCAFG00000017043	ZMYM3	1.17172	0.719331	0.000615261	0.037795	0.61391032
ENSCAFG00000001811	ZFAND5	26.8197	16.4564	0.000122342	0.0104132	0.613593739
ENSCAFG00000011197	O97657_CANFA	1.53851	0.943331	0.000533366	0.0338641	0.613145836
ENSCAFG00000015654	PAPSS2	12.9475	7.93102	3.03E-05	0.00319796	0.61255223
ENSCAFG00000005233	ASB13	2.05437	1.25732	0.000599609	0.0370925	0.612022177
ENSCAFG00000019429	MKNK2	8.62125	5.27602	0.000203457	0.015679	0.611978541
ENSCAFG00000012667	CYBRD1	9.25109	5.65244	0.000796616	0.0461218	0.611002595

ENSCAFG00000014076	CTSH	49.6305	30.3129	2.61E-05	0.00281061	0.610771602
ENSCAFG00000006985	Q2EFX7_CANFA	5.92407	3.61326	4.33E-05	0.0043353	0.609928647
ENSCAFG00000017656	PSTPIP2	3.00559	1.83311	0.000346035	0.0242294	0.609900219
ENSCAFG00000029590	FAM8A1	3.76708	2.29626	6.16E-05	0.00579269	0.609559659
ENSCAFG00000000285	CITED2	10.9682	6.68391	0.000635876	0.0386991	0.609389873
ENSCAFG00000014386	SLC25A42	1.9165	1.16776	0.000541483	0.0342406	0.609319071
ENSCAFG00000008061	ARHGEF3	9.33251	5.68068	2.38E-05	0.00259729	0.608697982
ENSCAFG00000009738	SEMA4G	7.32423	4.44813	0.000212512	0.0162747	0.607317083
ENSCAFG00000016984	PER1	1.80365	1.09526	0.000259861	0.0191217	0.607246417
ENSCAFG00000007953	NEIL2	2.94508	1.78602	0.000303114	0.0217259	0.60644193
ENSCAFG00000005607	SPTLC3	9.40854	5.70146	1.73E-05	0.00196612	0.605987752
ENSCAFG00000012100	CBL	2.35919	1.42748	0.000458472	0.0301057	0.60507208
ENSCAFG00000003845	ENSCAFG00000003845	3.68371	2.22725	0.000456765	0.0300777	0.604621428
ENSCAFG00000007489	RILPL2	4.2755	2.58459	0.000784311	0.0455625	0.604511753
ENSCAFG00000029357	NCEH1	2.83362	1.70709	7.28E-05	0.00672974	0.6024414
ENSCAFG00000031642	ENSCAFG00000031642	5.33338	3.20774	0.000810726	0.0467658	0.601445987
ENSCAFG00000020144	FUK	2.25561	1.35608	0.000290397	0.0209744	0.601203222
ENSCAFG00000014592	NBR1	19.1963	11.5341	1.49E-05	0.00173731	0.600850164
ENSCAFG00000000282	HECA	5.04051	3.02778	0.000404931	0.0275526	0.600689216
ENSCAFG00000014597	ITGAV	14.3362	8.59598	1.18E-05	0.00142476	0.599599615
ENSCAFG00000008557	A4_CANFA	28.0097	16.7813	2.14E-05	0.00235993	0.599124589
ENSCAFG00000023806	KRT25	39.3974	23.5817	1.25E-05	0.00149651	0.598559803
ENSCAFG00000023139	NFE2L1	26.6212	15.9308	1.08E-05	0.00132122	0.598425315
ENSCAFG00000014773	Q6TYZ5_CANFA	13.4081	8.0232	8.38E-05	0.0075765	0.598384559
ENSCAFG00000016761	CPEB4	4.56966	2.73193	0.00044767	0.029645	0.597840977
ENSCAFG00000011146	ABHD4	6.31484	3.7751	0.000423853	0.0285302	0.597814038
ENSCAFG00000004033	PDZK1IP1	29.1344	17.4156	2.94E-05	0.00311419	0.597767587
ENSCAFG00000011522	IRF2BP2	15.0743	9.00089	2.93E-05	0.00310951	0.59710169
ENSCAFG00000007105	TENC1	5.71842	3.41329	2.66E-05	0.00286014	0.596893897
ENSCAFG00000012014	SH3BP4	8.10589	4.82443	1.42E-05	0.00167376	0.595175854
ENSCAFG00000017107	PLEKHO2	3.59712	2.1381	7.76E-05	0.00708256	0.59439218
ENSCAFG00000017329	FLRT2	3.72332	2.21077	0.000261765	0.0192467	0.593763093
ENSCAFG00000001906	FZD1	10.8127	6.4188	3.81E-05	0.00389089	0.593635262
ENSCAFG00000002021	ENPP5	4.09635	2.4304	0.000457603	0.0301057	0.593308677
ENSCAFG00000011171	ENSCAFG00000011171	10.7745	6.37459	7.35E-05	0.00677935	0.591636735

ENSCAFG00000017130	NUPR1	128.542	75.995	4.03E-05	0.00406302	0.591207543
ENSCAFG00000014134	FZD2	15.1132	8.93123	2.07E-05	0.002289	0.590955588
ENSCAFG00000009175	UBTD1	8.02957	4.73997	0.000345971	0.0242294	0.590314301
ENSCAFG00000013926	FBXO33	5.48835	3.23642	0.000184526	0.0147193	0.589689069
ENSCAFG00000012082	SEMA4B	9.80424	5.76019	7.25E-06	0.000926027	0.587520297
ENSCAFG00000004481	SC6A6_CANFA	18.2663	10.7261	5.97E-06	0.000785367	0.587207042
ENSCAFG00000004991	PPARG_CANFA	19.4507	11.4133	5.85E-06	0.000775748	0.586780938
ENSCAFG00000000843	UPK3A	18.9439	11.1142	2.97E-05	0.00314241	0.586690175
ENSCAFG00000028971	CCDC85C	3.77393	2.20973	0.000258001	0.0190295	0.585524904
ENSCAFG00000008198	KIF13B	3.34861	1.95664	1.42E-05	0.00167376	0.584314089
ENSCAFG00000002028	VLDLR	2.68933	1.5677	0.000708994	0.0422506	0.582933296
ENSCAFG00000002746	KLHL7	4.67376	2.72416	0.000101463	0.00887728	0.58286262
ENSCAFG00000000784	TTC38	0.901411	0.525141	0.000268562	0.0196444	0.582576649
ENSCAFG00000016604	ZSWIM4	2.02828	1.1816	0.000188445	0.0149431	0.582562565
ENSCAFG00000001938	VEGFA_CANFA	21.9462	12.778	4.42E-05	0.00440493	0.582242028
ENSCAFG00000010706	MXI1	6.33579	3.67814	8.02E-06	0.00101702	0.580533761
ENSCAFG00000003156	MYH14	10.5934	6.14653	1.95E-05	0.00216452	0.580222591
ENSCAFG00000006777	ADAMTS9	1.56612	0.908451	2.61E-05	0.00281698	0.580064746
ENSCAFG00000019224	VASN	15.6742	9.07492	1.76E-05	0.00199491	0.578971814
ENSCAFG00000004826	SLC6A9	5.93465	3.42769	0.000866207	0.0494201	0.577572393
ENSCAFG00000019990	IER5L	4.0304	2.32747	0.000665686	0.0401235	0.577478662
ENSCAFG00000029966	ENSCAFG00000029966	0.561923	0.324443	0.000557871	0.0351115	0.577379819
ENSCAFG00000001505	UBE2H	9.03019	5.21349	8.77E-05	0.00784465	0.577340012
ENSCAFG00000017042	JDP2	11.9549	6.89052	0.000327553	0.0231946	0.576376214
ENSCAFG00000019197	TJP3	2.95204	1.69885	0.000353171	0.0246007	0.575483395
ENSCAFG00000015587	KLHDC7A	4.31703	2.4829	5.19E-05	0.00502641	0.57514078
ENSCAFG00000019400	WDR85	3.70588	2.1284	0.000224017	0.0169634	0.574330523
ENSCAFG00000016990	CA12	13.4042	7.697	2.58E-05	0.00278778	0.574223005
ENSCAFG00000015776	CCDC120	4.28035	2.45598	8.61E-05	0.00773195	0.573780182
ENSCAFG00000005940	THSD1	5.14147	2.94543	5.88E-06	0.000777823	0.572877018
ENSCAFG00000001516	TST	2.27729	1.30309	0.000594656	0.0368589	0.57221083
ENSCAFG00000024916	ABCA8	1.74074	0.995849	5.92E-05	0.00559758	0.572083712
ENSCAFG00000011394	KLHL25	7.06417	4.04013	2.08E-05	0.00230124	0.571918569
ENSCAFG00000019305	RAP1GAP2	3.20866	1.83479	6.53E-05	0.00611384	0.571824375
ENSCAFG00000001570	PSAT1	25.9216	14.8194	2.62E-05	0.00282121	0.571700821

ENSCAFG00000001360	CBX7	1.50922	0.862767	0.000175786	0.0141301	0.571664171
ENSCAFG00000006673	PPP1R3B	7.00526	3.99734	0.000128554	0.0108437	0.570619791
ENSCAFG00000015999	KRT23	2.41331	1.37576	0.000165853	0.0134816	0.57007181
ENSCAFG00000001791	ALDH1A1	4.44728	2.53403	0.000393693	0.0269048	0.569793222
ENSCAFG00000010137	MFSD4	3.07688	1.75069	6.78E-06	0.000871516	0.568982216
ENSCAFG00000030441	MGAT3	8.44266	4.79511	1.50E-06	0.000227973	0.567961993
ENSCAFG00000009877	ITGB6	2.6278	1.49248	7.45E-05	0.00685371	0.567957988
ENSCAFG00000019811	SPIRE2	2.53124	1.43671	0.000119192	0.0102004	0.567591378
ENSCAFG00000010052	CATD_CANFA	44.3631	25.1609	1.25E-06	0.000193444	0.567158291
ENSCAFG00000004901	STEAP3	4.01035	2.27353	0.000114007	0.00981037	0.566915606
ENSCAFG00000003966	ANKRD50	6.50004	3.68456	1.14E-06	0.000178169	0.566851896
ENSCAFG00000020370	FHOD1	0.868305	0.491548	0.000425946	0.0286506	0.566100621
ENSCAFG00000008891	SERINC5	12.9744	7.33775	1.00E-06	0.000157909	0.565556018
ENSCAFG00000008980	HERPUD1	12.951	7.31206	8.28E-06	0.00104348	0.56459424
ENSCAFG00000015872	TSPAN14	5.4863	3.09435	6.29E-06	0.00082064	0.564013999
ENSCAFG00000014334	HDAC5	2.96286	1.66426	1.80E-05	0.00202386	0.561707269
ENSCAFG00000009313	PHF21A	1.97447	1.10785	0.000167468	0.0135893	0.561087279
ENSCAFG00000008300	ZNF395	6.32581	3.54585	1.01E-06	0.000159865	0.560536911
ENSCAFG00000031326	CEBPB	10.7149	6.00169	3.98E-05	0.00402152	0.560125619
ENSCAFG00000002017	FAM214B	2.97241	1.65935	1.23E-05	0.00147759	0.558250712
ENSCAFG00000011868	PCK2	21.7214	12.1129	8.27E-07	0.000133439	0.557648218
ENSCAFG00000007298	TCP11L1	3.34141	1.86186	8.63E-05	0.00774281	0.557207885
ENSCAFG00000011913	Q6Q9F8_CANFA	21.7971	12.1252	6.76E-06	0.000870398	0.556275835
ENSCAFG00000002222	ASNS	10.2429	5.68874	4.80E-07	8.23E-05	0.555383729
ENSCAFG00000009125	TMEM67	0.904766	0.502405	0.000259289	0.0190946	0.555287223
ENSCAFG00000008023	IL17RD	3.18329	1.76139	4.18E-05	0.00419245	0.553323763
ENSCAFG00000010271	CARS	4.27478	2.36378	7.27E-07	0.000118762	0.552959451
ENSCAFG00000010213	RIPK4	9.11013	5.02938	5.56E-07	9.37E-05	0.55206457
ENSCAFG00000003210	FHL3	2.83639	1.56373	0.000127534	0.0107867	0.55130994
ENSCAFG00000006757	PPFIBP2	2.23604	1.23048	3.88E-05	0.0039457	0.55029427
ENSCAFG00000007882	PCSK1	2.81068	1.5424	2.05E-06	0.000304259	0.548764
ENSCAFG00000016103	AP5Z1	2.73804	1.50137	9.72E-06	0.00120954	0.548337497
ENSCAFG00000018208	Q8HYR4_CANFA	1.29676	0.710936	0.00010496	0.0091491	0.548240229
ENSCAFG00000016947	FAM117A	2.63556	1.4445	0.00017313	0.0139405	0.548080863
ENSCAFG00000005251	RALGAPA2	0.644027	0.352612	3.97E-05	0.0040201	0.547511207

ENSCAFG00000025580	FRY	0.276409	0.151222	0.000343406	0.0240913	0.547095066
ENSCAFG00000007407	CEBPA	15.1732	8.26236	9.29E-05	0.00823611	0.544536419
ENSCAFG00000012827	TCN2	3.12843	1.70221	5.04E-05	0.00489521	0.544109985
ENSCAFG00000007883	ENSCAFG00000007883	7.15132	3.883	5.00E-05	0.00487043	0.542976681
ENSCAFG00000016802	ZFYVE1	4.35981	2.36588	2.68E-06	0.000387681	0.542656675
ENSCAFG00000008700	FNIP2	9.62037	5.21889	2.25E-06	0.00033096	0.542483293
ENSCAFG00000030662	AHNAK2	36.6678	19.8882	6.30E-07	0.000105044	0.54238869
ENSCAFG00000004840	INHBB	5.64473	3.05734	0.000153607	0.0126171	0.541627323
ENSCAFG00000011246	ALDH3B2	6.90249	3.73797	1.22E-05	0.00146804	0.541539358
ENSCAFG00000028550	ZNF814	2.16386	1.1707	3.00E-05	0.00317488	0.541023911
ENSCAFG00000005621	BHLHE40	16.531	8.94316	1.61E-07	3.03E-05	0.540993285
ENSCAFG00000002169	NFIL3	8.11476	4.38413	4.56E-06	0.000624252	0.540266132
ENSCAFG00000006157	MAVS	12.8761	6.93149	1.26E-05	0.0015052	0.538322163
ENSCAFG00000019022	ACOT11	1.3429	0.722902	1.29E-05	0.00153431	0.538314096
ENSCAFG00000030537	HSPB8	21.1784	11.3547	9.11E-08	1.76E-05	0.536145318
ENSCAFG00000006966	ST5	1.16754	0.625944	2.35E-05	0.00257528	0.536122103
ENSCAFG00000005021	ENSCAFG00000005021	3.95437	2.11781	1.66E-06	0.000251831	0.535561923
ENSCAFG00000015014	SLC7A4	0.407622	0.218134	0.000180704	0.0144512	0.535137946
ENSCAFG00000013888	ENSCAFG00000013888	0.866105	0.462899	0.000419713	0.0283735	0.534460602
ENSCAFG00000010599	HYAL1	6.37379	3.40475	7.20E-06	0.000920356	0.534179821
ENSCAFG00000015203	PLEKHG6	8.73391	4.65075	7.04E-06	0.000902664	0.532493465
ENSCAFG00000011405	TXNIP	20.3838	10.79	4.87E-08	9.94E-06	0.529341928
ENSCAFG00000030095	ENSCAFG00000030095	6.25857	3.30447	2.85E-06	0.000408577	0.527991218
ENSCAFG00000024839	KIF21B	0.190105	0.100308	0.000574762	0.0358862	0.527645249
ENSCAFG00000002393	SEMA4C	3.46208	1.82601	1.29E-06	0.000199473	0.527431486
ENSCAFG00000012765	CYP20A1	4.09223	2.15282	2.09E-05	0.002305	0.526075025
ENSCAFG00000009385	CHST1	1.40576	0.73923	0.000267097	0.0195775	0.525857899
ENSCAFG00000005104	ITPKC	2.49117	1.3051	1.46E-05	0.00170771	0.523890381
ENSCAFG00000029187	RHOV	1.44591	0.755936	1.29E-05	0.00152759	0.522809857
ENSCAFG00000014771	ENSCAFG00000014771	4.98854	2.6021	4.50E-05	0.00447643	0.521615543
ENSCAFG00000003939	HBP1	12.97	6.73833	1.71E-07	3.19E-05	0.519531997
ENSCAFG00000006965	TRIB3	15.9082	8.25572	3.66E-08	7.66E-06	0.518960033
ENSCAFG00000019444	EXD3	0.212838	0.110332	0.000557508	0.0351115	0.518384875
ENSCAFG00000000767	CELSR1	0.968673	0.498251	1.09E-05	0.00132949	0.514364497
ENSCAFG00000014106	ATG2A	1.56376	0.802298	4.97E-06	0.000670613	0.513056991

ENSCAFG00000013668	ENSCAFG00000013668	1.11991	0.573787	5.52E-05	0.00528501	0.512350992
ENSCAFG00000006446	ULK1	6.33577	3.24088	0.000130438	0.0109829	0.511521094
ENSCAFG00000008104	ENSCAFG00000008104	7.40467	3.78068	1.22E-05	0.00146804	0.510580485
ENSCAFG00000012095	AHDC1	1.42132	0.725499	4.34E-06	0.000596442	0.510440295
ENSCAFG00000001448	PIM1	6.29989	3.19612	6.03E-08	1.21E-05	0.507329493
ENSCAFG00000015239	C1orf114	0.804601	0.407154	0.000436708	0.0291657	0.506032182
ENSCAFG00000011354	ENSCAFG00000011354	4.33734	2.18668	3.82E-05	0.00389835	0.504152315
ENSCAFG00000023922	AQP3	2.10968	1.06045	1.23E-05	0.00147832	0.502659171
ENSCAFG00000014374	NYAP1	1.37564	0.691266	0.000243224	0.0181245	0.502505016
ENSCAFG00000006904	SLC52A3	1.94279	0.973975	7.49E-07	0.000121949	0.501327987
ENSCAFG00000018726	BCORL1	0.422735	0.211861	0.000406018	0.0276066	0.501167398
ENSCAFG00000017069	EFNA1	12.5652	6.29381	2.98E-06	0.000425453	0.500892147
ENSCAFG00000000097	ERBB3	13.0461	6.50901	3.00E-09	7.20E-07	0.498923816
ENSCAFG00000031339	TMEM86A	0.669062	0.333091	1.55E-05	0.00179126	0.497847733
ENSCAFG00000000967	ZHX2	0.400996	0.199544	0.000754082	0.0442868	0.497620924
ENSCAFG00000006665	CLIP3	1.62802	0.809495	3.80E-05	0.00388485	0.497226693
ENSCAFG00000000360	SQSTM1	64.2924	31.8843	2.55E-09	6.22E-07	0.495926424
ENSCAFG00000000365	C5orf45	16.7855	8.32265	3.49E-09	8.33E-07	0.495823776
ENSCAFG00000019937	KIAA0513	3.4021	1.68533	4.11E-06	0.000569799	0.495379325
ENSCAFG00000018414	ENSCAFG00000018414	2.43584	1.20611	9.87E-05	0.00869108	0.49515157
ENSCAFG00000029093	LYPD6	0.524371	0.258575	0.000200826	0.0155702	0.493114608
ENSCAFG00000003180	SLC1A4	6.42688	3.16369	5.11E-09	1.19E-06	0.492259074
ENSCAFG00000008395	SCARA3	2.55787	1.25863	9.22E-08	1.78E-05	0.492061755
ENSCAFG00000014620	VAT1	25.4643	12.5051	2.78E-08	5.94E-06	0.491083595
ENSCAFG00000031672	C14orf79	1.92018	0.942777	3.04E-05	0.00320201	0.490983658
ENSCAFG00000011433	CEBPE	1.5162	0.742116	1.55E-05	0.00178983	0.489457855
ENSCAFG00000005714	CXXC5	18.1001	8.83536	2.69E-08	5.76E-06	0.48813874
ENSCAFG00000003300	RNF144A	1.47294	0.716812	0.000195743	0.0153023	0.486653903
ENSCAFG00000018146	ALDH3A1	1.16546	0.563449	5.28E-05	0.00510225	0.483456318
ENSCAFG00000015925	CCPG1	3.98377	1.92285	3.41E-08	7.22E-06	0.482670937
ENSCAFG00000000569	ZNF608	1.08128	0.517764	3.72E-07	6.52E-05	0.478843593
ENSCAFG00000029053	TRIB2_CANFA	6.00285	2.8572	1.95E-09	4.85E-07	0.475973912
ENSCAFG00000018622	DAB2	1.08089	0.51394	1.97E-06	0.00029454	0.475478541
ENSCAFG00000020184	CHST4	15.6659	7.35483	6.46E-10	1.72E-07	0.469480209
ENSCAFG00000030761	ZNF835	0.843232	0.395011	0.000269227	0.0196722	0.468448778

ENSCAFG00000012085	ABCC5	0.883488	0.413494	1.22E-07	2.33E-05	0.468024467
ENSCAFG00000012703	ENSCAFG00000012703	2.47761	1.15911	5.43E-06	0.000727173	0.467833921
ENSCAFG00000005595	FARP1	0.976246	0.45586	1.99E-06	0.00029655	0.466951977
ENSCAFG00000017411	EPOR	1.58438	0.737694	0.000397564	0.0271102	0.465604211
ENSCAFG00000016107	NOTCH3	1.01974	0.471117	2.37E-07	4.34E-05	0.461997176
ENSCAFG00000012129	GRK5	0.857347	0.394718	3.65E-07	6.41E-05	0.460394683
ENSCAFG00000017626	C5orf4	1.12134	0.515552	0.000210204	0.0161243	0.459764211
ENSCAFG00000004655	BCL3	3.88186	1.7587	9.33E-07	0.000148842	0.453056009
ENSCAFG00000016461	FBXL20	2.32615	1.05273	1.32E-06	0.000204016	0.452563248
ENSCAFG00000004797	USP35	0.464749	0.210023	8.97E-05	0.0079861	0.451906298
ENSCAFG00000012390	NYNRIN	1.16885	0.524509	1.31E-08	2.90E-06	0.448739359
ENSCAFG00000010752	C2orf72	2.19018	0.968818	0.000295309	0.0212964	0.442346291
ENSCAFG00000017095	YPEL3	22.546	9.94227	8.43E-10	2.21E-07	0.440977113
ENSCAFG00000013831	HEXIM2	2.34643	1.03374	8.61E-06	0.00108271	0.440558636
ENSCAFG00000005048	IFT172	0.206883	0.0906088	5.67E-05	0.00539734	0.43797122
ENSCAFG00000005593	NCCRP1	1.23496	0.540469	1.71E-05	0.00194657	0.437640895
ENSCAFG00000006674	SPINK5	33.9147	14.8229	1.88E-10	5.34E-08	0.437064164
ENSCAFG00000030682	ENSCAFG00000030682	2.45771	1.07141	4.82E-05	0.00473038	0.435938333
ENSCAFG00000009061	SLC24A6	1.0222	0.445243	8.66E-05	0.00775983	0.435573273
ENSCAFG00000031154	KIAA1161	0.674649	0.292146	7.12E-05	0.00660043	0.433034067
ENSCAFG00000016798	CREBRF	0.874011	0.377611	4.07E-06	0.000565067	0.432043761
ENSCAFG00000008685	CCNG2	6.58725	2.84559	5.20E-11	1.55E-08	0.431984516
ENSCAFG00000013651	UPK3B	3.47726	1.4929	3.92E-10	1.07E-07	0.42933229
ENSCAFG00000029920	TSC22D3	2.26476	0.96516	6.15E-10	1.64E-07	0.426164362
ENSCAFG00000006759	KIAA1456	0.23482	0.0998194	0.000280833	0.0203462	0.425089004
ENSCAFG00000003749	SLC7A11	4.07679	1.73037	4.51E-08	9.25E-06	0.424444232
ENSCAFG00000017619	OTUB2	1.45273	0.615094	0.000191113	0.0150472	0.423405588
ENSCAFG00000002727	ABCA1	1.62337	0.683423	7.81E-11	2.28E-08	0.420990286
ENSCAFG00000001957	DOCK8	0.234599	0.0977347	4.57E-06	0.000624326	0.416603225
ENSCAFG00000007475	SORBS2	0.483843	0.201331	4.67E-05	0.00462323	0.416108118
ENSCAFG00000016700	RORA	0.162946	0.0676833	0.000540817	0.0342215	0.415372577
ENSCAFG00000016376	DHRS3	1.11933	0.464217	4.34E-05	0.0043353	0.41472756
ENSCAFG00000031759	NAPRT1	3.3128	1.36176	1.79E-08	3.92E-06	0.41106013
ENSCAFG00000012682	PELI3	0.77682	0.319245	0.000138737	0.0115574	0.41096393
ENSCAFG00000005428	DYRK1B	2.7792	1.13792	6.64E-09	1.51E-06	0.409441566

ENSCAFG00000013923	SUSD2	1.00305	0.409869	3.92E-05	0.00397715	0.408622701
ENSCAFG00000016328	GRB7	5.83107	2.37294	4.37E-12	1.46E-09	0.40694761
ENSCAFG00000032469	DDIT4	20.9744	8.48208	2.62E-14	1.04E-11	0.404401556
ENSCAFG00000005395	GDPD5	0.88538	0.356854	3.96E-07	6.87E-05	0.403051797
ENSCAFG00000005284	C17orf99	1.37122	0.548499	0.000267939	0.0196238	0.400008022
ENSCAFG00000010195	FBXO25	1.36631	0.546405	5.97E-06	0.000785367	0.399912904
ENSCAFG00000009414	CHAC1	6.86317	2.69733	5.82E-09	1.34E-06	0.393015181
ENSCAFG00000002147	IL1R1	2.02765	0.788512	6.14E-08	1.23E-05	0.388879738
ENSCAFG00000015579	GPA33	0.216603	0.0842096	0.000364104	0.025158	0.388773932
ENSCAFG00000017937	CYP1A1	20.6017	7.99663	6.66E-16	2.94E-13	0.38815389
ENSCAFG00000008484	ENSCAFG00000008484	0.148775	0.0577349	6.12E-05	0.00576523	0.38806856
ENSCAFG00000017891	DEGS2	18.756	7.27124	1.82E-12	6.30E-10	0.387675411
ENSCAFG00000012375	NFATC4	3.42114	1.32626	2.17E-08	4.72E-06	0.3876661
ENSCAFG00000017941	CYP1A2	8.95607	3.47083	6.35E-13	2.28E-10	0.387539401
ENSCAFG00000003044	FKBP14	1.8378	0.706621	6.84E-05	0.00636084	0.384492872
ENSCAFG00000001974	KANK1	0.190348	0.0727863	3.54E-05	0.00365706	0.38238542
ENSCAFG00000004082	ABTB1	1.98666	0.753537	2.53E-10	7.08E-08	0.37929842
ENSCAFG00000019194	SLC43A2	1.40432	0.525125	8.90E-08	1.73E-05	0.373935428
ENSCAFG00000012580	ENSCAFG00000012580	0.449537	0.165699	1.67E-05	0.00190406	0.368599248
ENSCAFG00000029146	PPP1R3C	4.2054	1.52178	2.88E-13	1.06E-10	0.361863319
ENSCAFG00000012255	RAB17	1.31399	0.47212	7.50E-06	0.000952518	0.359302582
ENSCAFG00000006699	CALCOCO1	2.08378	0.746444	4.59E-13	1.66E-10	0.358216318
ENSCAFG00000030515	YPEL2	8.16142	2.91339	5.25E-07	8.94E-05	0.356970968
ENSCAFG00000003152	ZNF672	0.930733	0.331975	4.04E-05	0.00407146	0.356681239
ENSCAFG00000005747	C8orf4	12.5845	4.37437	5.52E-09	1.28E-06	0.347599825
ENSCAFG00000019675	DECR2	1.79052	0.605726	1.52E-06	0.000230347	0.338296137
ENSCAFG00000014873	MXD4	1.66164	0.555023	1.41E-06	0.0002162	0.334021208
ENSCAFG00000016366	C1orf158	1.08418	0.349569	0.000510095	0.0327405	0.322427088
ENSCAFG00000002057	PDGFRA	0.213363	0.06829	6.23E-08	1.25E-05	0.320064866
ENSCAFG00000010807	PDCD4	2.43816	0.754467	3.45E-08	7.28E-06	0.309441136
ENSCAFG00000019445	NRARP	6.26902	1.92233	5.46E-07	9.27E-05	0.306639634
ENSCAFG00000029558	BMF	2.39258	0.697076	0	0	0.291349088
ENSCAFG00000001007	FBXO32	0.947766	0.267883	3.75E-08	7.81E-06	0.282646771
ENSCAFG00000013077	PIK3IP1	1.7711	0.483944	4.21E-08	8.71E-06	0.273244876
ENSCAFG00000019824	DPEP1	0.247481	0.0568279	0.000756233	0.0443853	0.229625305

ENSCAFG00000011850	KLHL24	5.34059	1.21806	0	0	0.228075924
ENSCAFG00000009418	TP53INP1	1.21425	0.273081	1.67E-11	5.26E-09	0.22489685
ENSCAFG00000029904	HBA_CANFA	0.376013	0.0596974	4.61E-05	0.00457591	0.158764192
ENSCAFG00000030131	ENSCAFG00000030131	0.305328	0	8.50E-05	0.00766604	0
ENSCAFG00000031741	ENSCAFG00000031741	0.462933	0	1.31E-05	0.00155425	0

#DIV/0!, Indicates that no accurate fold change can be calculated as the denominator is 0.

SUPPLEMENTARY TABLE 3. Differentially expressed genes in MDCK cells infected with virus 65 vs cells infected with virus 85.

gene_id	gene	value_1 (65)	value_2 (85)	p_value	q_value	FOLD CHANGE 65 VS 85
ENSCAFG00000000517	TNFA_CANFA	0	0.228953	0.000492312	0.0318383	#DIV/0!
ENSCAFG00000000803	DLA-DRA	0	0.159486	0.000492312	0.0318383	#DIV/0!
ENSCAFG00000018164	CCL4_CANFA	0.0471098	0.675799	9.07E-06	0.0011344	14.34518932
ENSCAFG00000002733	Q95LE4_CANFA	0.0324374	0.305077	0.000226062	0.0170348	9.405100285
ENSCAFG00000005582	ENSCAFG00000005582	1.16403	9.78473	0	0	8.405908782
ENSCAFG00000011481	IL29L	3.29086	26.054	0	0	7.917079426
ENSCAFG00000005588	ENSCAFG00000005588	0.715677	5.40374	0	0	7.550529079
ENSCAFG00000001653	IFNB1	2.54138	12.7543	0	0	5.018651284
ENSCAFG00000018171	CCL5	2.59183	12.71	0	0	4.90387101
ENSCAFG00000032243	DIO2	0.0415924	0.173371	0.000731879	0.0431988	4.168333638
ENSCAFG00000020200	GBP6	0.485786	1.80615	0	0	3.717995167
ENSCAFG00000012046	IFI6	1.58598	5.74363	1.07E-11	3.45E-09	3.621502163
ENSCAFG00000019809	GSTM3	0.357181	1.18189	1.23E-05	0.00147805	3.308938605
ENSCAFG00000002120	CD274	0.192439	0.624459	3.65E-06	0.000509008	3.244971134
ENSCAFG00000008584	CXL10_CANFA	96.6783	311.781	0	0	3.224932586
ENSCAFG00000024116	ENSCAFG00000024116	1.53478	4.81009	3.42E-06	0.000481938	3.134058302
ENSCAFG00000018405	FST	2.11756	6.61268	2.22E-16	1.01E-13	3.122782826
ENSCAFG00000006550	ENSCAFG00000006550	1.76456	5.46544	4.37E-10	1.19E-07	3.097338713
ENSCAFG00000008391	HEY1	0.440095	1.31319	2.73E-09	6.61E-07	2.983878481
ENSCAFG00000000267	TNFAIP3	3.85566	10.5727	0	0	2.742124565
ENSCAFG00000020204	ENSCAFG00000020204	1.35786	3.5978	6.75E-07	0.000111732	2.649610416
ENSCAFG00000013418	NFKBIA	6.10206	15.8084	8.22E-15	3.34E-12	2.590666103
ENSCAFG00000008285	HNF4G	0.262921	0.672163	2.21E-07	4.06E-05	2.55652078
ENSCAFG00000009617	IFIT1	23.2887	57.8476	3.31E-14	1.31E-11	2.483934269
ENSCAFG000000139	Q7YSA1_CANFA	2.01528	4.83915	2.02E-11	6.26E-09	2.401229606

09						
ENSCAFG00000003880	ENSCAFG00000003880	27.6122	61.9422	4.72E-10	1.28E-07	2.243291009
ENSCAFG00000012339	ATF3	3.77166	8.42425	0.000382272	0.026258	2.233565592
ENSCAFG00000024540	GBP5	1.84438	4.11102	1.21E-09	3.14E-07	2.228944144
ENSCAFG00000003550	INHBA	2.01736	4.46952	6.32E-09	1.45E-06	2.215529206
ENSCAFG00000006212	SEMA3A	0.77038	1.65008	1.90E-06	0.000285477	2.141903995
ENSCAFG00000003029	IL8_CANFA	1.45821	3.01756	1.49E-06	0.000227973	2.069359009
ENSCAFG00000031614	IFIT3	27.2511	54.1892	3.90E-09	9.18E-07	1.988514225
ENSCAFG00000005750	IDO1	16.3442	31.3856	2.35E-08	5.09E-06	1.920289766
ENSCAFG00000015087	RARRES3	8.89658	17.0199	1.52E-07	2.86E-05	1.913083455
ENSCAFG00000032746	MXD1	1.86389	3.48984	5.54E-06	0.000738822	1.872342252
ENSCAFG00000000851	IRF1	9.35472	17.4592	8.77E-08	1.71E-05	1.866351959
ENSCAFG00000000498	ENSCAFG00000000498	18.0162	33.5151	6.07E-05	0.00572312	1.860275752
ENSCAFG00000012657	IRGM/IFI1	19.8128	36.6909	1.43E-07	2.69E-05	1.851878584
ENSCAFG00000001807	DDX58	53.5688	98.9914	8.39E-07	0.000134937	1.847930138
ENSCAFG00000022709	ENSCAFG00000022709	181.776	334.125	6.57E-07	0.000108806	1.838113942
ENSCAFG00000009612	IFIT2	6.69669	12.2205	2.67E-07	4.80E-05	1.824856758
ENSCAFG00000007501	DBR1	4.56701	8.28592	2.08E-06	0.00030719	1.814298633
ENSCAFG00000019054	ENSCAFG00000019054	103.42	181.418	1.50E-06	0.000228183	1.754186811
ENSCAFG00000010588	MARCKSL1	64.7839	111	3.98E-06	0.000552739	1.713388666
ENSCAFG00000000686	ENSCAFG00000000686	5.24686	8.9407	0.000269747	0.0196796	1.704009636
ENSCAFG00000014017	BATF2	3.80272	6.47318	0.000572323	0.0357576	1.702249968
ENSCAFG00000030839	PNRC1	6.60079	11.1868	6.48E-06	0.000838941	1.694766839
ENSCAFG00000000497	ENSCAFG00000000497	12.4006	21.0005	6.39E-06	0.000829749	1.693506766
ENSCAFG00000015105	BIRC3_CANFA	2.55616	4.31647	3.67E-05	0.00376919	1.688654075
ENSCAFG00000007539	ADML_CANFA	11.1495	18.1918	5.81E-05	0.00551317	1.631624737
ENSCAFG00000028671	ENSCAFG00000028671	11.6463	19.0004	5.34E-05	0.00514409	1.631453766
ENSCAFG00000004176	PANX1	4.66497	7.59471	3.17E-05	0.00331793	1.628029762
ENSCAFG00000029359	KCNK5	4.08031	6.61047	7.37E-05	0.00679158	1.62009014
ENSCAFG00000028829	ENSCAFG00000028829	11.8413	19.0353	9.09E-05	0.00808429	1.607534646
ENSCAFG00000017539	LDLR	20.6087	32.7792	0.000120719	0.0102936	1.590551563
ENSCAFG00000031999	SLC30A1	6.11372	9.5159	0.000155419	0.0127326	1.556482796
ENSCAFG00000016138	FBLIM1	7.59099	4.96182	0.000502789	0.0323378	0.653645967
ENSCAFG00000011913	Q6Q9F8_CANFA	18.8785	12.1252	0.000665214	0.0401208	0.642275605
ENSCAFG00000019224	VASN	14.2793	9.07492	0.000390172	0.0266836	0.635529753

ENSCAFG00000016328	GRB7	3.73522	2.37294	0.000650389	0.0394037	0.635287881
ENSCAFG00000013651	UPK3B	2.37344	1.4929	0.000813966	0.0469239	0.629002629
ENSCAFG00000017095	YPEL3	15.8913	9.94227	0.000591251	0.0366962	0.625642333
ENSCAFG00000031382	PMEPA1	12.6759	7.62911	1.68E-05	0.00191468	0.601859434
ENSCAFG00000001906	FZD1	10.6806	6.4188	6.18E-05	0.0058112	0.600977473
ENSCAFG00000014134	FZD2	14.9568	8.93123	3.21E-05	0.00334853	0.597135082
ENSCAFG00000030095	ENSCAFG00000030095	5.60289	3.30447	0.000128479	0.0108437	0.58977956
ENSCAFG00000032422	RARRES1	8.41941	4.80906	0.000416876	0.028202	0.571187292
ENSCAFG00000004082	ABTB1	1.34361	0.753537	0.000250843	0.0185744	0.560830152
ENSCAFG00000012375	NFATC4	2.40963	1.32626	0.000603133	0.0372369	0.550399854
ENSCAFG00000006699	CALCOCO1	1.35923	0.746444	4.08E-05	0.00410076	0.549166808
ENSCAFG00000008395	SCARA3	2.30722	1.25863	5.71E-06	0.000759279	0.545517983
ENSCAFG00000011850	KLHL24	2.23515	1.21806	0.000123555	0.0104974	0.544956714
ENSCAFG00000031339	TMEM86A	0.69534	0.333091	4.87E-06	0.000659195	0.479033279
ENSCAFG00000010807	PDCD4	1.66604	0.754467	0.000264513	0.0194183	0.452850472
ENSCAFG00000002057	PDGFRA	0.152917	0.06829	0.000190654	0.0150472	0.446582133
ENSCAFG00000019675	DECR2	1.44471	0.605726	0.000159217	0.0130097	0.419271688
ENSCAFG00000029558	BMF	1.6788	0.697076	3.13E-10	8.62E-08	0.415222778
ENSCAFG00000025031	ENSCAFG00000025031	0.497476	0.187584	0.000218161	0.0166261	0.377071457
ENSCAFG00000028395	SNORA31	27.752	0	1.80E-05	0.00202447	0

#DIV/0!, Indicates that no accurate fold change can be calculated as the denominator is 0.

SUPPLEMENTARY TABLE 4. Aminoacid changes between viruses isolated from patients 65 and 85.

GENE	VIRUS 65	VIRUS 85
HA	144T	144A
HA	158R	158G
HA	161N	161S
HA	173S	173L
HA	277R	277Q
HA	363M	363V
NA	43N	43D
NA	197N	197D
NA	296K	296R
PA-X	210L	210P
PB1	215R	215K
PB1	587A	587T
PB1	635R	635K
PB2	106A	106T
PB2	122I	122V

PB2	475M	475L
PB2	480L	480V
PB2	740N	740D
NS1	26E	26K
NS1	64I	64T
NS1	224R	224K

SUPPLEMENTARY TABLE 5. Frequency of amino acid changes in MDCK cells infected with viruses isolated from patients 65 and 85.

GENE	SAMPLE 65*	SAMPLE 85*	aa CHANGE
NA	17.94%	45.94%	D151N
NA	35.83%	-	D151G
HA	-	91.53%	D241N
PA	-	92.01%	I421V
PB2	-	98.49%	I63V

*Only frequencies higher than 1% were considered reliable and relevant.

SUPPLEMENTARY TABLE 6. Amino acid changes at postion 64 in NS1 proteins from circulating influenza A viruses

	total number of sequences	aminoacid position 64	number of sequences	%
**human**				
H1N1	9269	I	9263	99.94
		T	2	0.02
		V	2	0.02
		L	2	0.02
pH1N1	5961	I	5926	99.41
		T	7	0.12
		V	18	0.30
		L	10	0.17
H3N2	6980	I	6976	99.94
		T	2	0.03
		V	1	0.015
		L	1	0.015
H5Nx	276	I	276	100
H7Nx	88	I	87	98.86
		V	1	1.14
**Avian**				
H1N1	340	I	336	98.82
		V	4	1.18
H3N2	248	I	248	100
**Swine**				
H1N1	3140	I	3117	99.26
		T	1	0.03
		V	4	0.13
		L	9	0.29
		M	4	0.13

		F	5	0.16
pH1N1	805	I	805	100
H3N2	1017	I	1015	99.8
		V	1	0.1
		M	1	0.1
